# Isolation of a Bent Dysprosium Bis(amide) Single-Molecule Magnet

**DOI:** 10.1021/jacs.3c12427

**Published:** 2024-01-29

**Authors:** Jack Emerson-King, Gemma K. Gransbury, George F. S. Whitehead, Iñigo J. Vitorica-Yrezabal, Mathieu Rouzières, Rodolphe Clérac, Nicholas F. Chilton, David P. Mills

**Affiliations:** † Department of Chemistry, The University of Manchester, Oxford Road, Manchester M13 9PL, U.K.; ‡ Univ. Bordeaux, CNRS, CRPP, UMR 5031, 33600 Pessac, France; § Research School of Chemistry, The Australian National University, Sullivans Creek Road, Canberra, ACT 2601, Australia

## Abstract

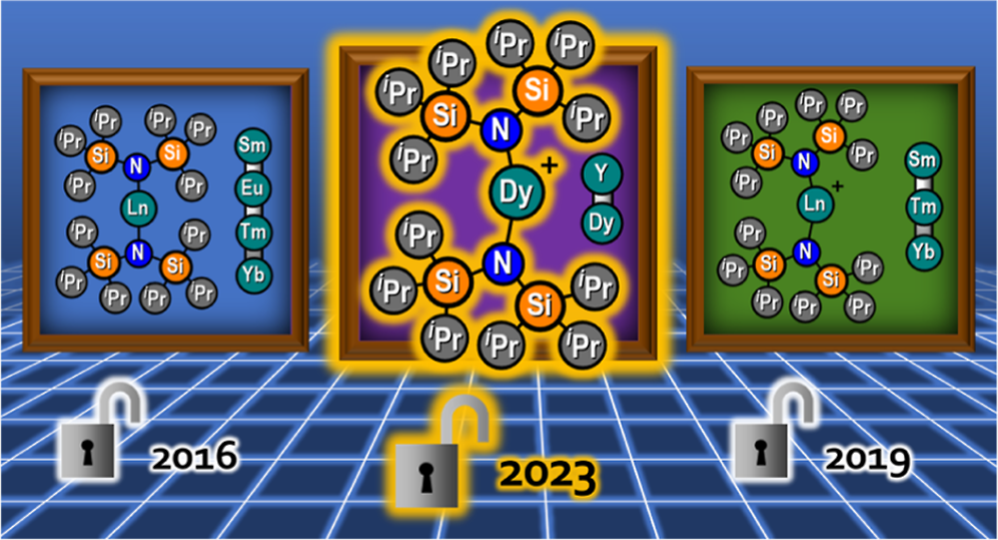

The isolation of formally two-coordinate lanthanide (Ln) complexes is synthetically challenging, due to predominantly ionic Ln bonding regimes favoring high coordination numbers. In 2015, it was predicted that a near-linear dysprosium bis(amide) cation [Dy{N(Si^i^Pr_3_)_2_}_2_]^+^ could provide a single-molecule magnet (SMM) with an energy barrier to magnetic reversal (*U*_eff_) of up to 2600 K, a 3-fold increase of the record *U*_eff_ for a Dy SMM at the time; this work showed a potential route to SMMs that can provide high-density data storage at higher temperatures. However, synthetic routes to a Dy complex containing only two monodentate ligands have not previously been realized. Here, we report the synthesis of the target bent dysprosium bis(amide) complex, [Dy{N(Si^i^Pr_3_)_2_}_2_][Al{OC(CF_3_)_3_}_4_] (**1-Dy**), together with the diamagnetic yttrium analogue. We find *U*_eff_ = 950 ± 30 K for **1-Dy**, which is much lower than the predicted values for idealized linear two-coordinate Dy(III) cations. Ab initio calculations of the static electronic structure disagree with the experimentally determined height of the *U*_eff_ barrier, thus magnetic relaxation is faster than expected based on magnetic anisotropy alone. We propose that this is due to enhanced spin–phonon coupling arising from the flexibility of the Dy coordination sphere, in accord with ligand vibrations being of equal importance to magnetic anisotropy in the design of high-temperature SMMs.

## Introduction

Raising the temperatures at which single-molecule magnets (SMMs) exhibit magnetic remanence is key to unlocking their potential applications in high-density data storage, as the liquid helium cooling currently required is expensive and unsustainable for widespread adoption.^1−3^ For the last two decades, lanthanide (Ln) SMMs have shown the most promise to achieve this goal,^4−7^ with axial dysprosium (Dy) complexes predicted to show the highest energy barriers to magnetic reversal (*U*_eff_) as these ligand fields best complement the magnetic anisotropy intrinsic to the Dy(III) ion.^8−10^ As the isolation of an ideal axial two-coordinate linear Dy(III) complex is a major synthetic challenge (see below), pentagonal bipyramidal Dy complexes with strongly donating apical alkoxides and five weak equatorial donor ligands were the first SMMs to achieve *U*_eff_ values > 1000 K.^11^ Salts with axial dysprosocenium cations [Dy(Cp^R^)_2_]^+^ (Cp^R^ = substituted cyclopentadienyl) and related derivatives subsequently raised 100 s magnetic blocking temperatures (*T*_B_) ever closer to the boiling point of liquid nitrogen (77 K);^12−22^ this was attributed to the rigidity of the coordinated aromatic ligands expediting magnetic relaxation via Raman pathways.^12,23^ The current record-holding SMM [Dy_2_(C_5_^i^Pr_5_)_2_(μ-I)_3_] has a *U*_eff_ of 2345 ± 36 K and a *T*_B_ of 72 K, with its 1e^–^ Dy–Dy bond providing a significant contribution to these parameters.^19^

Prior to the isolation of these high-barrier SMMs, the near-linear Ln(II) complexes [Ln{N(Si^i^Pr_3_)_2_}_2_] (Ln = Sm, Eu, Tm, and Yb) were prepared by salt metathesis reactions of 2 equiv of [K{N(Si^i^Pr_3_)_2_}] with parent LnI_2_.^24,25^ The experimentally determined atomic coordinates of the Sm(II) derivative (N–Ln–N: 175.5(2)°) were used to calculate that an analogous Dy(III) bis(amide) cation [Dy{N(Si^i^Pr_3_)_2_}_2_]^+^ could show *U*_eff_ ≈ 2600 K;^24^ this value was over triple that of the magnitude of the record barrier for Dy SMMs at the time (842 K for a polymetallic Dy-doped yttrium alkoxide complex).^26^ Further calculations revealed that *U*_eff_ values could remain >1300 K even if the N–Dy–N angle was reduced to as low as 120°, provided that no additional ligands were coordinated.^27^ In the interim, the bent Ln(III) bis(amide) complexes [Ln{N(Si^i^Pr_3_)_2_}_2_][B(C_6_F_5_)_4_] for Ln = Sm, Tm, and Yb were synthesized by oxidation of the parent Ln(II) complexes [Ln{N(Si^i^Pr_3_)_2_}_2_];^24,25,28,29^ recently, a related bent Yb(III) bis(amide) complex, [Yb{N(SiPh_2_Me)_2_}_2_][Al{OC(CF_3_)_3_}_4_], has been reported.^30^ However, due to synthetic difficulties associated with directly installing bulky silylamides at small, charge-dense Ln(III) centers by salt metathesis protocols where side-reactions may occur (see below), the desired Dy(III) bis(amide) cation [Dy{N(Si^i^Pr_3_)_2_}_2_]^+^ has not previously been isolated. Indeed, the isolation of any Dy complex containing only two monodentate ligands has proved elusive for the wider synthetic chemistry community to date, which is particularly hampered by the predominantly ionic bonding regimes of relatively large Ln cations favoring higher coordination numbers (CNs).^31−33^

Here, we report the isolation and characterization of the bent Dy bis(amide) complex [Dy{N(Si^i^Pr_3_)_2_}_2_][Al{OC(CF_3_)_3_}_4_] (**1-Dy**), together with the diamagnetic yttrium analogue **1-Y**, and other complexes that were prepared as starting materials toward these synthetic targets. Magnetic measurements reveal that the SMM properties of **1-Dy** are not as favorable as originally predicted, with magnetization *vs* field hysteresis loops close at zero field at 2 K. Ab initio calculations show that the bent geometry still imposes very large magnetic anisotropy in **1-Dy**, as large as for the first dysprosocenium cation [Dy(Cp^ttt^)_2_]^+^.^12,15,16^ As molecular rigidity has been shown to be crucial for controlling spin–phonon relaxation in dysprosocenium cations,^12^ we propose that the flexible coordination environment in **1-Dy** enables rapid magnetic relaxation; the large magnetic anisotropy generated by crystal-field (CF) splitting will therefore not necessarily result in a high-barrier Ln SMM unless spin–phonon relaxation enabled by molecular vibrations is adequately controlled.

## Results

### Synthesis

**Scheme 1:**
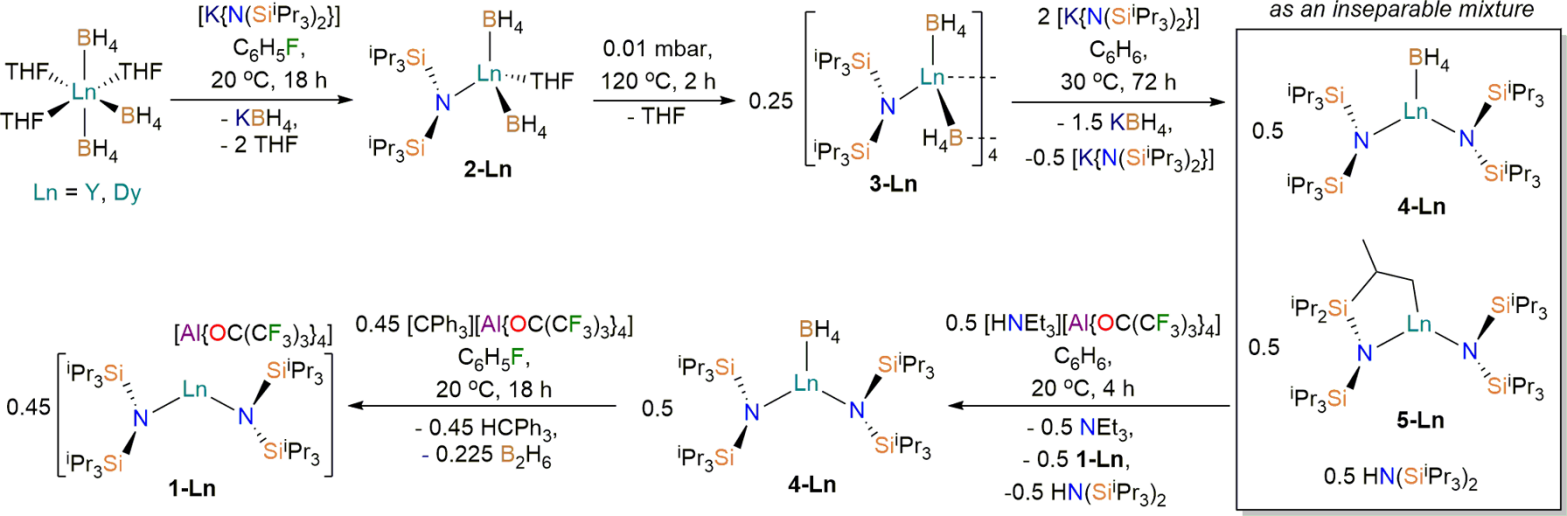
Synthesis of **1-Ln**, **2-Ln**, **3-Ln**, **4-Ln**, and **5-Ln** (Ln = Y and Dy)

Complexes **1-Ln** were prepared by the synthetic route shown in Scheme 1; we note that either fluorobenzene or benzene is used as a reaction solvent and in most steps is interchangeable; see the Experimental Section for full details. Analysis of mass balances and in situ ^1^H NMR spectra for the Y congeners indicated that all reactions proceeded with complete consumption of the starting materials to give the products indicated exclusively. The separate salt elimination reactions of [Ln(BH_4_)_3_(THF)_3_] (Ln = Y and Dy)^34^ with 1 equiv of [K{N(Si^i^Pr_3_)_2_}]^24^ in fluorobenzene at ambient temperature for 1 h gave the heteroleptic Ln(III) mono(amide) bis(borohydride) complexes [Ln{N(Si^i^Pr_3_)_2_}(BH_4_)_2_(THF)] (**2-Ln**) in 79–83% isolated yields following filtration and crystallization from *n*-hexane at −35 °C. The bound THF was removed from **2-Ln** by heating solid samples at 120 °C for 2 h at 0.01 mbar. Recrystallization of the desolvated products from 1,2-difluorobenzene layered with *n*-hexane gave, by slow diffusion, crystals of tetranuclear Ln(III) complexes [Ln{N(Si^i^Pr_3_)_2_}(BH_4_)(μ-BH_4_)]_4_ (**3-Ln**) in 54–67% isolated yields. It is critical to remove all of the KBH_4_ evolved during the synthesis of **2-Ln**; failure to do so has a deleterious impact on the thermal desolvation step. We also found that samples of **3-Ln** recrystallized in the presence of trace amounts of KBH_4_ were contaminated with several crystals of the adducts [{Ln{N(Si^i^Pr_3_)_2_}(BH_4_)(μ-BH_4_)}_2_{K(μ-BH_4_)}]_∞_ (**3-Ln·0.5KBH**_**4**_), for which we report the single-crystal XRD structures here for completeness. The optimized experimental procedures described herein provide pure samples of **2-Ln**.

The separate salt elimination reactions of **3-Ln** with an excess (2 equiv) of [K{N(Si^i^Pr_3_)_2_}] in benzene at the optimal temperature of 30 °C for 72 h gave full conversion to a mixture of the desired Ln(III) bis(amide) borohydride complexes [Ln{N(Si^i^Pr_3_)_2_}_2_(BH_4_)] (**4-Ln**), species assigned as Ln(III) cyclometalates [Ln{N(Si^i^Pr_3_)_2_}{N(Si^i^Pr_3_)[Si(^i^Pr)_2_{CH(Me)CH_2_}]-κ^2^-*N*,*C*}] (**5-Ln**) and HN(Si^i^Pr_3_)_2_; this product distribution is in accord with deprotonation of a silyl group in situ, likely promoted by a highly Lewis acidic Dy(III) ion.^31^ The physical separation of these three highly alkane-soluble species proved challenging; as such, the mixture of **4-Ln**, **5-Ln**, and HN(Si^i^Pr_3_)_2_ was treated with 0.5 equiv of [HNEt_3_][Al{OC(CF_3_)_3_}_4_]^28^ in benzene at 20 °C for 4 h, in order to protonate **5-Ln** and generate the target homoleptic Ln(III) bis(amide) complexes **1-Ln**. After removal of benzene and NEt_3_ under vacuum, the resultant oils were triturated with *n*-hexane to give a solution containing a mixture of **4-Ln** and HN(Si^i^Pr_3_)_2_, together with the solid **1-Ln** and the contaminant [HNEt_3_][Al{OC(CF_3_)_3_}_4_]. The suspensions were filtered, the volatiles of the filtrates were removed in vacuo, and the resultant oils were recrystallized from hexamethyldisiloxane at −30 °C to enable the separation of **4-Ln** from HN(Si^i^Pr_3_)_2_. Unfortunately, we found the solid mixtures of **1-Ln** and [HNEt_3_][Al{OC(CF_3_)_3_}_4_] challenging to separate; recrystallization from fluorobenzene solutions layered with *n*-hexane consistently afforded the cocrystallized material of both ionic species. As such, pure samples of **1-Ln** were instead prepared in 73–79% crystalline yields by a hydride abstraction strategy via the reaction of isolated **4-Ln** with 0.9 equiv of [CPh_3_][Al{OC(CF_3_)_3_}_4_]^35^ in fluorobenzene at 20 °C for 18 h, followed by slow diffusion of *n*-hexane into the resulting solutions. Treating a mixture of **1-Y** and [HNEt_3_][Al{OC(CF_3_)_3_}_4_] with excess [K{N(Si^i^Pr_3_)_2_}] gave ^1^H, ^13^C{^1^H} DEPTQ, ^29^Si{^1^H} DEPT90, and ^19^F NMR spectra that are consistent with the concomitant formation of **5-Y**, HN(Si^i^Pr_3_)_2_, and K[Al{OC(CF_3_)_3_}_4_]. We have not yet been able to isolate **5-Ln** in pure form for further characterization, but NMR data for a C_6_D_6_ solution of a 1:2 mixture of **5-Y** and HN(Si^i^Pr_3_)_2_ are in line with the proposed cyclometalate formulation (see below).

### Spectroscopic Characterization

Bulk samples of crystalline **1-Ln**, **2-Ln**, **3-Ln**, and **4-Ln** were characterized by elemental analysis, multinuclear NMR spectroscopy, and ATR-IR spectroscopy (see Supporting Information Figures S1–S48 for annotated NMR spectra of **1-Ln**, **2-Ln**, **3-Ln**, **4-Ln**, and **5-Y**). The ^1^H and ^13^C{^1^H} DEPTQ NMR spectra of **1-Y** in C_6_H_5_F solution show the anticipated resonances for the methyl and methine environments of the ^i^Pr groups, with no additional Y–C or Y–H coupling observed (^89^Y, *I* = 1/2, 100% abundance). The ^29^Si{^1^H} DEPT90 NMR spectrum of **1-Y** shows a single resonance at δ_Si_ = −4.8 ppm, while no resonance was observed in the corresponding spectrum of **1-Dy**. The ^19^F NMR spectrum of **1-Dy** contains a single broad resonance at δ_F_ = −97.2 ppm (full-width at half-maximum, fwhm ≈ 280 Hz) for the [Al{OC(CF_3_)_3_}_4_]^−^ anion, that is paramagnetically shifted relative to that seen for **1-Y** (δ_F_ = −75.1 ppm).

The ^1^H, ^11^B, ^13^C{^1^H} DEPTQ, and ^29^Si{^1^H} DEPT90 NMR spectra of diamagnetic **2-Y**, **3-Y**, and **4-Y** were fully assigned in C_6_D_6_ solution. For brevity, we do not provide a full discussion of all resonances in the ^1^H and ^13^C{^1^H} DEPTQ NMR spectra here as chemical shifts and coupling constants were in line with expected values for the functional groups present. In C_6_D_6_, the ^11^B NMR spectra of **2-Y** and **4-Y** feature a single pentet resonance at δ_B_ = −22.0 ppm (^1^*J*_BH_ = 81 Hz) and δ_B_ = −21.4 ppm (^1^*J*_BH_ = 86 Hz), respectively. By contrast, for **3-Y**, two broad resonances were observed at δ_B_ = −20.7 and −3.6 ppm, which we assign as the terminal and bridging BH_4_ groups, respectively, implying that this species remains oligomeric in benzene solution. In 1,2-difluorobenzene, a broad pentet resonance was observed at δ_B_ = −20.9 ppm (^1^*J*_BH_ = 80 Hz) for **3-Y**, consistent with a fluxional monomeric species, possessing equivalent time-averaged terminal BH_4_ groups. The ^29^Si{^1^H} DEPT90 NMR spectra of **2-Y**, **3-Y**, and **4-Y** in C_6_D_6_ contain a similarly shielded singlet resonance (δ_Si_/ppm = −2.7, **2-Y**; −3.7, **3-Y**; −3.4, **4-Y**); with **3-Y**, no significant difference was observed in 1,2-difluorobenzene (δ_Si_ = −3.6 ppm). Interpretation of the NMR data for **2-Dy**, **3-Dy**, and **4-Dy** was limited due to paramagnetic broadening of signals, though their ^11^B{^1^H} NMR spectra contain a single resonance (δ_B_/ppm = −18.1, **2-Dy**; −9.9, **3-Dy**; −14.1, **4-Dy**). Additionally, the solution magnetic susceptibilities of these complexes were determined at 298 K by the Evans method;^36^ all values obtained (range χ*T* = 12.5–14.2 cm^3^ K mol^–1^) are in line with that expected for a Dy(III) free ion (χ*T* = 14.17 cm^3^ K mol^–1^).^37^

The species assigned as the cyclometalate **5-Y** exhibits three signals in the ^29^Si{^1^H} DEPT90 NMR spectrum at −3.2, −5.8, and −7.6 ppm. Three magnetically inequivalent silyl groups are also seen in the corresponding ^1^H and ^13^C{^1^H} DEPTQ NMR spectra; the latter spectrum contains a doublet resonance at δ_C_ = 55.3 ppm, ^1^*J*_YC_ = 47.4 Hz with the correct phase for a methylene group, which was assigned to the Y-bound carbon atom. Due to the presence of multiple coincident resonances in ^1^H–^13^C HSQC and ^1^H–^13^C HMBC NMR spectra, we were unable to assign the associated ^1^H resonance. The resonances assigned to **5-Y** in the ^1^H, ^13^C{^1^H} DEPTQ, and ^29^Si{^1^H} DEPT90 NMR spectra of **5-Y** are comparable to those previously reported for the Y(III) silylamide cyclometalate [Y{N(SiMe_3_)_2_}_2_{N(SiMe_3_)[Si(Me)_2_CH_2_]-κ^2^-*N*,*C*}K], which has δ_Si_ = −26.1, −13.5, and −12.0 ppm, and also shows resonances for the bound methylene group at δ_H_ = −1.27 ppm (^2^*J*_YH_ = 2.6 Hz) and δ_C_ = 23.6 ppm (^1^*J*_YC_ = 22.9 Hz).^38^

The ATR-IR spectra of each Dy/Y pair in **1-Ln**, **2-Ln**, **3-Ln**, and **4-Ln** overlap with each other and show a number of red-shifted C–H stretching bands that are diagnostic of some methine and methyl groups being in close proximity to and interacting with the Ln centers (see Supporting Information Figures S49–S56). These spectroscopic markers were corroborated by a qualitative analysis of the density-functional theory (DFT)-calculated IR spectra for **1-Y**, **2-Y**, **3-Y**, and **4-Y** (see Supporting Information Figures S57–S60) and are in accord with their crystallographically determined solid-state structures (see below and Supporting Information Figures S61–S70). For **1-Ln**, these features extended down to 2560 cm^–1^, with the lowest energy modes computationally assigned to the methine C–H group that is closest to the Ln center; the corresponding resonance for **3-Ln** was observed at 2745 cm^–1^, and for **4-Ln** there are two bands at 2756 and 2729 cm^–1^. Characteristic borohydride vibrations were also observed for **2-Ln**, **3-Ln**, and **4-Ln**.^39^ The apical B–H stretch of the terminal borohydrides corresponded to sharp features at 2486, 2519, and 2495 cm^–1^ for **2-Ln**, **3-Ln**, and **4-Ln**, respectively, while the stretching modes of the B–H bonds that are proximal to the metal gave broad, convoluted bands between 2360 and 2060 cm^–1^ for all complexes.

### Solid-State Structural Characterization

**Figure 1:**
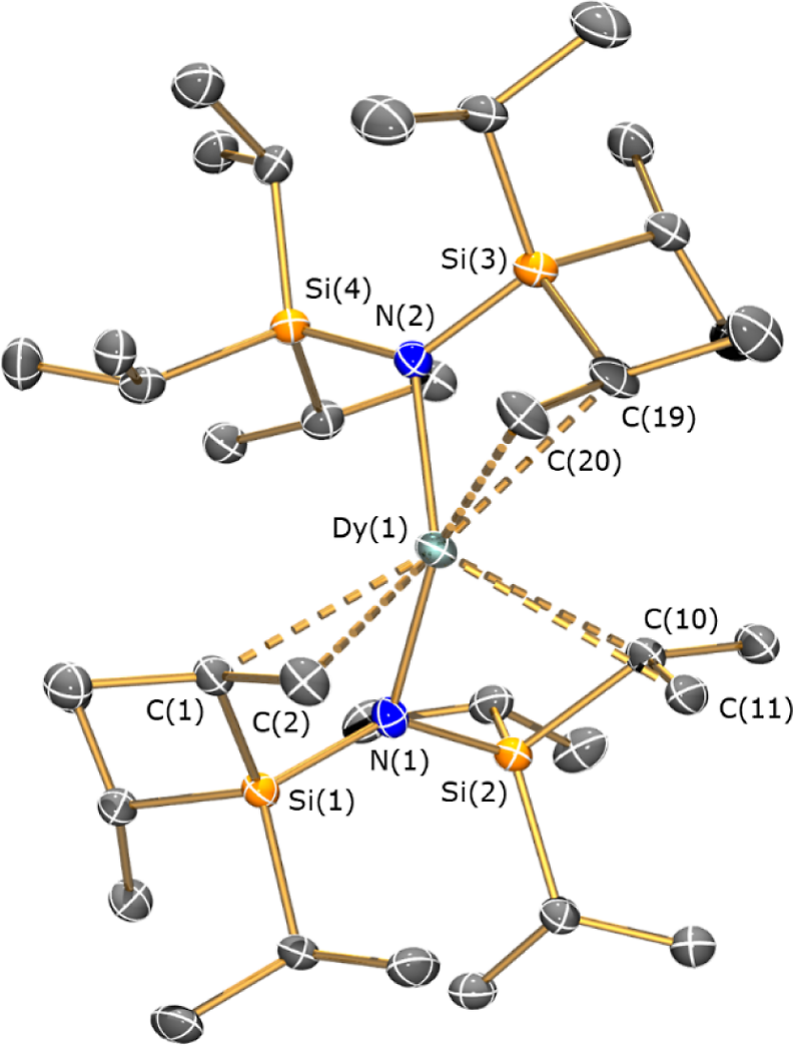
Solid-state structure of the cation of **1-Dy** at 100(2) K. Displacement ellipsoids are set at 50% probability level. Hydrogen atoms and the [Al{OC(CF_3_)_3_}_4_]^−^ counteranion are omitted for clarity. Selected bond distances (Å) and angles (deg): Dy(1)–N(1): 2.209(5); Dy(1)–N(2): 2.202(5); Dy(1)···C(1): 2.845(5); Dy(1)···C(2): 2.987(6); Dy(1)···C(10): 2.883(7); Dy(1)···C(11): 3.036(7); Dy(1)···C(19): 2.863(6); Dy(1)···C(20): 2.929(6); Dy(1)···Si(1): 3.215(2); Dy(1)···Si(2): 3.229(2); Dy(1)···Si(3): 3.207(2); N(1)–Dy(1)–N(2): 128.7(2).

The solid-state structures of **1-Ln**, **2-Ln**, **3-Ln**, **4-Ln**, and **3-Ln·0.5KBH**_**4**_ were characterized by single-crystal X-ray diffraction (XRD). All bond distances and angles are in line with expected values, and these only vary to a small extent for each Dy/Y pair in accord with the difference in six-coordinate ionic radii of Dy(III) (0.912 Å) and Y(III) (0.900 Å),^40^ thus we focus our discussion herein on the target axial Dy(III) bis(amide) cation in **1-Dy** (Figure 1). We note that the diffraction data for **1-Dy** are weak (maximum diffraction angle, 55°); see the Experimental Section for further details. The remaining structures and all crystallographic parameters are collated in the Supporting Information (see Figures S61–S71 and Tables S1–S3).

The [Dy{N(Si^i^Pr_3_)_2_}_2_]^+^ cation exhibits a bent geometry, with a N–Dy–N angle that deviates significantly from linearity (128.7(2)°), with the NSi_2_ fragments in a staggered conformation (twist angle: 63.12(6)°) and at a mean Dy–N distance of 2.206(7) Å. The structure of this cation is similar to the previously reported Sm, Tm, and Yb congeners,^28^ with deviations in metrical parameters expected on the basis of variation of Ln(III) cation size and Lewis acidity.^31^ In common with the previously reported heavy [Ln{N(Si^i^Pr_3_)_2_}_2_]^+^ cations, the Dy coordination sphere of **1-Dy** is completed by three short Dy···Si (range: 3.207(2)–3.229(2) Å), six short Dy···C (range: 2.845(5)–3.036(7) Å), and six short Dy···H distances (range: 2.293–2.455 Å) for methine and methyl fragments of three different ^i^Pr groups. These interactions are presumably driven by the electrostatic stabilization of the coordinatively unsaturated Dy(III) center by the electron density of the Si–C/C–H bonds of the silyl groups, which are proposed to set the bent geometry of the cation, as previously described for Sm, Tm, and Yb congeners.^28^ The Dy atom is sterically protected by this extremely bulky ligand system, with all visible access channels restricted to <3.4% of the solid angle at Dy (Figure S71).^41^ Powder XRD was performed on a sample of microcrystalline **1-Dy** (see Supporting Information Figure S72 and Table S4), confirming that the single-crystal structure obtained is representative of the bulk crystalline material used for magnetic characterization.

### Magnetic Measurements

**Figure 2:**
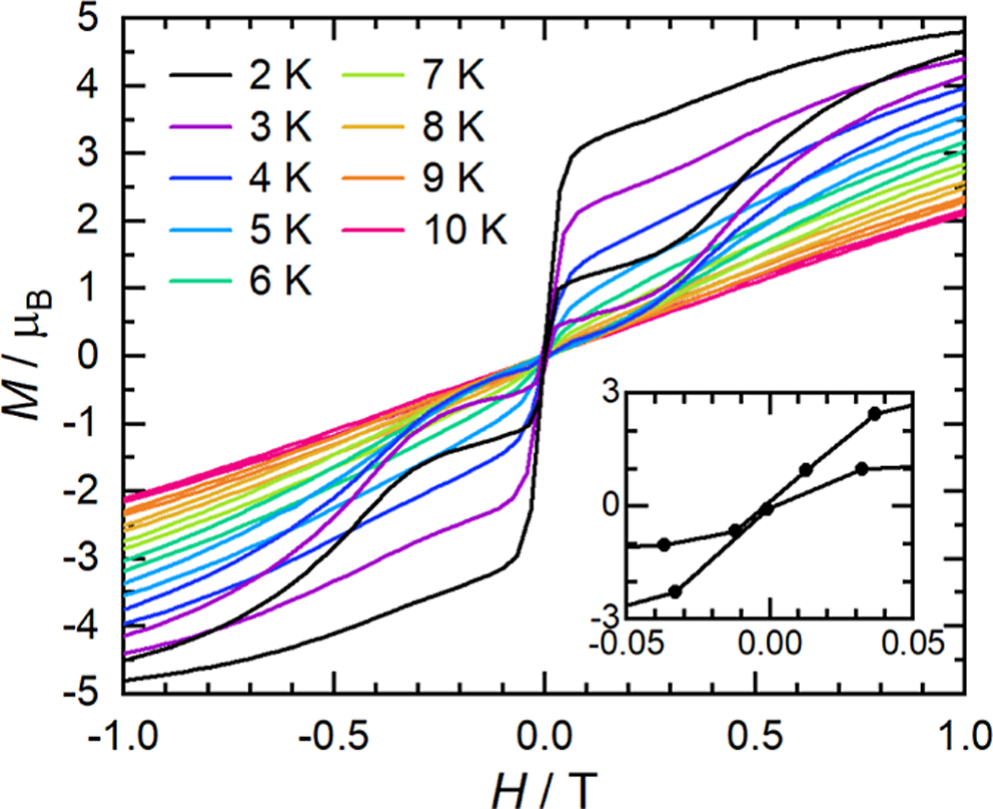
*MvsH* hysteresis loops of **1-Dy** suspended in eicosane from 2 to 10 K in between −1 and +1 T; the inset shows closing of the loop at zero field at 2 K. Sweep rate is 22 Oe s^–1^.

The static and dynamic magnetic properties of **1-Dy** in the solid state and as a 200 mM frozen solution sample in fluorobenzene were probed by dc (direct current) and ac (alternating current) susceptibility measurements (see Supporting Information Figures S73–S107 and Tables S5–S10). The *χT* value determined at 300 K under a 0.1 T dc field (14.75 cm^3^ K mol^–1^, Figure S73) is slightly higher than that determined at 298 K in fluorobenzene solution (12.97 cm^3^ K mol^–1^, Figure S74) and the expected Dy(III) free ion value (14.17 cm^3^ K mol^–1^).^37^ We observe a regular decrease in *χT* with decreasing temperature as excited CF states are thermally depopulated until *ca*. 10 K where there is a sharper decrease, reaching χ*T* = 7.71 cm^3^ K mol^–1^ at 2 K (Figure S73); zero field-cooled (ZFC) and field-cooled (FC) data collected in a smaller 0.001 or 0.005 T dc field show that this drop is mainly due to Zeeman depopulation effects, dropping only to χ*T* = 11.3 cm^3^ K mol^–1^ at 1.85 K (Figures S76 and S77). Magnetization (*M*) *vs* field (*H*) experiments show that the magnetization saturates at *M*_sat_ = 5.42 μ_B_ under a 7 T applied dc field (Figure S78), suggesting an *m*_*J*_ = ± 15/2 ground state (*M*_sat_ = 5.00 μ_B_).^7^ The waist-restricted *M vs*. *H* hysteresis loops of **1-Dy** (Figure 2) are typical for Ln SMMs;^5^ the presence of rapid quantum tunneling of magnetization (QTM) is evidenced by the closed loop at zero field at 2 K (Figure S80). The nonlinearity of the *M vs*. *H* data at low fields is in accord with rapid QTM being quenched in increasing fields.

Magnetization

**Figure 3:**
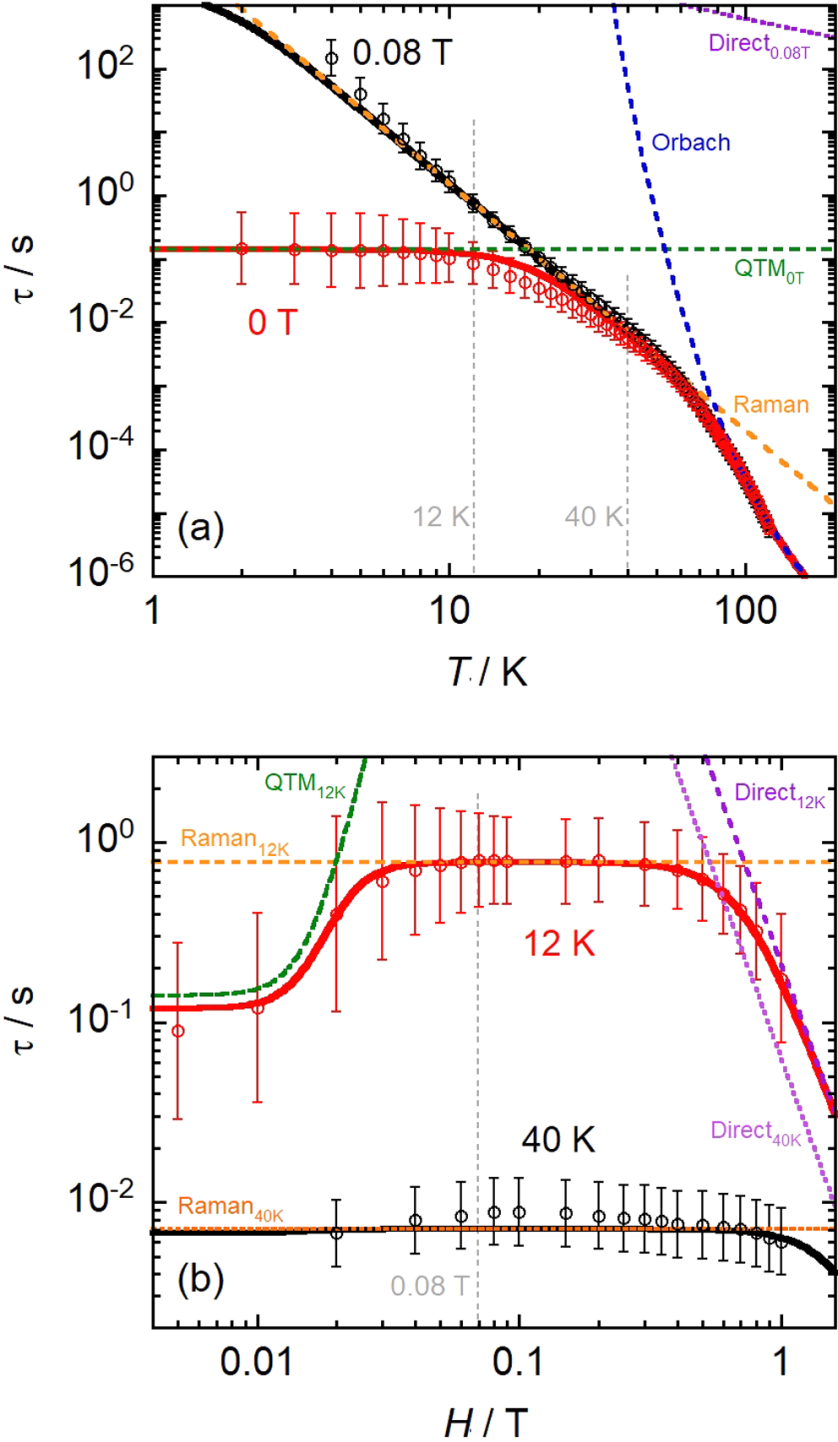
Temperature dependence (a; at 0 and 0.08 T) and field-dependence (b; at 12 and 40 K) of the magnetic relaxation time (τ) for **1-Dy**. Open circles are the experimental data and bars denote ESDs from the generalized Debye model and the relaxation time distribution.^42,43^ Solid lines are the result of the best global simulation (eq 3) as discussed in the text.

Ac susceptibility measurements of polycrystalline **1-Dy** were performed up to 10 kHz to study the magnetization dynamics. Temperature- and frequency-dependent behavior were seen for the in-phase (χ′) and out-of-phase (χ″) components of ac susceptibility in zero dc field, with maxima in χ″ due to slow relaxation of the magnetization present between 2 and 108 K (Figures S89–S91, Table S5).^42^ The ac data were fit using the generalized Debye model^42,43^ to extract relaxation times along with estimated standard deviations (ESDs) as arising from a distribution in the relaxation times;^44,45^ note that *T*_B_ is not defined in zero field as τ < 100 s at all temperatures. The temperature dependence of the magnetic relaxation time suggests Orbach relaxation at high temperatures, Raman-I relaxation at intermediate temperatures, and QTM at the lowest temperatures (Figure 3a).^2^ The average temperature-dependent relaxation time was modeled using eq 1, giving initial estimates of *U*_eff_ = 850 K, τ_0_ = 6.8 × 10^–9^ s, *C* = 8 × 10^–4^ s^–1^ K^–*n*^, *n* = 3.3, and τ_*QTM*_^–1^ = 7.4 s (Figure S104). Ac susceptibility measurements in a 0.08 T dc field (Figures S92–S94, Table S6) indicate that QTM is quenched under these conditions (Figure 3a), and the average relaxation time can be fit with eq 1, with τ_*QTM*_^–1^ = 0 to give *U*_eff_ = 915 K, τ_0_ = 3.8 × 10^–9^ s, *C* = 4 × 10^–5^ s^–1^ K^–*n*^, and *n* = 4.1 (Figure S105).
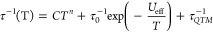
1
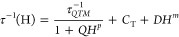
2
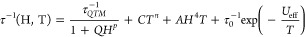
3

Performing an ac susceptibility experiment at 12 K as a function of magnetic field allows us to investigate field-dependent relaxation dynamics (Figures S95 and S96, Table S7). The relaxation time increases with increasing field below *ca*. 0.04 T and then plateaus until *ca*. 0.4 T, above which it increases again (Figure 3b). This is consistent with the quenching of QTM in low fields,^46,47^ followed by a plateau defined by the field-independent processes (predominately the Raman-I mechanism,^48^ as the Orbach contribution is insignificant at 12 K), and then an increase at higher fields owing to either a field-dependent Raman-II or a Direct single-phonon mechanism.^47,48^ Fitting the average field-dependent relaxation time with a model accounting for these three terms (eq 2) gives τ_*QTM*_^–1^ = 10 s^–1^, *Q* = 2 × 10^7^ T^–*p*^, *p* = 3.8, *C*_12 K_ = 1.3 s^–1^, *D* = 4.4 s^–1^ T^–*m*^, and *m* = 3.8 (Figure S106). The field exponent of the Raman-II/Direct term (*m*) is approaching the value expected for the single-phonon Direct process in the high-temperature limit of *m* = 4,^49^ and so we suggest this as the more likely mechanism.

The parameters of these individual models are in reasonable agreement; however, we sought to determine parameters for a unified model that can describe the complete field and temperature dependence of the relaxation time. To do so, we refined a set of global parameters for QTM, Raman-I, Direct, and Orbach processes (eq 3) against the temperature-dependent rates at 0 and 0.08 T dc fields and the field-dependent rates at 12 and 40 K (Figures S97 and S98, Table S8) using the individual model parameters as starting values (Figure 3). To reduce the number of parameters, we assumed a Direct process with *H*^4^ field dependence and linear temperature dependence,^50^ and τ_*QTM*_^–1^ was fixed to the average of the 2–5 K rates in 0 dc field. This function is complex and nonlinear, so errors were determined by independently varying each parameter such that the resultant time lies within one ESD of the experimental distributions. The global model provides good reproduction of all experimental data with the following parameters: τ_*QTM*_^–1^ = 7.1_-0.6_^+0.8^ s^–1^, *Q* = 2_–1_^+2^ × 10^10^ T^–*p*^, *p* = 5.7_–0.2_^+0.5^, *C* = 8_–1_^+2^ × 10^–5^ s^–1^ K^–*n*^, *n* = 3.9 ± 0.1, *A* = 0.4 ± 0.2 s^–1^ K^–1^ T^–4^ s^–1^, *U*_eff_ = 950 ± 30 K, and τ_0_ = (2.6 ± 0.4) × 10^–9^ s. The zero-field QTM rate (τ_*QTM*_^–1^) is rapid, in agreement with the *MvsH* hysteresis data, and the field-exponent (*p*) is slightly larger than the axial SMM [Dy(O^t^Bu)(Cl)(THF)_5_][B(C_6_F_5_)_4_] of 3.8 ± 0.2.^46^

It has previously been shown that the linearity of the N–Dy–N angle in bis–amide complexes should correlate with *U*_eff_.^27^ We hypothesized that in solution the N–Dy–N angle in **1-Dy** may increase, as has been observed for the Yb congener;^28^ phase-dependent geometries have previously been shown to be a feature of f-block silylamide chemistry.^54^ A *ca*. 200 mM solution of **1-Dy** in fluorobenzene was prepared by dissolving a known mass of solid in an appropriate mass of solvent, and this solution was flash-frozen in liquid nitrogen. The ac susceptibility experiments for the frozen solution sample give relatively noisy data (Figure S99); the Cole–Cole profiles are much broader and more asymmetric than the solid-state data and cannot be modeled well by the generalized Debye model. To account for the asymmetric distribution in relaxation times, the low-temperature (2–13 K) data were fit to a phenomenological Havriliak–Negami model (eq 4, Figures S100 and S101, Table S9) which includes parameters for the skew (γ) and the width (α) of the relaxation time distribution. For symmetric distributions (γ = 1), the Havriliak–Negami model becomes equivalent to the generalized Debye model.^51^
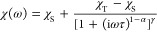
4

At higher temperatures (31–79 K), a shoulder in the Cole–Cole plot emerges, and the ac data are best fit by a double-generalized Debye model (Figures S102 and S103 and Table S10). Furthermore, magnetic hysteresis loops are more open in frozen solution than in the solid state (but remain waist-restricted, Figures S86–S88). The low-temperature frozen solution relaxation data show a comparable QTM rate to the solid-state sample, whereas the high-temperature fits encompass a major component (65%) that has rates in line with the solid-state data and a minor component (35%) that has considerably slower dynamics (Figure S107); the minor component and the hysteresis data are both in accord with a sample that relaxes measurably slower than the solid-state material. These observations are consistent with the frozen solution sample containing a broader and more asymmetric distribution of molecular geometries, with some molecules in the distribution having larger N–Dy–N angles and hence larger anisotropy and slower magnetic relaxation rates; we note that the interactions of Si–C/C–H bonds of the silyl groups with the Dy(III) ion seen in the solid state are also likely to be important in the solution phase.

### Ab Initio Calculations

First-principles complete active space self-consistent field spin–orbit (CASSCF-SO) calculations were performed using OpenMolcas^52^ from the atomic coordinates of the cation in **1-Dy** determined by single-crystal XRD. These calculations show that the ground state is an almost pure *m*_*J*_ = ±15/2 Kramers doublet, with Ising-like *g*-values (*g*_*x*_ = *g*_*y*_ = 0, *g*_*z*_ = 19.86; Table S11). The first two excited Kramers doublets are at 616 K (98% *m*_*J*_ ± 13/2, 0.8° between excited *g*_*z*_ and ground *g*_*z*_) and 1185 K (94% *m*_*J*_ ± 11/2, 1.6°) above the ground state (Figures S108 and S109), with the second excited state being highly mixed.^12^

## Discussion

All previously reported examples of axial Dy SMMs with no equatorial donor ligands contain bulky η^5^-cyclopentadienyl ligands or related derivatives,^12−22^ hence there is no literature precedent for refined magnetostructural comparisons with **1-Dy**. The Dy(III) ion in **1-Dy** is bound by two monodentate σ-donor bis(silyl)amides; although some of the charge density is delocalized about the ligand scaffolds due to negative hyperconjugation with the triisopropylsilyl groups,^53,54^ the N atoms can be formally treated as point-charge Lewis bases to a first approximation. This differs from the highest-performing Dy SMMs bound by π-aromatic ligands in the literature,^12−22^ which donate the electron density to Dy from delocalized molecular orbitals located about a pentagonal arrangement of atoms. Accordingly, the degree of magnetic anisotropy and the purity of *m*_J_ states should be more sensitive to deviations from ideal linearity in complexes like **1-Dy** than for a sandwich-type complex. We previously reported the solid-state structures of the bent Tm(III) complex [Tm{N(Si^i^Pr_3_)_2_}_2_][B(C_6_F_5_)_4_] (N–Tm–N: 125.49(9)°),^28^ and considering that the six-coordinate ionic radii of Dy(III) (0.912 Å) and Tm(III) (0.88 Å) are quite similar,^40^ we anticipated a similar N–Dy–N angle for **1-Dy**. However, previous computational studies on a series of model two-coordinate DyL_2_ compounds (L = mono- or dianionic monodentate C- or N-donor ligand) predicted that *U*_eff_ should decrease regularly with bending (e.g., [Dy{N(SiH_3_)_2_}_2_]; *U*_eff_ = 2072 cm^–1^ at 180° N–Dy–N and 919 cm^–1^ at 120°),^27^ thus **1-Dy** remained a desirable synthetic target.

The N–Dy–N angle in **1-Dy** (128.7(2)°) is far more bent than the corresponding Cp_centroid_···Dy···Cp_centroid_ angle of any isolated dysprosocenium cation (smallest known, [(C_5_^i^Pr_4_H)_2_]^+^ = 147.2(8)°),^13^ and **1-Dy** has shorter Dy–N bonds (2.206(7) Å mean) than the mean Dy···Cp_centroid_ distances in these systems (e.g., [Dy(C_5_^i^Pr_4_H)_2_]^+^ = 2.29(1) Å).^13^ Despite substantially different structures, a comparison of the total calculated CF splitting of the ^6^H_15/2_ multiplet between **1-Dy** and [Dy(Cp^ttt^)_2_]^+^ (for which we have commensurate CASSCF-SO calculations; Cp^ttt^ = {C_5_H_2_^*t*^Bu_3_-1,2,4}, mean Dy···Cp_centroid_ = 2.416(2) Å and mean Cp_centroid_···Dy···Cp_centroid_ = 152.56(7)°)^12^ shows that **1-Dy** has a substantially larger overall splitting (2459 K *vs* 2124 K); however, the energy gap to the first excited state is slightly smaller (616 *vs* 703 K), and the second excited state of **1-Dy** has large transverse *g*-values. Although these calculations may not fully capture the full effects of the multiple Dy···Si–C/C–H interactions present in **1-Dy**, we note that the two close equatorial Dy···H–C contacts seen in the solid-state structure of [Dy(Cp^ttt^)_2_][B(C_6_F_5_)_4_] did not appear to have a significant effect on SMM behavior.^12^ However, the *U*_eff_ for **1-Dy** (950 ± 30 K) is roughly half the value observed for [Dy(Cp^ttt^)_2_]^+^ (1780 ± 40 K).^44^ Conventional usage of the average transition matrix elements of magnetic moment to infer magnetic relaxation probabilities^27^ suggests that the *U*_eff_ value for **1-Dy** should be near the top of the CF manifold (Figure S108), while the experimentally determined *U*_eff_ value lies in between the first and second excited states. This discrepancy highlights that the static electronic structure alone is insufficient to predict relaxation dynamics. While this would be an excellent opportunity to deploy our recent methods to calculate magnetic relaxation dynamics ab initio,^55,56^ unfortunately, for **1-Dy**, there are eight formula units in the crystallographic unit cell (1472 atoms), thus this system is currently too large to perform periodic DFT calculations. We propose that the modulation of the CF by phonons (i.e., spin–phonon coupling) has a larger impact on the electronic states in **1-Dy** than for [Dy(Cp^R^)_2_]^+^ because in the former complex the CF is almost exclusively dominated by two flexible monatomic donor ligands rather than the more rigid η^5^-Cp^R^ rings in [Dy(Cp^R^)_2_]^+^ and related derivatives,^12−22^ which have been shown to be crucial for dictating spin dynamics in [Dy(Cp^ttt^)_2_]^+^.^57^

## Conclusions

In this work, the isolation of compounds containing the [Dy{N(Si^i^Pr)_3_}_2_]^+^ cation has realized a long-standing goal to synthesize a formally two-coordinate Dy(III) complex, allowing the magnetic properties of this new class of SMM to be determined. We have found a larger than predicted effect of the molecular geometry on SMM behavior, with the significantly bent [Dy{N(Si^i^Pr)_3_}_2_]^+^ cation showing relatively low-lying and highly mixed excited *m*_*J*_ states. Frozen solution magnetic data indicate a species with substantially slower relaxation dynamics, suggesting that a more linear N–Dy–N angle can be adopted in this phase, but this could not be unambiguously confirmed. We propose that fast magnetic relaxation in [Dy{N(Si^i^Pr)_3_}_2_]^+^ arises from a combination of its large deviation from linearity and the flexible coordination environment providing multiple close Dy···H–C/C–Si contacts, in accord with the rigidity of coordinated ligands being of equal importance to the control of molecular geometry for SMMs to show high-blocking temperatures.

## Experimental Section

### Experimental Materials and Methods

All manipulations were conducted under argon with the strict exclusion of oxygen and water by using Schlenk line and glovebox techniques. Glassware was flame-dried under vacuum prior to use. Argon was passed through a column of activated 3 Å molecular sieves and Cu catalyst prior to use. Isolated compounds were dried in vacuo on a Schlenk line to the point at which the flask could maintain a constant static pressure of less than 5 × 10^–3^ mbar. C_6_H_6_ and C_6_D_6_ were purchased in anhydrous form, degassed, and stored under argon over a K mirror or activated 3 Å molecular sieves, respectively. *n*-Hexane was refluxed over molten K for 3 days, distilled, and stored under argon over a K mirror. C_6_H_5_F and 1,2-C_6_H_5_F_2_ were stirred over neutral alumina for 4–6 h, filtered, refluxed over CaH_2_ for 3 days, distilled, and stored under argon over activated 3 Å molecular sieves. Hexamethyldisiloxane (HMDSO) was refluxed over CaH_2_ for 3 days, distilled, and stored under argon over activated 3 Å molecular sieves. [Ln(BH_4_)_3_(THF)_3_] (Ln = Y, Dy),^34^ [HNEt_3_][Al{OC(CF_3_)_3_}_4_],^17^ and [CPh_3_][Al{OC(CF_3_)_3_}_4_]^35^ were prepared according to literature procedures; [K{N(Si^i^Pr_3_)_2_}] was prepared by an adapted literature procedure,^24^ which is detailed in the Supporting Information.

NMR spectra (see Figures S1–S48) of **1-Ln**, **2-Ln**, **3-Ln**, **4-Ln**, **5-Y**/HN(Si^i^Pr_3_)_2_, HN(Si^i^Pr_3_)_2_, and [K{N(Si^i^Pr_3_)_2_}] were recorded at 298 K on a Bruker AVIII HD 400 cryoprobe spectrometer operating at 400.07 (^1^H) 128.36 (^11^B), 100.60 (^13^C), 376.40 (^19^F), or 79.48 (^29^Si) MHz. Chemical shifts are reported in ppm and coupling constants in Hz. ^1^H and ^13^C{^1^H} DEPTQ NMR spectra recorded in C_6_D_6_ were referenced to the solvent signal.^58^ Spectra recorded in protonated solvents were locked to, and where possible referenced with, an internal sealed capillary of C_6_D_6_. The solution magnetic susceptibilities of **1-Dy**, **2-Dy**, **3-Dy**, and **4-Dy** were determined at 298 K by the Evans method;^36^^1^H NMR spectra recorded in C_6_H_5_F or 1,2-C_6_H_4_F_2_ were referenced using the highest intensity peak of the highest frequency fluoroarene multiplet (δ_H_: 6.87 or 6.85 respectively). ^11^B/^11^B{^1^H} (H_3_BO_3_/D_2_O), ^19^F (C_7_H_5_F_3_/CDCl_3_), and ^29^Si{^1^H} DEPT90 (SiMe_4_) NMR spectra were referenced to external standards. The *C*(CF_3_)_3_ carbon resonances of the [Al{OC(CF_3_)_3_}_4_]^−^ anion were not observed in the ^13^C{^1^H} NMR spectra of **1-Ln**, likely due to quadrupolar broadening by the 100% abundant *I* = 5/2 ^27^Al nuclei and coupling to multiple 100% abundant *I* = 1/2 ^19^F nuclei.

ATR-IR spectra of **1-Ln**, **2-Ln**, **3-Ln**, and **4-Ln** were recorded as microcrystalline powders using a Bruker Alpha FT-IR spectrometer with a Platinum-ATR module within a nitrogen-filled glovebox at ambient temperature (see Figures S49–S56). Elemental analysis (C, H, and N) samples were prepared in an argon-filled glovebox, and the analysis was carried out either by Mr. Martin Jennings and Mrs. Anne Davies at the Microanalytical Service, Department of Chemistry, the University of Manchester, or the Elemental Analysis Services Team, Science Centre, London Metropolitan University. Elemental analysis values obtained for **1-Ln**, **2-Ln**, **3-Ln**, and **4-Ln** typically gave carbon compositions that were lower than expected values; this phenomenon has commonly been ascribed to incomplete combustion due to carbide formation, and we note that we have previously observed low carbon values reproducible for Ln {N(Si^i^Pr_3_)_2_} complexes.^24,25,28,29^

Single-crystal XRD data were collected on either an Oxford Diffraction Agilent Supernova diffractometer equipped with a CCD area detector and a mirror-monochromated Mo Kα source (**1-Y**, **2-Dy**, **3-Dy**, **3-Dy·0.5KBH**_**4**_**·1,2-C**_**6**_**H**_**4**_**F**_**2**_, and **4-Y**), a Rigaku XtalLAB Synergy-S diffractometer equipped with a HyPix 6000HE photon counting pixel array detector with a mirror-monochromated Mo Kα X-ray source (**4-Dy**), or a Rigaku FR-X diffractometer equipped with a HyPix 6000HE photon counting pixel array detector and a mirror-monochromated X-ray source (**1-Dy**, **2-Y**, **3-Y**, and **3-Y·0.5KBH**_**4**_**·C**_**6**_**H**_**6**_) (λ = 0.71073 Å for Mo Kα or λ = 1.5418 Å for Cu Kα radiation, Figures S61–S71 and Tables S1–S3). Intensities were integrated from data recorded on 0.5° (**1-Dy**, **2-Y**, **3-Y**, and **3-Y·0.5KBH**_**4**_**·C**_**6**_**H**_**6**_), or 1° (**1-Y**, **2-Dy**, **3-Dy**, **3-Dy·0.5KBH**_**4**_**·1,2-C**_**6**_**H**_**4**_**F**_**2**_, **4-Dy**, and **4-Y**) frames by ω rotation. Cell parameters were refined from the observed positions of all strong reflections in each data set. A Gaussian grid face-indexed with a beam profile was applied for all structures.^59^ The structures were solved using SHELXT;^60^ the data sets were refined by full-matrix least-squares on all unique *F*^2^ values.^60^ Anisotropic displacement parameters were used for all non-hydrogen atoms with constrained riding hydrogen geometries, with the exception of borohydride H atoms, which were located in the difference map and refined isotropically; *U*_iso_(H) was set at 1.2 (1.5 for methyl groups) times *U*_eq_ of the parent atom. The largest features in final difference syntheses were close to heavy atoms and were of no chemical significance. CrysAlisPro^59^ was used for control and integration, and SHELX^60,61^ was employed through OLEX2^62^ for structure solution and refinement. ORTEP-3^63^ and POV-Ray^64^ were used for molecular graphics. Despite the use of a highly intense X-ray source (Rigaku FR-X rotating anode), crystals of **1-Dy** and **3-Dy** only diffracted to 0.94 and 1.06 Å of resolution, respectively, and thus the data were trimmed accordingly. Complex **3-Dy** was also found to be highly sensitive to X-ray irradiation, presenting signs of beam damage (reduction of data resolution and diffracted intensities) with moderate X-ray exposure at 100 K. The data affected by the beam damage were removed, leading to a low completeness (91%) at 1.06 Å.

Powder XRD data of a microcrystalline sample of **1-Dy** mounted with a minimum amount of Fomblin were collected at 100(2) K using a Rigaku FR-X rotating-anode single-crystal X-ray diffractometer using Cu Kα radiation (λ = 1.5418 Å) with a HyPix-6000HE detector and an Oxford Cryosystems nitrogen flow gas system (Figure S72). Data were collected at θ between 2 and 70° with a detector distance of 150 mm and a beam divergence of 1.5 mRad using CrysAlisPro.^59^ For data processing, the instrument was calibrated using silver behenate as standard, then the data were reduced and integrated using CrysAlisPro.^59^ Le Bail profile analysis was performed using JANA2006 software.^65^

Magnetic measurements of **1-Dy** (Figures S73–S107 and Tables S5–S10) were performed on a Quantum Design MPMS3 superconducting quantum interference device (SQUID) magnetometer at The University of Manchester or an MPMS XL magnetometer or a PPMS EverCool II susceptometer housed at the Centre de Recherche Paul Pascal at temperatures between 1.8 and 300 K and dc magnetic fields ranging from −7 to +7 T (MPMS3 and MPMS XL) or −9 to +9 T (PPMS EverCool II).

The MPMS3 measurements were collected on a finely ground powder sample of **1-Dy** (27.4 mg) restrained in eicosane (22.0 mg) and a 200 mM fluorobenzene (0.102 g) solution of **1-Dy** (35.7 mg); these samples were prepared in a glovebox under an atmosphere of argon loaded into borosilicate tubes, which were later flame-sealed under vacuum and loaded into a plastic straw held in place by friction between the diamagnetic tape at the top of the tube and the straw. The solution sample was flash-frozen in liquid nitrogen and rapidly cooled in zero field after loading into the instrument. Measurements were performed in dc scan mode using 40 mm scan length and 6 s scan time. Equilibrium susceptibility measurements were performed on cooling in temperature settle mode 1.8–300 K (solid) or 1.8–180 K (frozen solution) in 0.1 T dc field. Field dependence (*H*) of the magnetization (*M*) curves (2 K, 0–7 T) and *MvsH* hysteresis curves (±5 T for 2–7 K; ±3 T for 8–10 or 12 K) were performed in continuous sweep mode with a sweep rate of 22 Oe s^–1^. Raw magnetic data were scaled for the shape of the sample using a Quantum Design MPMS3 Geometry Correction Simulator (correction factor: 1.003 for solid and 0.817 for solution), corrected for the diamagnetic contribution of the sample holder (straw + borosilicate tube) and for the mass of eicosane using calibrated blanks or for the mass of fluorobenzene using Pascal’s constants.^66^ The magnetic susceptibility was corrected for the intrinsic diamagnetism of the sample estimated as the molecular weight (g mol^–1^) multiplied by −0.5 × 10^–6^ cm^3^ K mol^–1^. Ac magnetic data were recorded for the frozen solution of **1-Dy** at 0.1–1000 Hz between 2 and 76 K. Low-temperature (2–13 K) ac data were fit to the Havriliak–Negami model, and high-temperature (31–79 K) data were fit to the double-generalized Debye model in CC-FIT2 5.0.1.^44,45,67^

The MPMS XL and PPMS EverCool II measurements were collected on polycrystalline **1-Dy** in sealed polypropylene (PP) bags. For the PPMS VSM dc measurements, the sample was suspended in mineral oil (MPMS RSO dc: 40.2 mg **1-Dy** and 10.9 mg PP; PPMS VSM dc: 19.5 mg **1-Dy**, 18.3 mg PP, and 13.9 mg oil; MPMS ac and PPMS ACMS ac: 40.2 mg **1-Dy** and 10.9 mg PP; MPMS ac: 22.9 mg **1-Dy** and 9.46 mg PP); these samples were prepared in a glovebox under an atmosphere of argon. Data were collected as follows: (i) via an MPMS XL for dc measurements using the RSO option with fields up to 7 T and for ac measurements in the 0.001–1500 Hz range and (ii) via a PPMS EverCool II for dc measurements with the large bore VSM option with fields up to 9 T and for the ac measurements with the ACMS-II option in the 10–10,000 Hz range. The FC/ZFC measurements were performed from 50 K cooling to 1.85 K without dc field (reset magnet). The field (10 Oe) was set at 1.85 K, and ZFC measurements were recorded upon heating up to 50 K. FC cooling measurements were performed by cooling from 50 to 1.85 K, and FC heating data were collected upon heating from 1.85 to 50 K. During the MPMS XL and PPMS EverCool II experiments, it is clear that the amplitude of the magnetization for **1-Dy** decreased slightly over a period of several weeks and more rapidly during sample transfer/loading in the experimental setup; we attribute this to a small amount of sample decomposition as **1-Dy** is relatively air- and moisture-sensitive and the PP bags are not completely impervious to air and moisture. However, no modification of the global, qualitative magnetic behavior was seen, with no shift or shape modification of the relaxation process. Therefore, the amplitude of the magnetic data presented herein was normalized to the MPMS3 measurements collected at Manchester directly after the synthesis and isolation of **1-Dy** in sealed borosilicate tubes. The maximum normalization factor used in this study was 1.19, which also incorporates errors in the sample mass, magnetometer calibration, and background corrections. Ac data for solid **1-Dy** were fitted with the generalized Debye model with MagSuite software, restraining the frequency window to where the model fits well.^68^

### Computational Methods

OpenMolcas^50^ was used to perform CASSCF-SO calculations on **1-Dy** to determine its electronic structure (Figures S108 and S109 and Table S11). The molecular geometry of the single-crystal XRD structure was used with no optimization, taking the largest disorder component only. Integrals were performed in the SEWAD module using basis sets from ANO-RCC library^69−72^ with VTZP quality for Dy atoms, VDZP quality for the N atoms, and VDZ quality for all remaining atoms, employing the second-order DKH transformation. Cholesky decomposition of the two-electron integrals with a threshold of 10^–8^ was performed to save disk space and reduce computational demand. The molecular orbitals (MOs) were optimized in state-averaged CASSCF calculations in the RASSCF module, where the active space was defined by the nine 4f electrons in the seven 4f orbitals of Dy(III). Three such calculations were performed independently for each possible spin state, where 21 roots were included for *S* = 5/2, 224 roots were included for *S* = 3/2, and 490 roots were included for *S* = 1/2. The wave functions obtained from these CASSCF calculations were then mixed by spin–orbit coupling in the RASSI module, where all the 21 *S* = 5/2 states, 128 of the *S* = 3/2 states, and 130 of the *S* = 1/2 states were included. SINGLE_ANISO was used to decompose the resulting spin–orbit wave functions into the CF Hamiltonian formalism.^73^ Diamond was employed for molecular graphics.^74^

DFT geometry optimizations and vibrational analyses were performed on the cation of **1-Y** and **2-Y**, the monomer of **3-Y**, and **4-Y**, for the purposes of assigning experimental IR spectra (Figures S57–S60). All calculations were executed by the Orca 5.0 software package at the PBE0^75,76^-D4^77,78^/def2-TZVP^79^ level (including the default effective core potential for yttrium^80^). The default Orca 5.0 integration grids, convergence method, and convergence thresholds (for both SCF and geometry iterations) were used throughout. The SCF energy calculations were expedited by employing the RIJCOSX approximation^81^ (and the associated def2/J auxiliary basis set^82^) and DIIS convergence acceleration^83^ (as is default in Orca 5.0). Geometry-optimized structures were verified as being minima on the potential energy surface through the absence of imaginary vibrational modes. A linear energy scaling was applied to the computed IR spectra.

### Synthesis

#### [Y{N(Si^i^Pr_3_)_2_}_2_][Al{OC(CF_3_)_3_}_4_] (**1-Y**)

C_6_H_5_F (5 mL) was added to a mixture of **4-Y** (0.168 g, 0.221 mmol) and [CPh_3_][Al{OC(CF_3_)_3_}_4_] (0.242 g, 0.200 mmol), and the reaction mixture was stirred for 18 h at 20 °C. The resulting suspension was filtered and layered with excess *n*-hexane (*ca*. 20 mL), which upon diffusion at ambient temperature afforded the title compound as colorless needles. The crystals were isolated and dried thoroughly in vacuo. Yield: 0.251 g, 0.146 mmol, 73%. This reaction can also be performed in benzene with equivalent success, yielding under the same conditions a biphasic mixture comprising a benzene-clathrated oil of **1-Y** beneath a benzene solution of other reaction components. Anal. Calcd for C_52_H_84_AlF_36_O_4_N_2_Si_4_Y (1713.42 g mol^–1^) C, 36.45; H, 4.94, N, 1.63. Found: C, 34.38; H, 4.51, N, 1.41. ^1^H NMR (400.07 MHz, C_6_H_5_F): δ 1.15 (d, ^3^*J*_HH_ = 7.4 Hz, 72H, C*H*_3_), 0.86 (sept, ^3^*J*_HH_ = 7.4 Hz, 12H, C*H*). ^13^C{^1^H} DEPTQ NMR (100.60 MHz, C_6_H_5_F): δ 123.28 (q, ^1^*J*_FC_ = 294 Hz, CF_3_), 19.57 (s, *C*H_3_), 18.25 (s, *C*H). ^19^F NMR (376.40 MHz, C_6_H_5_F): δ −75.05 (s, C*F*_3_). ^29^Si{^1^H} DEPT90 NMR (79.48 MHz, C_6_H_5_F): δ −4.79 (s, *Si*^i^Pr_3_). FTIR (ATR, microcrystalline): ν̃ = 2945 (m), 2867 (m), 1465 (m), 1352 (m), 1296 (m), 1274 (m), 1241 (m), 1208 (s) 1167 (m), 968 (s), 953 (m), 920 (m), 880 (m), 725 (s) cm^–1^.

#### [Dy{N(Si^i^Pr_3_)_2_}_2_][Al{OC(CF_3_)_3_}_4_] (**1-Dy**)

C_6_H_5_F (5 mL) was added to a mixture of **4-Dy** (0.350 g, 0.418 mmol) and [CPh_3_][Al{OC(CF_3_)_3_}_4_] (0.484 g, 0.400 mmol), and the reaction mixture was stirred for 18 h at 20 °C. The resulting suspension was filtered and layered with excess *n*-hexane (*ca*. 20 mL), which upon diffusion at ambient temperature afforded the title compound as colorless needles. The crystals were isolated and dried thoroughly in vacuo. Yield: 0.567 g, 0.317 mmol, 79%. This reaction can also be performed in benzene with equivalent success, yielding under the same conditions a biphasic mixture comprising a benzene-clathrated oil of **1-Dy** beneath a benzene solution of the other reaction components. Anal. Calcd for C_52_H_84_AlDyF_36_O_4_N_2_Si_4_ (1786.91 g mol^–1^) C, 34.95; H, 4.74, N, 1.57. Found: C, 33.66; H, 4.37, N, 1.41. χ*T* product = 14.2 cm^3^ mol^–1^ K, μ_eff_ = 10.7 μ_B_ mol^–1^ (Evans method). ^1^H NMR (400.07 MHz, C_6_D_6_): δ 4.76 (br, fwhm ≈ 60 Hz), 4.23 (br, fwhm ≈ 50 Hz), 3.95 (br, fwhm ≈ 160 Hz), 3.67 (br, fwhm ≈ 60 Hz). ^19^F NMR (376.40 MHz, C_6_H_5_F): δ 97.22 (br, fwhm ≈ 280 Hz, C*F*_3_). FTIR (ATR, microcrystalline): ν̃ = 2945 (m), 2867 (m), 1465 (m), 1352 (m), 1296 (m), 1274 (m), 1241 (m), 1208 (s) 1167 (m), 968 (s), 953 (m), 920 (m), 880 (m), 725 (s) cm^–1^.

#### [Y{N(Si^i^Pr_3_)_2_}(BH_4_)_2_(THF)] (**2-Y**)

C_6_H_5_F (10 mL) was added to a mixture of [Y(BH_4_)_3_(THF)_3_] (2.389 g, 5.000 mmol) and [K{N(Si^i^Pr_3_)_2_}] (1.857 g, 5.050 mmol), and the resulting suspension was stirred at 20 °C for 1 h. The volatiles were removed in vacuo, and the residues were extracted into *n*-hexane (3 × 20 mL) with vigorous agitation and filtered. The solution was concentrated to *ca*. 10 mL and stored at −25 °C, affording the title complex as colorless needles, which were isolated and thoroughly dried in vacuo. Yield: 2.298 g, 4.424 mmol, 88%. This reaction can also be successfully performed in benzene, following an otherwise identical procedure. Anal. Calcd for C_22_H_58_B_2_ONSi_2_Y (519.35 g mol^–1^) C, 50.87; H, 11.26, N, 2.70. Found: C, 49.55; H, 11.09, N, 2.45. ^1^H NMR (400.07 MHz, C_6_D_6_, 298 K): δ 3.60 (br s, fwhm ≈ 19 Hz, 4H, CH_2_C*H*_2_O), 1.33 (d, ^3^*J*_HH_ = 7.3 Hz, 36H, C*H*_3_CH), 1.26 (q, 8H, ^1^*J*_BH_ = 81 Hz, B*H*_4_), 1.15 (sept, ^3^*J*_HH_ = 7.3 Hz, 6H, CH_3_C*H*), 1.06 (br s, fwhm ≈ 21 Hz, 4H, C*H*_2_CH_2_O). ^11^B NMR (128.36 MHz, C_6_D_6_, 298 K): δ 22.0 (p, ^1^*J*_BH_ = 81 Hz, *B*H_4_). ^13^C{^1^H} DEPTQ NMR (100.60 MHz, C_6_D_6_, 298 K): δ 74.6 (CH_2_*C*H_2_O), 24.8 (*C*H_2_CH_2_O), 20.6 (*C*H_3_CH), 18.4 (CH_3_*C*H). ^29^Si{^1^H} DEPT90 NMR (79.48 MHz, C_6_D_6_, 298 K): δ −2.7 (*Si*^i^Pr_3_). FTIR (ATR, microcrystalline): ν̃ = 2951 (m), 2867 (m), 2488 (m), 2178 (b), 1463 (m), 1185 (m), 1095 (w), 999 (m), 923 (s), 873 (s), 719 (s), 653 (s) cm^–1^.

#### [Dy{N(Si^i^Pr_3_)_2_}(BH_4_)_2_(THF)] (**2-Dy**)

C_6_H_5_F (10 mL) (or benzene, see above) was added to a mixture of [Dy(BH_4_)_3_(THF)_3_] (2.757 g, 5.000 mmol) and [K{N(Si^i^Pr_3_)_2_}] (1.857 g, 5.050 mmol), and the resulting suspension was stirred at 20 °C for 1 h. The volatiles were removed in vacuo, and the residues were extracted into *n*-hexane (3 × 20 mL) with vigorous agitation and filtered. The solution was concentrated to *ca*. 10 mL and stored at −25 °C, affording the title complex as pale-yellow needles, which were isolated and thoroughly dried in vacuo. Yield: 2.470 g, 4.165 mmol, 83%. This reaction can also be successfully performed in benzene, following an otherwise identical procedure. Anal. Calcd for C_22_H_58_B_2_DyONSi_2_ (592.94 g mol^–1^) C, 44.56; H, 9.86, N, 2.36. Found: C, 43.86; H, 10.22, N, 2.84. χ*T* product = 13.9 cm^3^ mol^–1^ K, μ_eff_ = 10.5 μ_B_ mol^–1^ (Evans method). ^1^H NMR (400.07 MHz, C_6_D_6_): δ 40.13 (vbr, fwhm ≈ 650 Hz, OC*H*_2_CH_2_), 1.55 (br, fwhm ≈ 20 Hz), 1.11 (br, fwhm ≈ 20 Hz, C*H*_3_), 0.78 (br, fwhm ≈ 20 Hz), 0.63 (br, fwhm ≈ 20 Hz), −42.80 (vbr, fwhm ≈ 1610 Hz, OCH_2_C*H*_2_). The B*H*_4_ resonance was not located. ^11^B{^1^H} NMR (128.36 MHz, C_6_D_6_): δ −18.14 (vbr, fwhm ≈ 2490 Hz), *B*H_4_. FTIR (ATR, microcrystalline): ν̃ = 2951 (m), 2867 (m), 2488 (m), 2178 (b), 1463 (m), 1185 (m), 1095 (w), 999 (m), 923 (s), 873 (s), 719 (s), 653 (s) cm^–1^.

#### [Y{N(Si^i^Pr_3_)_2_}(BH_4_)(μ-BH_4_)]_4_ (**3-Y**)

In a long resealable ampule, **2-Y** (4.290 g, 8.259 mmol) was heated to 120 °C in the solid state at *ca*. 0.01 mbar for 2 h, resulting in partial sublimation of the solid material. After cooling to ambient temperature, the residues were found to have a mass of 3.699 g (8.270 mmol assuming the formula weight of **3-Y**), consistent with the removal of 99.2% of the bound THF. The ^1^H, ^11^B, ^13^C, and ^29^Si NMR spectra of this amorphous material were found to be identical to an authentic crystalline sample of **3-Y**, which was prepared as follows. In a long resealable ampule, **2-Y** (1.390 g, 2.676 mmol) was heated to 150 °C in the solid state at *ca*. 0.01 mbar for 2 h, resulting in the partial sublimation of the solid material and minor production of a high-boiling point colorless oil. After cooling to ambient temperature, the residues were extracted into 1,2-C_6_H_4_F_2_ (3 × 5 mL), filtered, and the solution concentrated to *ca*. 5 mL. Slow diffusion of excess *n*-hexane (*ca*. 20 mL) at −25 °C gave the title complex as colorless plates, which were isolated and thoroughly dried in vacuo. An additional crop was obtained upon storage at −25 °C after concentrating the fully diffused supernatant to *ca*. 5 mL. Yield: 0.641 g, 1.433 mmol, 54%. On one occasion, a sample of **3-Y** contaminated with trace KBH_4_ was recrystallized by slow evaporation of benzene. Several crystals of **3-Y·0.5KBH**_**4**_**·C**_**6**_**H**_**6**_ were identified in the crop by single-crystal XRD, though as a homogeneous sample of the impurity was neither obtained nor sought, no further characterization data are reported. Characterization data for **3-Y**: Anal. Calcd for C_18_H_50_B_2_NSi_2_Y (447.25 g mol^–1^) C, 48.33; H, 11.27, N, 3.13. Found: C, 45.67; H, 10.77, N, 2.81. ^1^H NMR (400.07 MHz, C_6_D_6_): δ 1.13–1.07 (m, 50H, C*H*CH_3_, CHC*H*_3_ and B*H*_4_). ^11^B NMR (128.36 MHz, C_6_D_6_, 298 K): δ −20.7 (br., fwhm ≈ 480 Hz, *B*H_4_), −3.6 (br., fwhm ≈ 2240 Hz, μ-*B*H_4_). ^13^C{^1^H} DEPTQ NMR (100.60 MHz, C_6_D_6_): δ 19.3 (s, *C*H_3_CH), 14.9 (s, CH_3_*C*H). ^29^Si{^1^H} DEPT90 NMR (79.48 MHz, C_6_D_6_): δ −3.7 (s, *Si*^i^Pr_3_). ^1^H NMR (400.07 MHz, C_6_H_4_F_2_, C_6_D_6_): δ 1.13–1.00 (m, 50H, C*H*CH_3_, CHC*H*_3_ and B*H*_4_). ^11^B NMR (128.36 MHz, C_6_H_4_F_2_, C_6_D_6_, 298 K): δ −20.9 (br. p, ^1^*J*_BH_ ≈ 80 Hz, fwhm ≈ 120 Hz, *B*H_4_). ^13^C{^1^H} DEPTQ NMR (100.60 MHz, C_6_H_4_F_2_, C_6_D_6_): δ 18.8 (s, *C*H_3_CH), 14.9 (s, CH_3_*C*H). ^29^Si{^1^H} DEPT90 NMR (79.48 MHz, C_6_H_4_F_2_, C_6_D_6_): δ −3.6 (s, *Si*^i^Pr_3_). FTIR (ATR, microcrystalline): ν̃ = 2945 (m), 2865 (m), 2747 (w), 2519 (w), 2291 (m), 2178 (w), 2142 (w), 1467 (m), 1241 (s), 1194 (s), 908 (s), 873 (s), 715 (s), 655 (s) cm^–1^.

#### [Dy{N(Si^i^Pr_3_)_2_}(BH_4_)(μ-BH_4_)]_4_ (**3-Dy**)

In a long resealable ampule, **2-Dy** (0.3822 g, 0.6446 mmol) was heated to 120 °C in the solid state at *ca*. 0.01 mbar for 2 h, resulting in the partial sublimation of the solid material. After cooling to ambient temperature, the residues were found to have a mass of 0.3357 g (0.6446 mmol assuming the formula weight of **3-Dy**), consistent with the removal of 100% of the bound THF. The ^1^H and ^11^B NMR spectra of this amorphous material were found to be identical to that of an authentic crystalline sample of **3-Dy**, which was prepared as follows. In a long resealable ampule, **2-Dy** (1.770 g, 2.985 mmol) was heated to 150 °C in the solid state at *ca*. 0.01 mbar for 2 h, resulting in the partial sublimation of the solid material and minor production of a high-boiling point colorless oil. After cooling to ambient temperature, in a glovebox, the residues were returned to the base of the flask and the above procedure repeated. After cooling to ambient temperature, the residues were extracted into 1,2-C_6_H_4_F_2_ (3 × 5 mL), filtered, and the solution was concentrated to *ca*. 5 mL. Slow diffusion of excess *n*-hexane (*ca*. 20 mL) at −25 °C afforded the title complex as pale-yellow plates, which were isolated and thoroughly dried in vacuo. An additional crop of the title complex was obtained upon storage at −25 °C after concentrating the fully diffused supernatant to *ca*. 5 mL. Yield: 1.067 g, 2.049 mmol, 67%. As described for **3-Y**, trace KBH_4_ contamination led to the formation of several crystals of **3-Dy·0.5KBH**_**4**_**·C**_**6**_**H**_**4**_**F**_**2**_**-1,2** following recrystallization from 1,2-C_6_H_4_F_2_ layered with hexane. This complex was characterized by single-crystal XRD only. Characterization data for **3-Dy**: Anal. Calcd for C_18_H_50_B_2_DyNSi_2_ (520.84 g mol^–1^) C, 41.50; H, 9.67, N, 2.69. Found: C, 39.55; H, 9.81, N, 2.55. χ*T* product = 12.5 cm^3^ mol^–1^ K, μ_eff_ = 10.0 μ_B_ mol^–1^ (Evans method). ^1^H NMR (400.07 MHz, C_6_D_6_): δ 1.71 (br, fwhm ≈ 20 Hz, C*H*_3_), 1.25 (br, fwhm ≈ 30 Hz), 0.34 (br, fwhm ≈ 30 Hz). ^11^B{^1^H} NMR (128.36 MHz, C_6_D_6_): δ −9.94 (vbr, fwhm ≈ 2450 Hz, B*H*_4_). FTIR (ATR, microcrystalline): ν̃ = 2947 (m), 2867 (m), 2751 (w), 2517 (w), 2287 (m), 2166 (w), 2143 (w), 1467 (m), 1208 (s), 1189 (s), 904 (s), 873 (s), 715 (s), 656 (s) cm^–1^.

#### [Y{N(Si^i^Pr_3_)_2_}_2_(BH_4_)] (**4-Y**)

A solution of **3-Y** (0.447 g, 1.000 mmol) and [K{N(Si^i^Pr_3_)_2_}] (0.734 g, 2.000 mmol) in benzene (10 mL) was stirred at 30 °C for 72 h. The volatiles were then removed in vacuo and the residues extracted into *n*-hexane and filtered. The volatiles were again removed in vacuo, and [HNEt_3_][Al{OC(CF_3_)_3_}_4_] (0.535 g, 0.500 mmol) was added followed by benzene (10 mL). The resulting suspension was vigorously stirred for 4 h at 20 °C, after which the volatiles were removed in vacuo, and the residues were extracted into HMDSO (3 × 1 mL) and filtered. Storage of the solution at −35 °C afforded the title compound as colorless blocks, which were isolated and thoroughly dried in vacuo. Yield: 0.178 g, 0.234 mmol, 23% with respect to **3-Y**. Anal. Calcd for C_36_H_88_BN_2_Si_4_Y (761.08 g mol^–1^) C, 56.81; H, 11.65, N, 3.68. Found: C, 51.98; H, 11.17; N, 2.12. ^1^H NMR (400.07 MHz, C_6_D_6_): δ 1.47 (qd, ^1^*J*_BH_ = 72 Hz, ^1^*J*_YH_ = 14 Hz, 4H, B*H*_4_), 1.35 (d, ^3^*J*_HH_ = 7.4 Hz, 72H, C*H*_3_), 1.06 (sept, ^3^*J*_HH_ = 7.5 Hz, 12H, C*H*). ^11^B NMR (128.36 MHz, C_6_D_6_): δ −21.41 (p, ^1^*J*_BH_ = 86 Hz, *B*H_4_). ^13^C{^1^H} DEPTQ NMR (100.60 MHz, C_6_D_6_): δ 21.0 (s, *C*H_3_CH), 19.8 (s, CH_3_*C*H). ^29^Si{^1^H} DEPT90 NMR (79.48 MHz, C_6_D_6_): δ −3.36 (s, *Si*^i^Pr_3_). FTIR (ATR, microcrystalline): ν̃ = 2943 (s), 2864 (s), 2756 (w), 2731 (w), 2495 (w), 2229 (br), 1463 (m), 1241 (s), 1216 (s), 941 (s), 879 (s), 698 (s), 658 (s) cm^–1^.

#### [Dy{N(Si^i^Pr_3_)_2_}_2_(BH_4_)] (**4-Dy**)

A solution of **3-Dy** (0.520 g, 1.000 mmol) and [K{N(Si^i^Pr_3_)_2_}] (0.734 g, 2.000 mmol) in benzene (10 mL) was stirred at 30 °C for 72 h. The volatiles were then removed in vacuo and the residues extracted into *n*-hexane and filtered. The volatiles were again removed in vacuo, and [HNEt_3_][Al{OC(CF_3_)_3_}_4_] (0.535 g, 0.500 mmol) was added followed by benzene (10 mL). The resulting suspension was vigorously stirred for 4 h at 20 °C, after which the volatiles were removed in vacuo and the residues extracted into HMDSO (3 × 1 mL) and filtered. Storage of the solution at −35 °C afforded the title compound as colorless blocks, which were isolated and thoroughly dried in vacuo. Yield: 0.089 g, 0.117 mmol, 12% with respect to **3-Dy**. Anal. Calcd for C_36_H_88_BDyN_2_Si_4_ (834.67 g mol^–1^) C, 51.80; H, 10.63, N, 3.36. Found: C, 49.16; H, 10.73; N, 2.97. χ*T* product = 12.6 cm^3^ mol^–1^ K, μ_eff_ = 10.1 μ_B_ mol^–1^ (Evans method). ^1^H NMR (400.07 MHz, C_6_D_6_): δ 1.35 (br, fwhm ≈ 25 Hz), 0.62 (br, fwhm ≈ 18 Hz). ^11^B{^1^H} NMR (128.36 MHz, C_6_D_6_): δ −14.1 (vbr, fwhm ≈ 1690 Hz, *B*H_4_). FTIR (ATR, microcrystalline): ν̃ = 2943 (s), 2864 (s), 2753 (w), 2727 (w), 2488 (w), 2240 (br), 1463 (m), 1239 (s), 1212 (s), 939 (s), 879 (s), 698 (s), 659 (s) cm^–1^.

#### [Y{N(Si^i^Pr_3_)_2_}{N(Si^i^Pr_3_)[Si(^i^Pr)_2_{CH(Me)CH_2_}]-κ^2^-N,C}] (**5-Y**)

The HMDSO-insoluble residues (250 mg), obtained from the synthesis of **3-Y** similar to that outlined above and found by ^1^H NMR spectroscopy to contain 11.3 mol % **1-Y** (42.4 mg, 0.0247 mmol) and 88.7 mol % [HNEt_3_][Al{OC(CF_3_)_3_}_4_] (207.6 mg, 0.194 mmol), were combined with [K{N(Si^i^Pr_3_)_2_}] (150 mg, 0.408 mmol) and the mixture suspended in benzene (5 mL). After stirring at ambient temperature for 1 h, the volatiles were removed in vacuo and the residues extracted into hexane. The volatiles were again removed in vacuo, and a portion of the resulting colorless oil containing a mixture of the title compound and HN(Si^i^Pr_3_)_2_ was analyzed in C_6_D_6_ solution by NMR spectroscopy. ^1^H NMR (400.07 MHz, C_6_D_6_): 1.67 (d, ^3^*J*_HH_ = 6.2 Hz, 3H, Si^i^Pr_2_CH(C*H*_3_)CH_2_Y), 1.45 (d, ^3^*J*_HH_ = 6.9 Hz, 3H, Si^*i*^*Pr*_2_CH(CH_3_)CH_2_Y), 1.43 (d, ^3^*J*_HH_ = 6.5 Hz, 3H, Si^*i*^*Pr*_2_CH(CH_3_)CH_2_Y), 1.38–1.32 (m, 9H, C*H*_3_), 1.30–1.25 (m, 54H, C*H*_3_), 1.12–1.02 (obsc. m, 9H, C*H*), 1.00–0.90 (m, 3H, C*H*). ^13^C{^1^H} DEPTQ NMR (100.60 MHz, C_6_D_6_, selected signals): δ 55.34 (d, ^1^*J*_YC_ = 47.4 Hz, Si^i^Pr_2_CH(CH_3_)*C*H_2_Y), 24.50 (s, Si^i^Pr_2_CH(*C*H_3_)CH_2_Y), 21.79 (s, Si^*i*^*Pr*_2_CH(CH_3_)CH_2_Y), 20.69 (s, Si^*i*^*Pr*_2_CH(CH_3_)CH_2_Y). ^29^Si{^1^H} DEPT90 NMR (79.48 MHz, C_6_D_6_): δ, −3.32 (s, ^i^Pr_3_*Si*NSi^i^Pr_2_CH(CH_3_)CH_2_), −5.75 (s, ^i^Pr_3_*Si*N*Si*^i^Pr_3_), −7.62 (s, ^i^Pr_3_SiN*Si*^i^Pr_2_CH(CH_3_)CH_2_).

## Data Availability

Research data files supporting this publication are available from FigShare at https://figshare.com/doi/10.6084/m9.figshare.23807376.

## Supporting Information Available

Additional experimental details, materials, methods, and associated data (PDF)

## Accession Codes

CCDC 2284722–2284732 contain the supplementary crystallographic data for this paper. These data can be obtained free of charge via www.ccdc.cam.ac.uk/data_request/cif, or by emailing data_request@ccdc.cam.ac.uk, or by contacting The Cambridge Crystallographic Data Centre, 12 Union Road, Cambridge, CB2 1EZ, UK; fax: +44 1223 336033.
