# Supplementary material for: Isolation of a Bent Dysprosium Bis(amide) Single-Molecule Magnet
Source: J Am Chem Soc. 2024 Jan 29;146(5):3331–42. doi: 10.1021/jacs.3c12427 (PMC10859956; doi:10.1021/jacs.3c12427)
Supplement: Supplementary file 1 — ja3c12427_si_001.pdf [file ja3c12427_si_001.pdf]

Supplementary Information for:

Isolation of a bent dysprosium bis(amide) single-molecule magnet

Jack Emerson-King,[1] Gemma K. Gransbury,[1] George F. S. Whitehead,[1] Iñigo J. Vitorica-Yrezabal,[1] Mathieu Rouzières,[2] Rodolphe Clérac,[2,*] Nicholas F. Chilton,[1,3,*] and David P. Mills[1,*]

[1]*Department of Chemistry, The University of Manchester, Oxford Road, Manchester, M13 9PL, UK.*

[2]*Univ. Bordeaux, CNRS, CRPP, UMR 5031, 33600 Pessac, France.*

[3]*Research School of Chemistry, The Australian National University, Sullivans Creek Road, Canberra, ACT, 2601, Australia.*

Contents

1. Modified synthesis of [KN(Si^{i}Pr$_3$)$_2$] S3

2. NMR spectra S5

2.1 HN(Si^{i}Pr$_3$)$_2$ S5

2.2 [KN(Si^{i}Pr$_3$)$_2$] S5

2.3 [Y{N(Si^{i}Pr$_3$)$_2$}$_2$][Al{OC(CF$_3$)$_3$}$_4$] (1-Y) S6

2.4 [Dy{N(Si^{i}Pr$_3$)$_2$}$_2$][Al{OC(CF$_3$)$_3$}$_4$] (1-Dy) S7

2.5 [Y{N(Si^{i}Pr$_3$)$_2$}(BH$_4$)$_2$(THF)] (2-Y) S8

2.6 [Dy{N(Si^{i}Pr$_3$)$_2$}(BH$_4$)$_2$(THF)] (2-Dy) S10

2.7 [Y{N(Si^{i}Pr$_3$)$_2$}(BH$_4$)(μ-BH$_4$)]$_4$ (3-Y) S12

2.8 [Dy{N(Si^{i}Pr$_3$)$_2$}(BH$_4$)(μ-BH$_4$)]$_4$ (3-Dy) S16

2.9 [Y{N(Si^{i}Pr$_3$)$_2$}$_2$(BH$_4$)] (4-Y) S17

2.10 [Dy{N(Si^{i}Pr$_3$)$_2$}$_2$(BH$_4$)] (4-Dy) S19

2.11 [Y{N(Si^{i}Pr$_3$)$_2$}{N(Si^{i}Pr$_3$)[Si(^{i}Pr)$_2${CH(Me)CH$_2$}]-κ^2-N,C}] (5-Y)/HN(Si^{i}Pr$_3$)$_2$ .. S20

3. IR spectra S21

3.1 [Ln{N(Si^{i}Pr$_3$)$_2$}$_2$][Al{OC(CF$_3$)$_3$}$_4$] (1-Ln) S21

3.2 [Ln{N(Si^{i}Pr$_3$)$_2$}(BH$_4$)$_2$(THF)] (2-Ln) S23

3.3 [Ln{N(Si^{i}Pr$_3$)$_2$}(BH$_4$)(μ-BH$_4$)]$_4$ (3-Ln) S25

3.4 [Ln{N(Si^{i}Pr$_3$)$_2$}$_2$(BH$_4$)] (4-Ln) S27

4. DFT calculated IR spectra S29

5. Single crystal X-ray diffraction S33

6. Powder X-ray diffraction S47

7. Magnetic measurements S49

8. CASSCF-SO calculations S82

9. References S85

1. Modified synthesis of [KN(Si^iPr_3)$_2$]

General considerations. We have previously reported the synthesis of HN(Si^iPr_3)$_2$ and [KN(Si^iPr_3)$_2$].[1] Here we describe a modified synthesis using $NaNH_2$ as a more operationally convenient starting material in place of condensed NH_3, with other synthetic optimizations. The previously reported NMR data are included for completeness. Under dinitrogen, solvents were either refluxed over molten potassium for 4 days (*n*-hexane), or passed over a column of activated alumina (pentanes, THF, toluene). All solvents were degassed, placed under an argon atmosphere, and stored over either a K mirror (*n*-hexane, pentanes, toluene) or over activated 3 Å molecular sieves (THF). All subsequent manipulations were performed under argon using standard Schlenk line techniques, with use of a glove box for storage of $NaNH_2$, KH and [KN(Si^iPr_3)$_2$] under argon. ^{n}BuLi (2.5 M in hexanes) and $NaNH_2$ were purchased from Fisher Scientific and were used as received. KH was purchased from Merck as a dispersion in mineral oil, which was removed prior to use through sequential pentane washes. Si^iPr_3Cl was purchased from Fluorochem, degassed, and stirred over Mg turnings prior to use. 3 Å molecular sieves were activated by heating at 310 °C *in vacuo* for > 6 hours.

HN(Si^iPr_3)$_2$. Si^iPr_3Cl (42.1 mL, 200 mmol) was added cautiously to a stirring suspension of $NaNH_2$ (7.80 g, 200 mmol) in THF (200 mL) at ambient temperature, with appropriate external cooling with an ice bath when required to moderate any resulting exotherm. The reaction mixture was stirred at ambient temperature overnight, and the resultant suspension filtered to afford a solution of $H_2NSi^iPr_3$. ^{n}BuLi (2.5 M in hexane, 80 mL, 200 mmol) was added to this solution, and the reaction mixture warmed with stirring until butane gas evolution was observed (at *ca.* 50-60 ºC). The reaction was held at this temperature until all gas evolution had subsided, typically 1-2 h, affording a solution of $LiHNSi^iPr_3$. Si^iPr_3Cl (42.1 mL, 200 mmol) was added, and the reaction mixture was heated to 70 ºC for 72 hours.

The volatiles were removed *in vacuo* and the residues extracted into pentane (*ca*. 50 mL) and filtered. Following removal of pentane in vacuo, $HN(Si^iPr_3)_2$ was obtained as a colorless oil by fractional distillation of the crude residues (10^{-2} mbar through a Vigreux column, unreacted $^iPr_3SiCl$ removed at *ca*. 40 ºC, $HN(Si^iPr_3)_2$ collected at *ca*. 100 ºC, significant quantities of some non-volatile material remain). Yield: 21.60 g, 65.6 mmol, 33%, formed a white crystalline solid upon standing (melting point *ca*. 25 ºC). ^1H NMR (500 MHz, C_6D_6): δ 1.02–0.87 (complex set of resonances, 42H, $CH(CH_3)_2$ & $(CHCH_3)_2$), –0.37 (s, 1H, N*H*). $^{29}Si\{^1H\}$ DEPT90 NMR (79.48 MHz, C_6D_6): δ 6.06.

[KN(Si^{i}Pr$_3$)$_2$]. A solution of $HN(Si^iPr_3)_2$ (21.6 g, 65.6 mmol) in toluene (100 mL) was added to a stirring suspension of KH (2.62 g, 65.6 mmol) in toluene (100 mL). The reaction mixture was heated until hydrogen evolution was observed (*ca*. 120 °C). The reaction was held at this temperature until gas evolution had ceased (typically 3 h), allowed to cool, and filtered to remove any insoluble K salts. The solution was concentrated to the point of incipient crystallization and layered with excess hexane, which after slow diffusion afforded the title compound as large colorless crystalline blocks. Occasionally, a brown discoloration is observed when using $HN(Si^iPr_3)_2$ containing trace impurities. Two or three recrystallizations as outlined above are sufficient to afford a product with no detectable protic impurities. Yield: 17.90 g, 48.7 mmol, 74%, colorless crystalline solid. ^1H NMR (400.07 MHz, C_6D_6): δ 1.30 (d, $^3J_{HH}$ = 7.4 Hz, 36H, $CHCH_3$), 0.90 (sept, $^3J_{HH}$ = 7.5 Hz, 6H, $CHCH_3$). $^{29}Si\{^1H\}$ DEPT90 NMR (79.48 MHz, C_6D_6): δ 16.35.

2. NMR spectra

2.1 $HN(Si^iPr_3)_2$

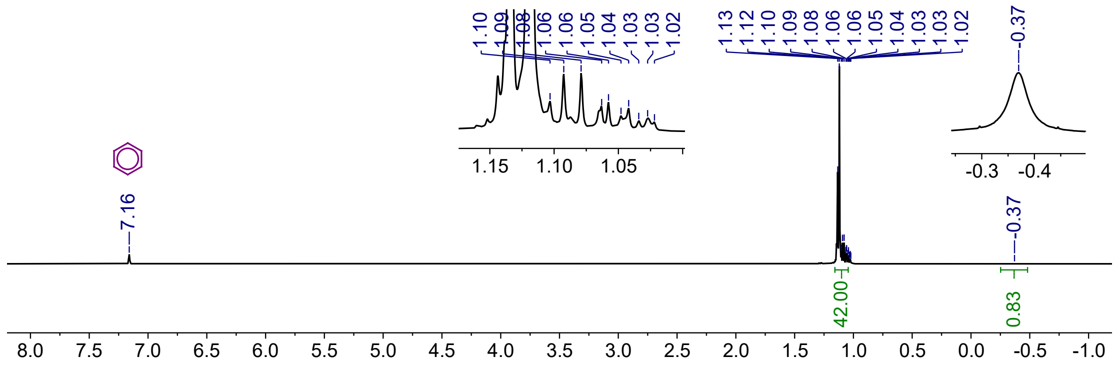

Figure S1. ^1H NMR spectrum (400.07 MHz, C_6D_6) of $HN(Si^iPr_3)_2$ at 298 K.

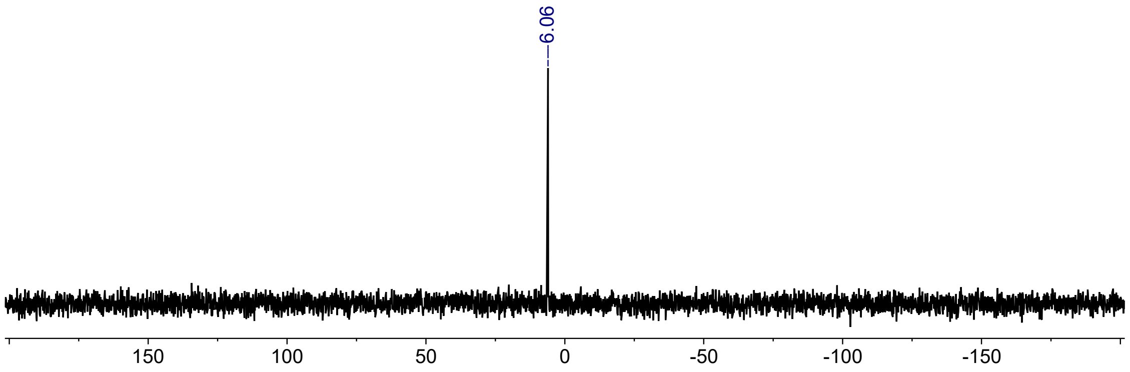

Figure S2. $^{29}Si\{^1H\}$ DEPT90 NMR spectrum (79.48 MHz, C_6D_6) of $HN(Si^iPr_3)_2$ at 298 K.

2.2 $[KN(Si^iPr_3)_2]$

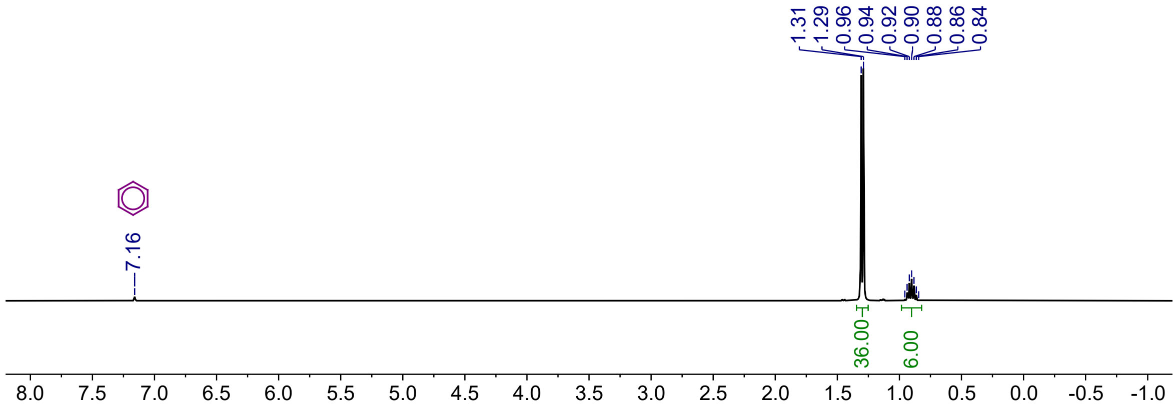

Figure S3. ^1H NMR spectrum (400.07 MHz, C_6D_6) of $[KN(Si^iPr_3)_2]$ at 298 K.

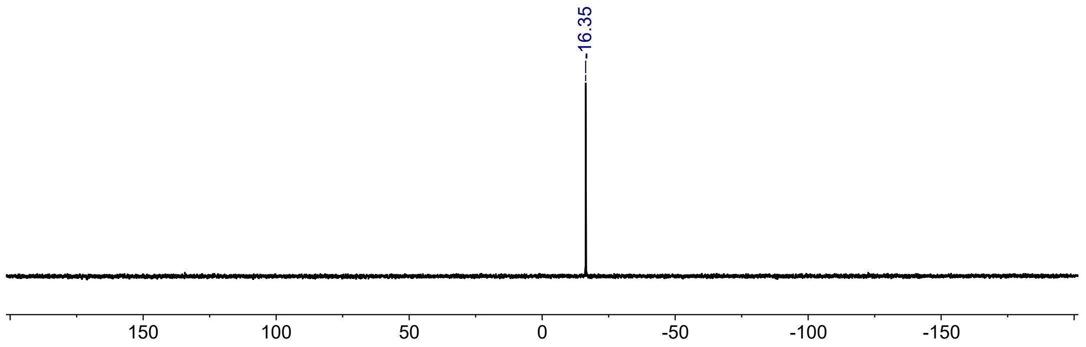

Figure S4. $^{29}Si\{^{1}H\}$ DEPT90 NMR spectrum (79.48 MHz, C_6D_6) of [$KN(Si^iPr_3)_2$] at 298 K.

2.3 [$Y\{N(Si^iPr_3)_2\}_2$][$Al\{OC(CF_3)_3\}_4$] (1-Y)

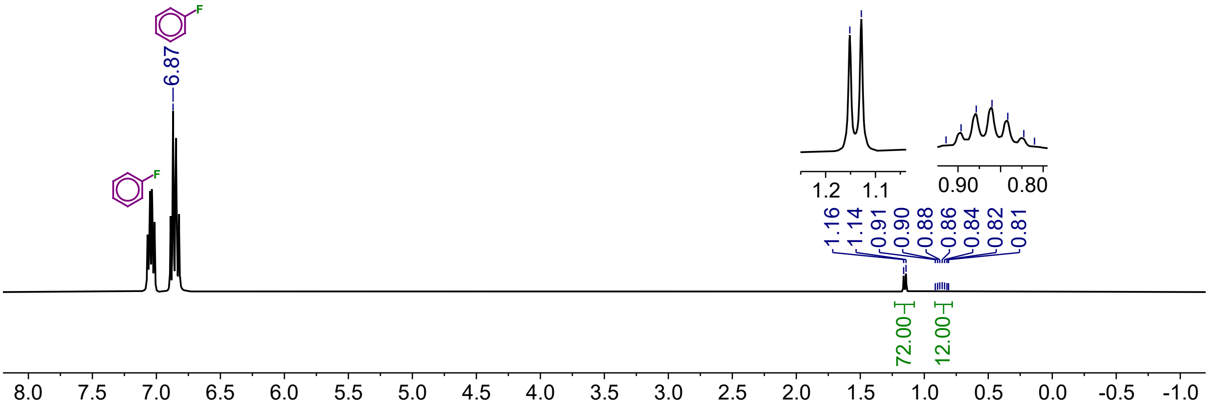

Figure S5. ^{1}H NMR spectrum (400.07 MHz, C_6H_5F) of **1-Y** at 298 K.

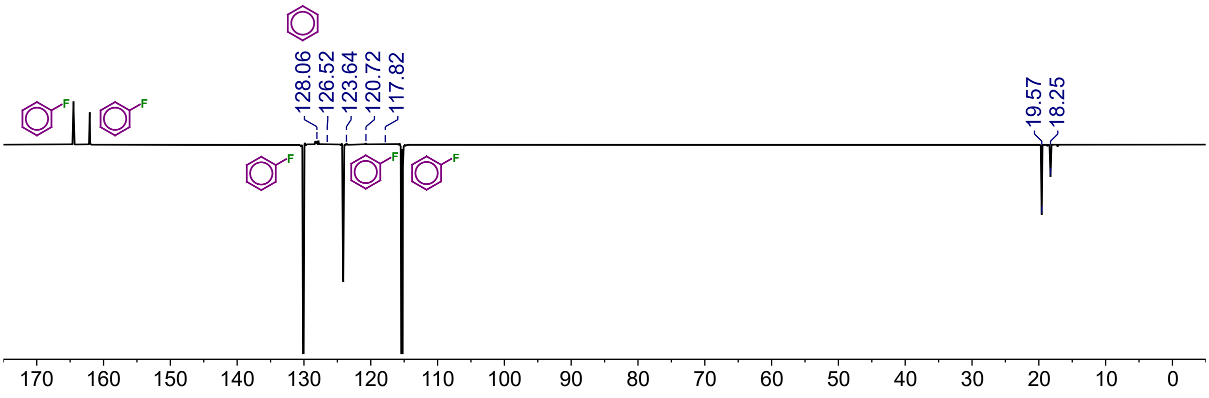

Figure S6. $^{13}C\{^{1}H\}$ DEPTQ NMR spectrum (100.60 MHz, C_6H_5F) of **1-Y** at 298 K.

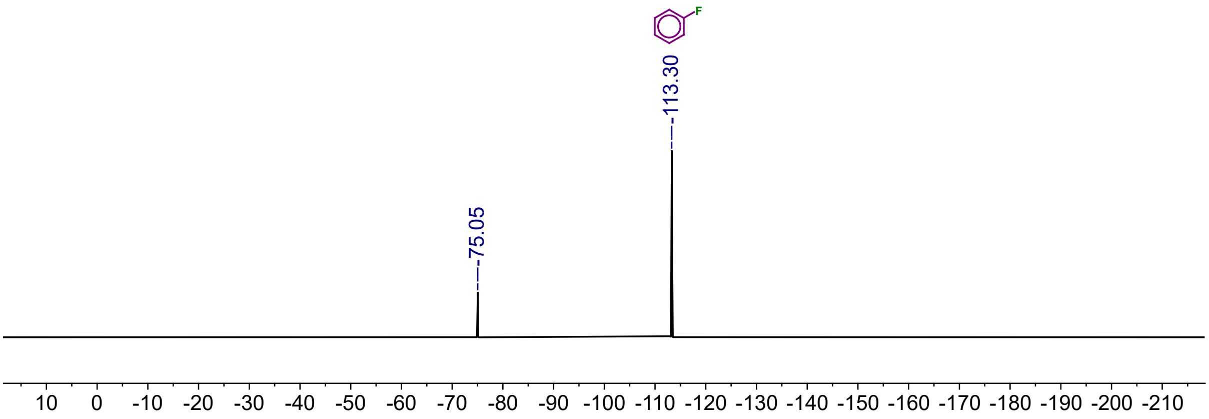

Figure S7. ^{19}F NMR spectrum (376.40 MHz, C_6H_5F) of **1-Y** at 298 K.

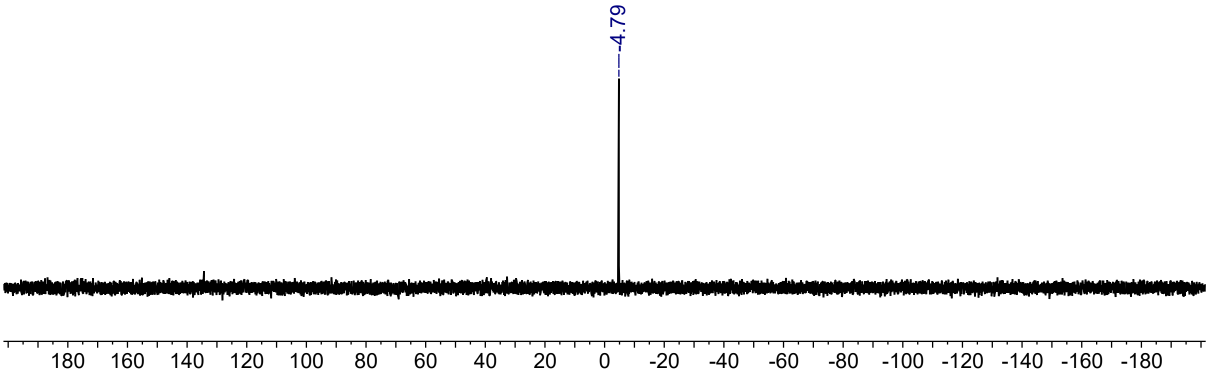

Figure S8. ^{29}Si{^{1}H} DEPT90 NMR spectrum (79.48 MHz, C_6H_5F) of **1-Y** at 298 K.

2.4 [Dy{N(Si^{i}Pr$_3$)$_2$}$_2$][Al{OC(CF$_3$)$_3$}$_4$] (1-Dy)

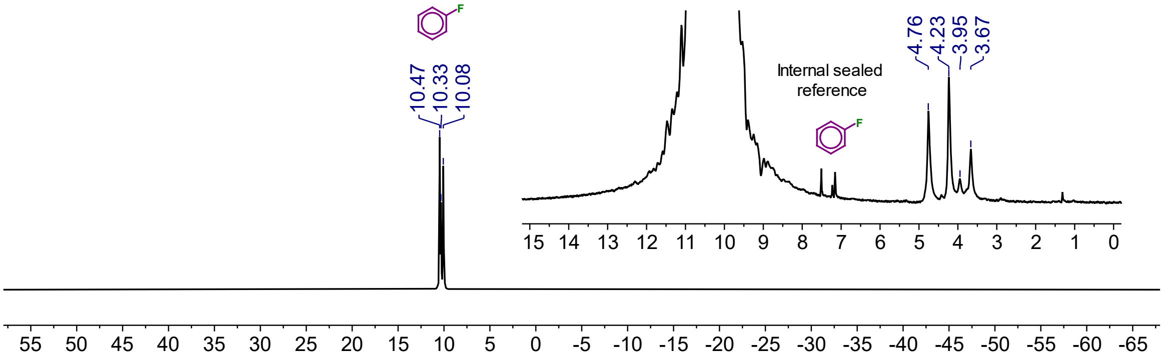

Figure S9. ^{1}H NMR spectrum (400.07 MHz, C_6H_5F) of **1-Dy** at 298 K containing an internal sealed capillary of 1 : 1 C_6D_6 : C_6H_5F.

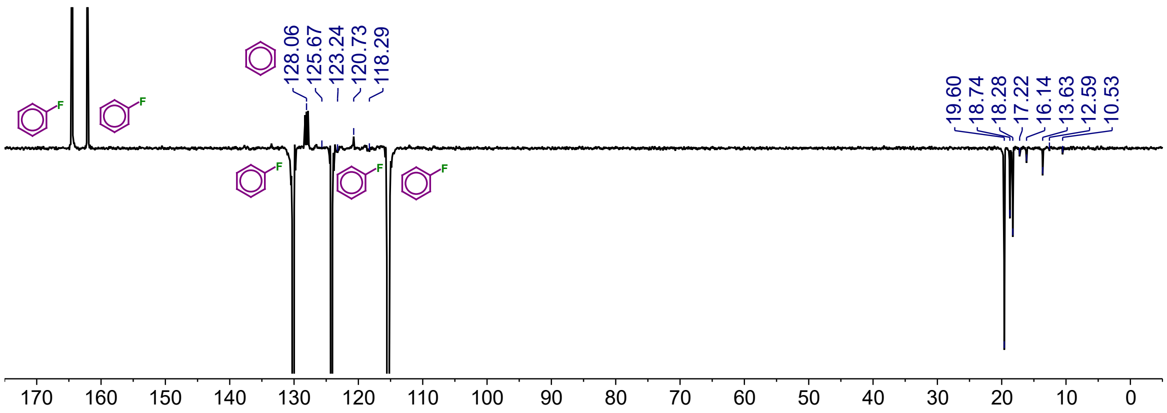

Figure S10. $^{13}C\{^{1}H\}$ DEPTQ NMR spectrum (100.60 MHz, C_6H_5F) of **1-Dy** at 298 K containing an internal sealed capillary of 1 : 1 C_6D_6 : C_6H_5F.

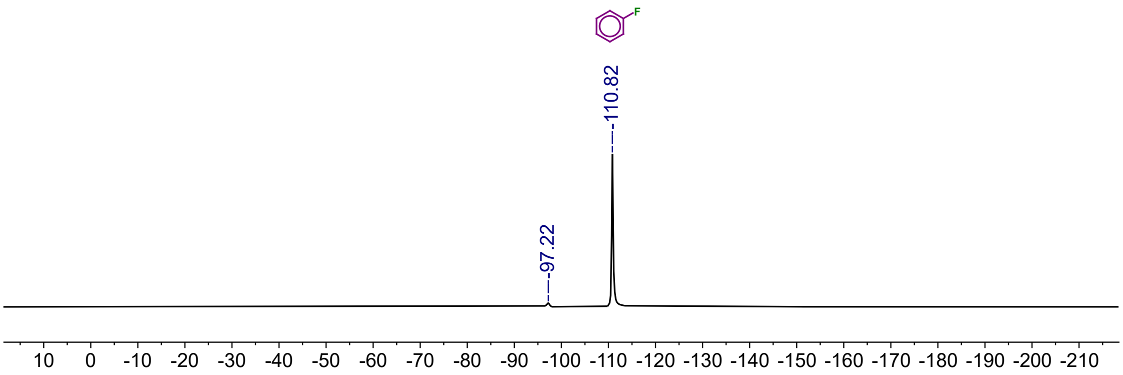

Figure S11. ^{19}F NMR spectrum (376.40 MHz, C_6H_5F) of **1-Dy** at 298 K containing an internal sealed capillary of 1 : 1 C_6D_6 : C_6H_5F.

2.5 [Y{N(Si^{i}Pr$_3$)$_2$}(BH$_4$)$_2$(THF)] (2-Y)

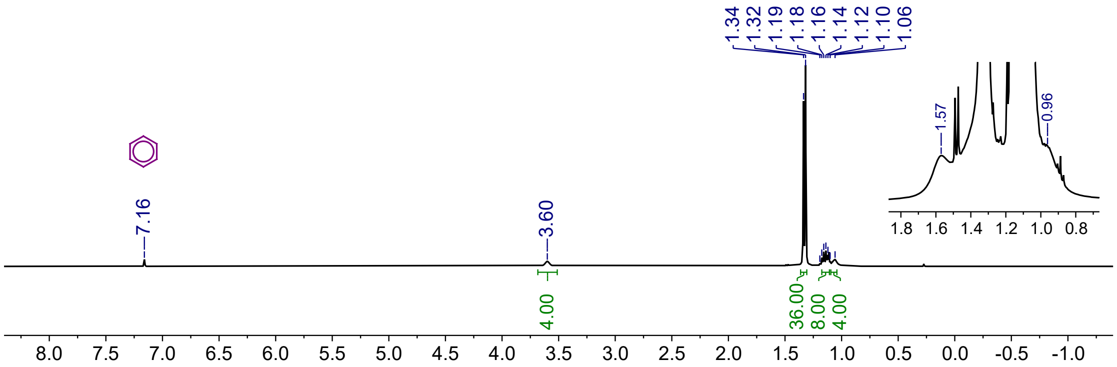

Figure S12. ^{1}H NMR spectrum (400.07 MHz, C_6D_6) of **2-Y** at 298 K.

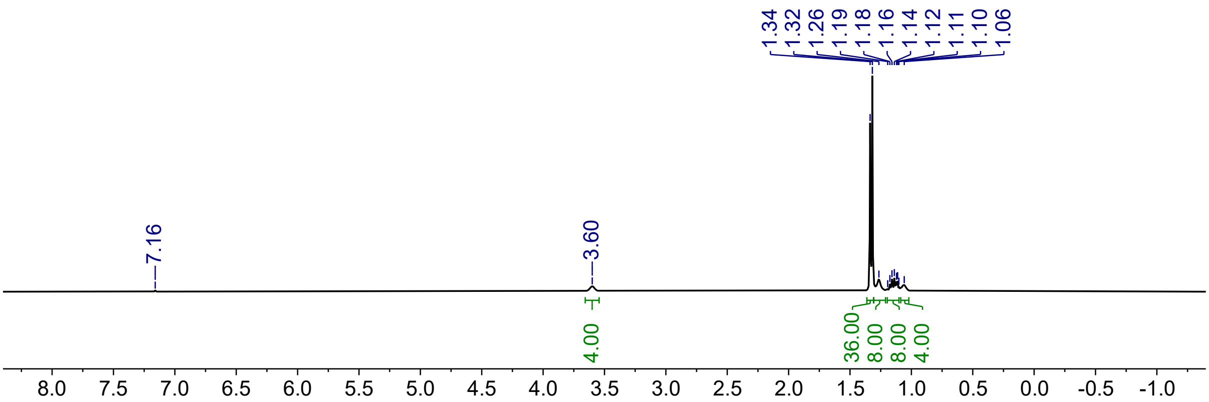

Figure S13. $^{1}H\{^{11}B\}$ NMR spectrum (400.07 MHz, C_6D_6) of **2-Y** at 298 K.

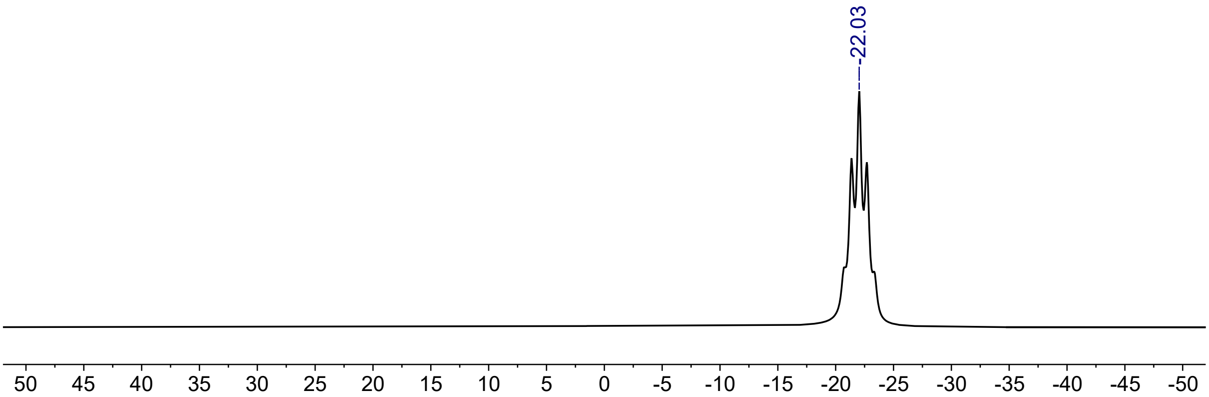

Figure S14. ^{11}B NMR spectrum (128 MHz, C_6D_6) of **2-Y** at 298 K.

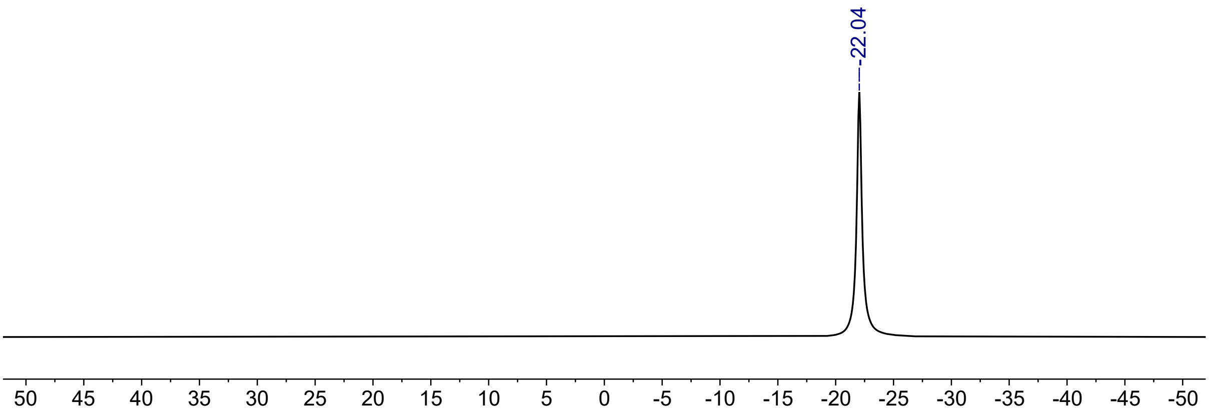

Figure S15. $^{11}B\{^{1}H\}$ NMR spectrum (128 MHz, C_6D_6) of **2-Y** at 298 K.

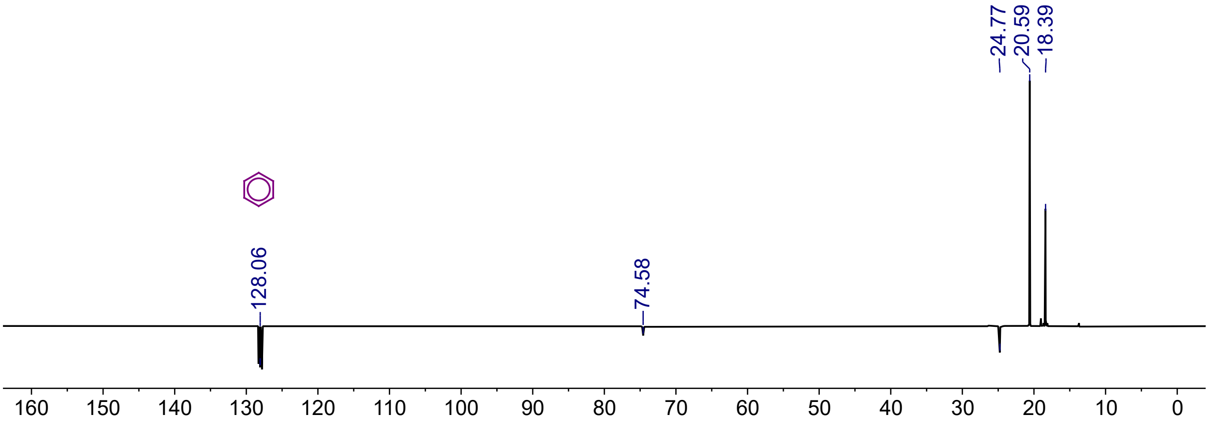

Figure S16. $^{13}C\{^{1}H\}$ DEPTQ NMR spectrum (100.60 MHz, C_6D_6) of **2-Y** at 298 K.

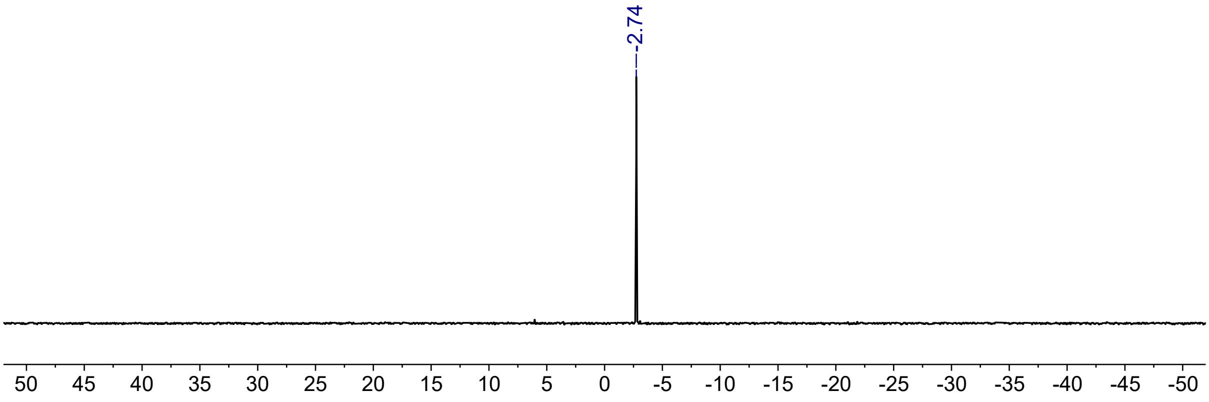

Figure S17. $^{29}Si\{^{1}H\}$ DEPT90 NMR spectrum (79.48 MHz, C_6D_6) of **2-Y** at 298 K.

2.6 [Dy{N(Si^{i}Pr$_3$)$_2$}(BH$_4$)$_2$(THF)] (2-Dy)

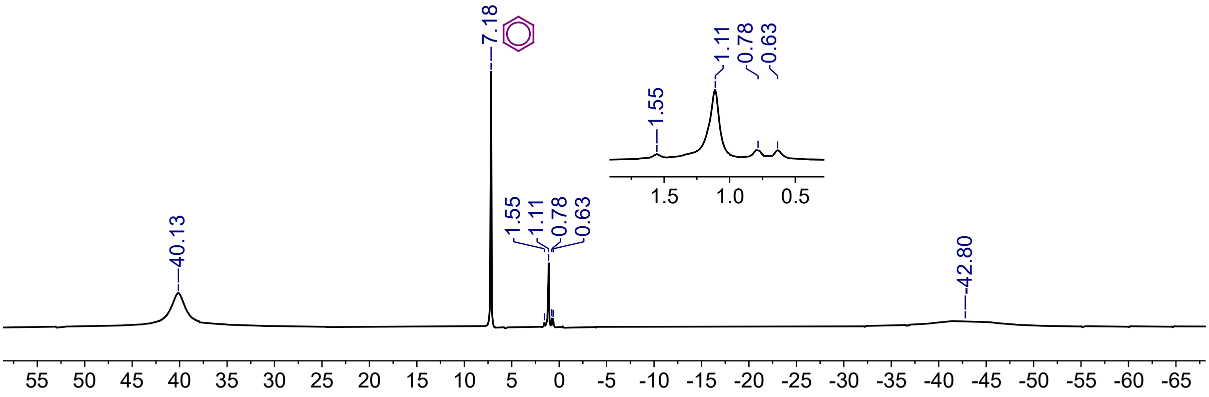

Figure S18. ^{1}H NMR spectrum (400.07 MHz, C_6D_6) of **2-Dy** at 298 K.

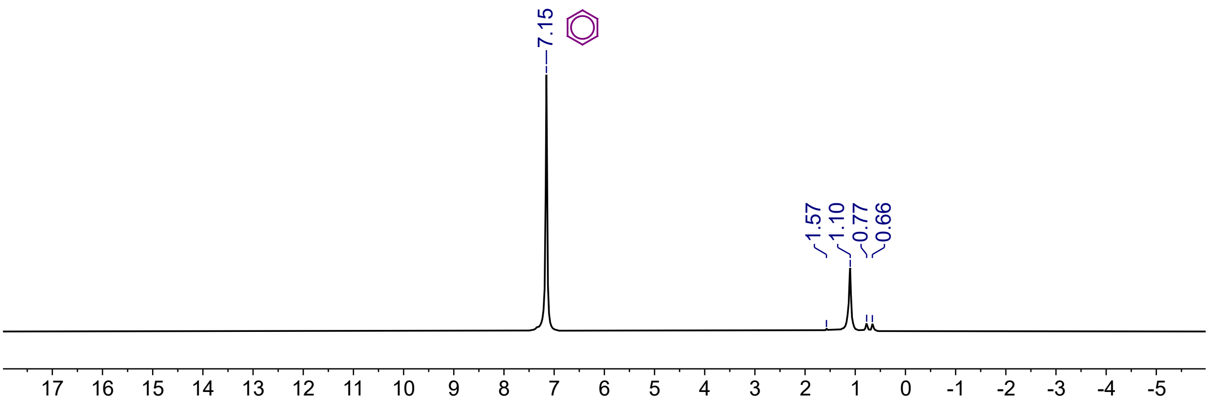

Figure S19. $^{1}H\{^{11}B\}$ NMR spectrum (400.07 MHz, C_6D_6) of **2-Dy** at 298 K.

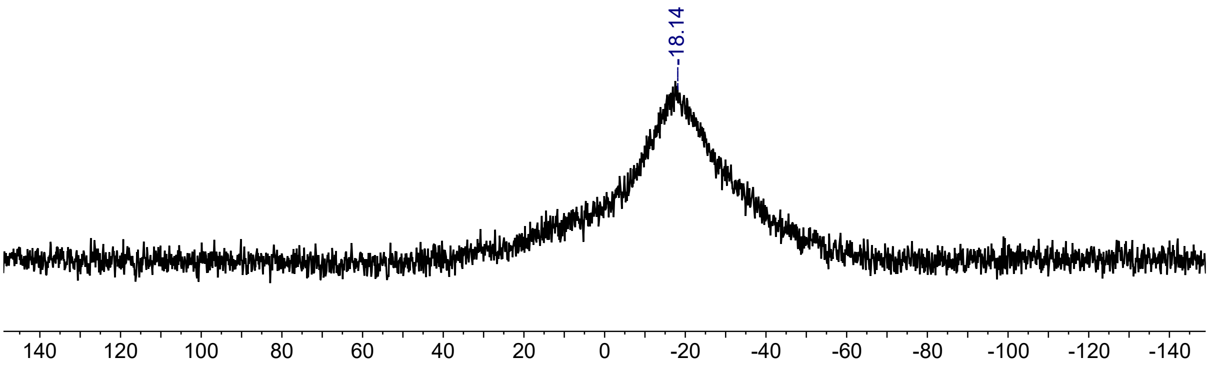

Figure S20. ^{11}B NMR spectrum (128 MHz, C_6D_6) of **2-Dy** at 298 K.

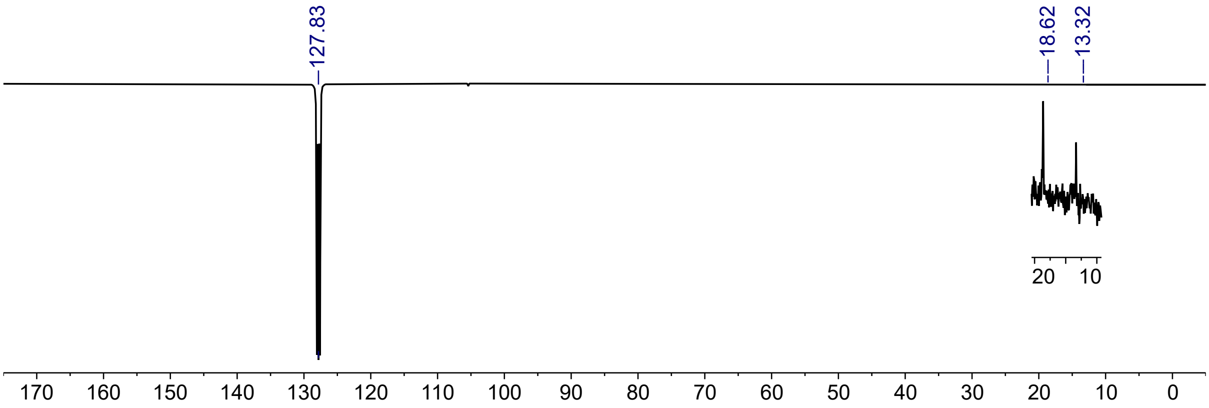

Figure S21. $^{13}C\{^{1}H\}$ DEPTQ NMR spectrum (100.60 MHz, C_6H_5F) of **2-Dy** at 298 K containing an internal sealed capillary of 1 : 1 C_6D_6 : C_6H_5F.

2.7 [Y{N(Si^{i}Pr$_3$)$_2$}(BH$_4$)(μ-BH$_4$)]$_4$ (3-Y)

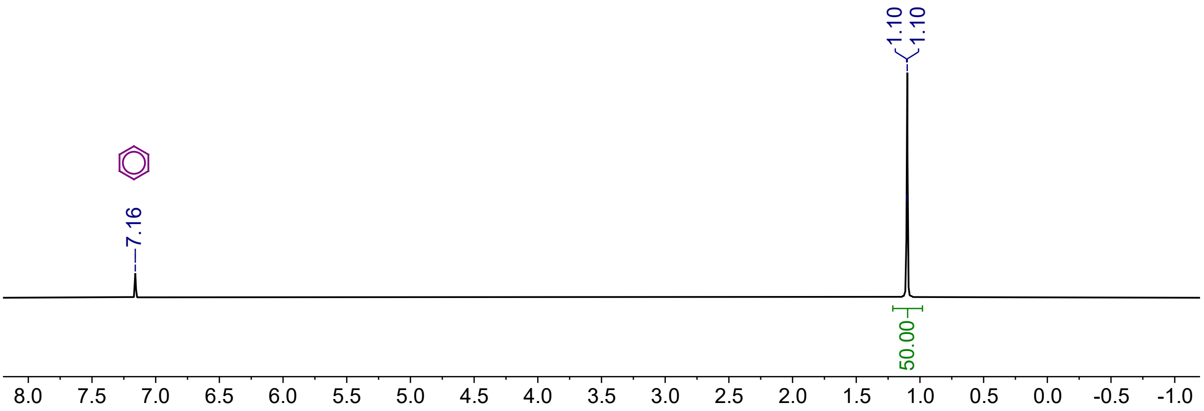

Figure S22. ^{1}H NMR spectrum (400.07 MHz, C_6D_6) of **3-Y** at 298 K.

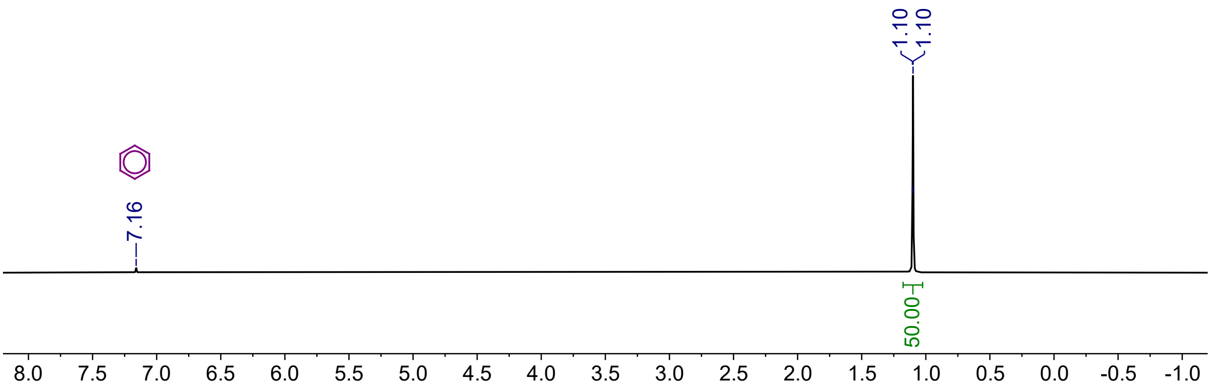

Figure S23. ^{1}H{^{11}B} NMR spectrum (400.07 MHz, C_6D_6) of **3-Y** at 298 K.

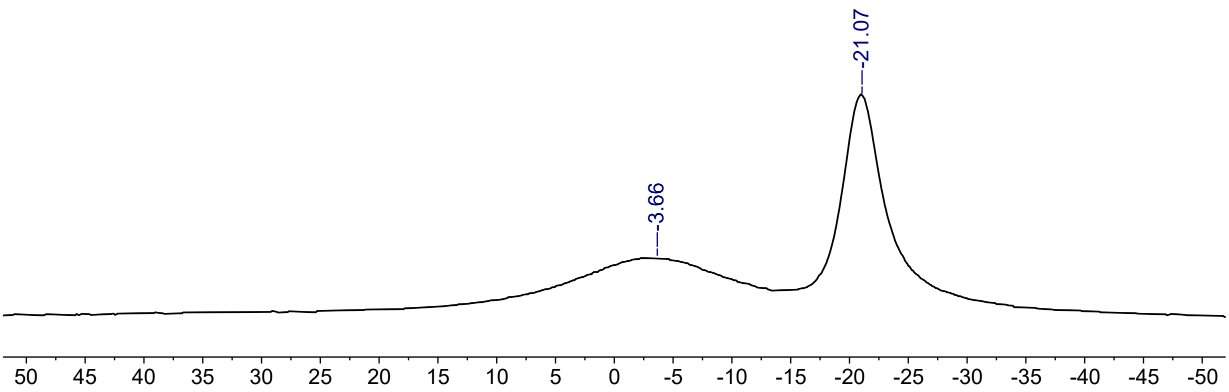

Figure S24. ^{11}B NMR spectrum (128 MHz, C_6D_6) of **3-Y** at 298 K.

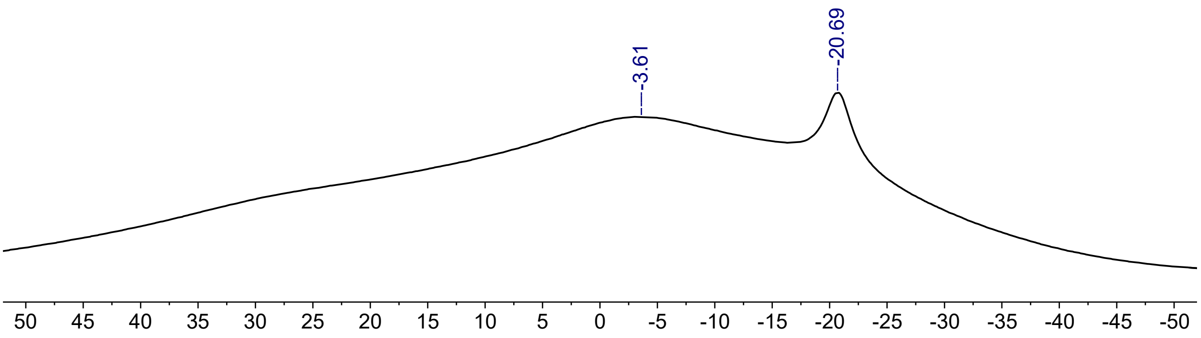

Figure S25. $^{11}B\{^{1}H\}$ NMR spectrum (128 MHz, C_6D_6) of **3-Y** at 298 K.

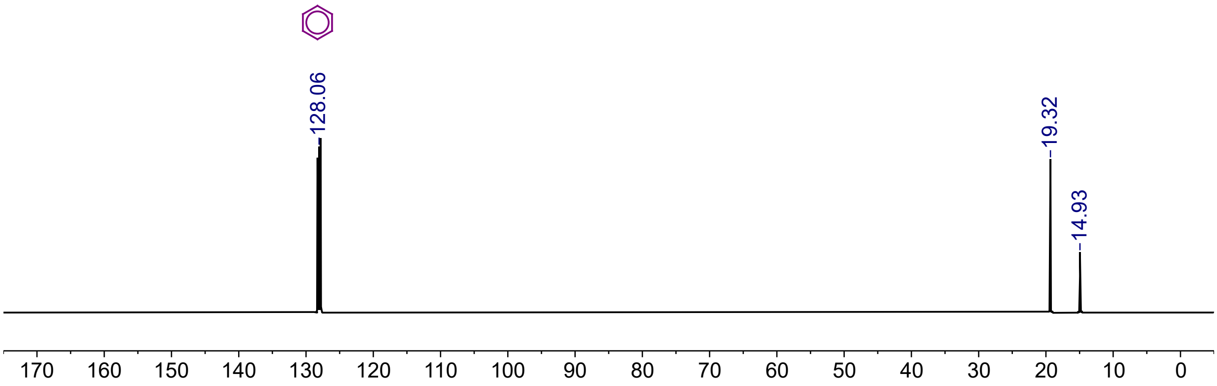

Figure S26. $^{13}C\{^{1}H\}$ DEPTQ NMR spectrum (100.60 MHz, C_6D_6) of **3-Y** at 298 K.

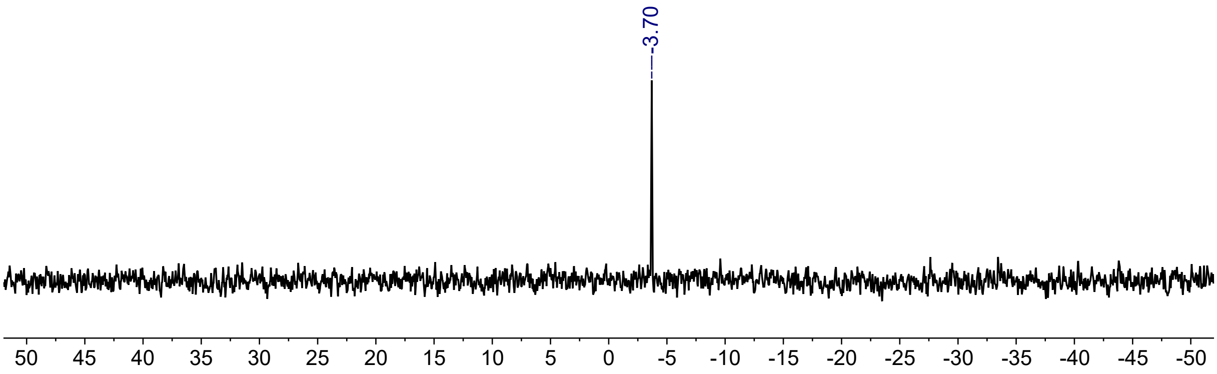

Figure S27. $^{29}Si\{^{1}H\}$ DEPT90 NMR spectrum (79.48 MHz, $C_6H_4F_2$) of **3-Y** at 298 K.

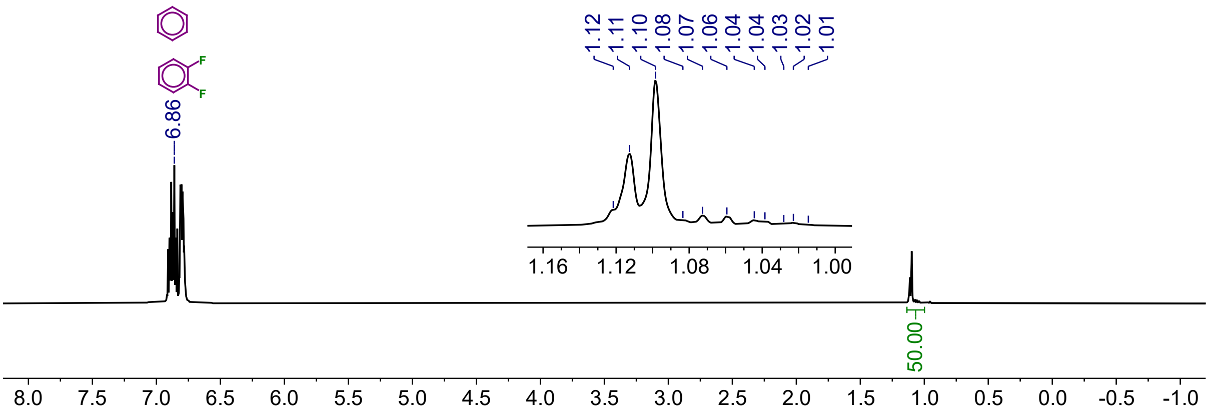

Figure S28. ^{1}H NMR spectrum (400.07 MHz, $C_6H_4F_2$) of **3-Y** at 298 K.

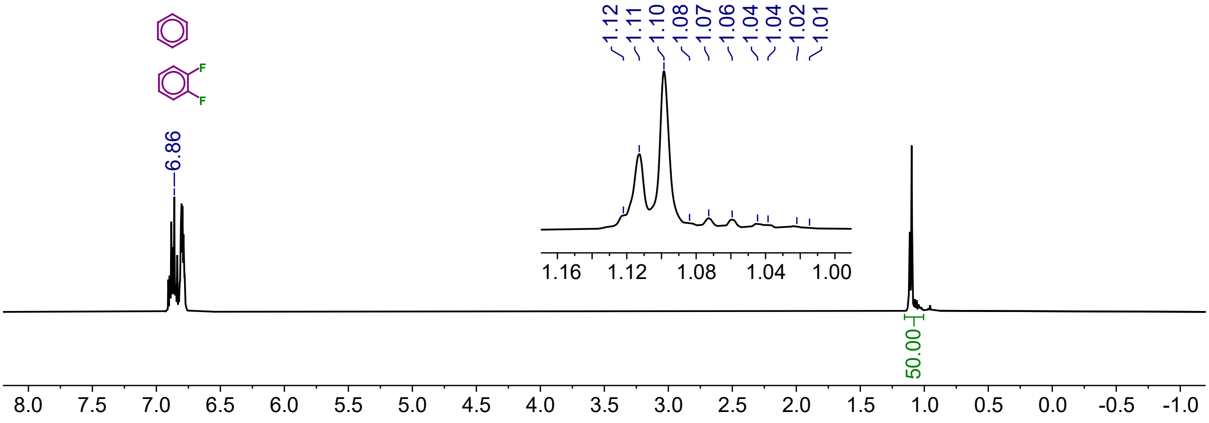

Figure S29. $^{1}H\{^{11}B\}$ NMR spectrum (400.07 MHz, $C_6H_4F_2$) of **3-Y** at 298 K.

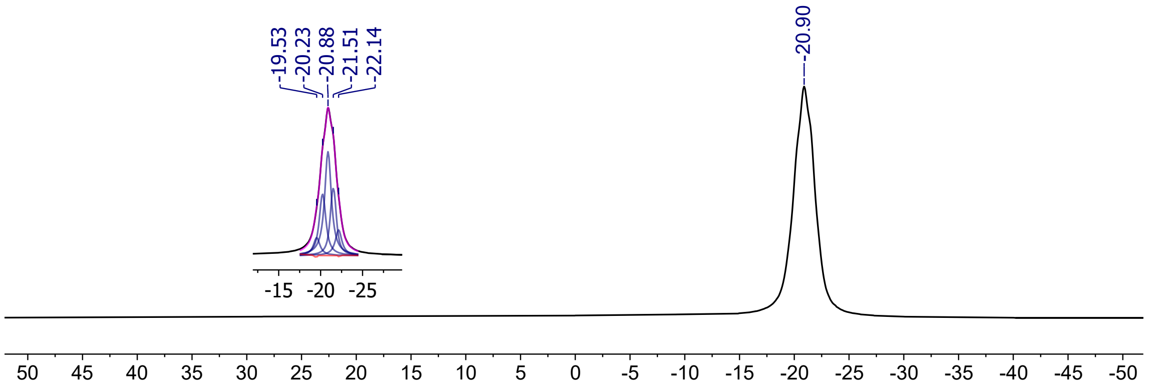

Figure S30. ^{11}B NMR spectrum (128 MHz, $C_6H_4F_2$) of **3-Y** at 298 K. Inset: line fitting of the resonance as a broad pentet, using a generalized-Lorentzian form with no additional constraints, as implemented in MestReNova version 14.3.

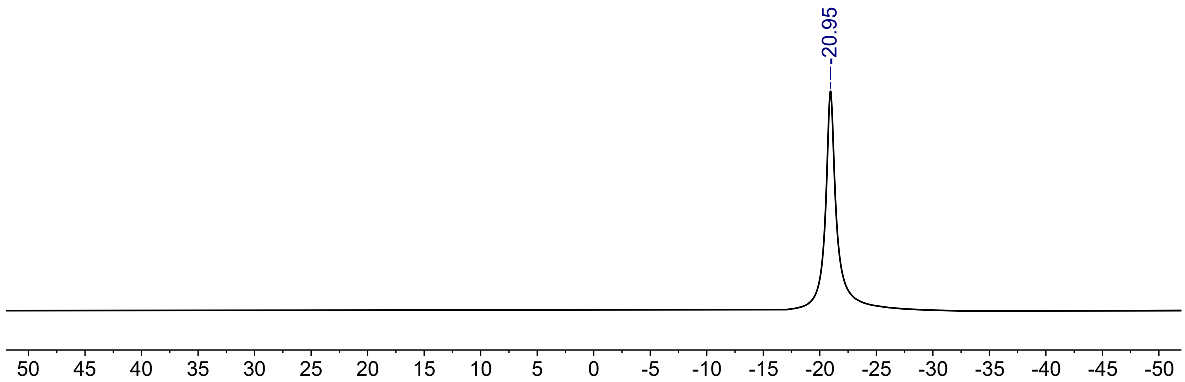

Figure S31. $^{11}B\{^{1}H\}$ NMR spectrum (128 MHz, $C_6H_4F_2$) of **3-Y** at 298 K.

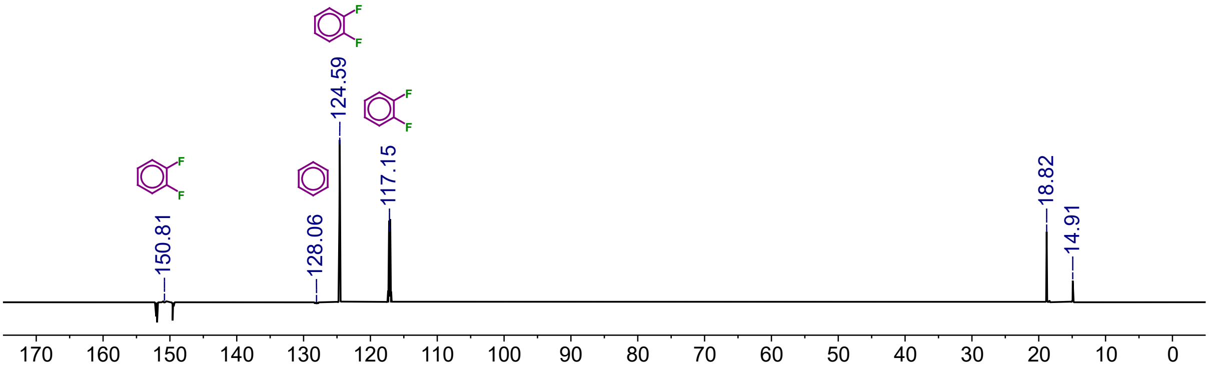

Figure S32. $^{13}C\{^{1}H\}$ DEPTQ NMR spectrum (100.60 MHz, $C_6H_4F_2$) of **3-Y** at 298 K.

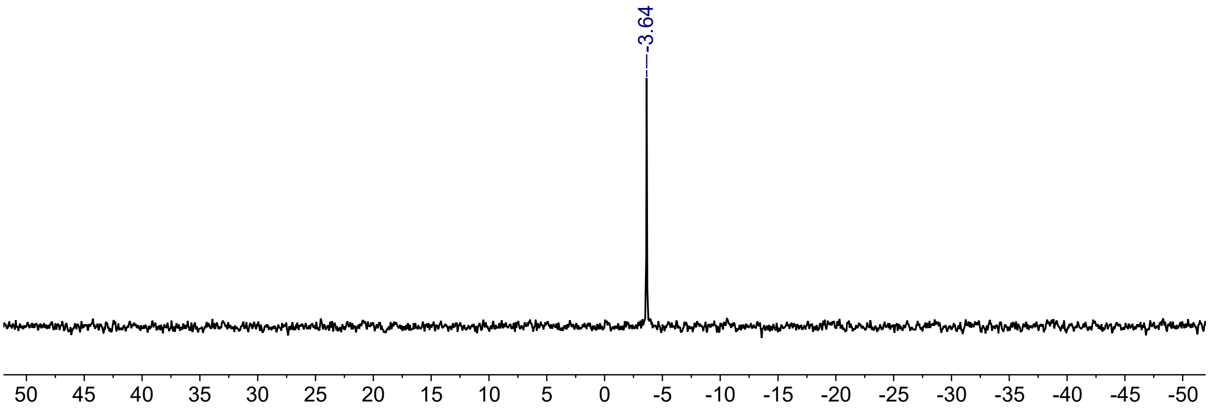

Figure S33. $^{29}Si\{^{1}H\}$ DEPT90 NMR spectrum (79.48 MHz, $C_6H_4F_2$) of **3-Y** at 298 K.

2.8 [Dy{N(Si^{i}Pr$_3$)$_2$}(BH$_4$)(μ-BH$_4$)]$_4$ (3-Dy)

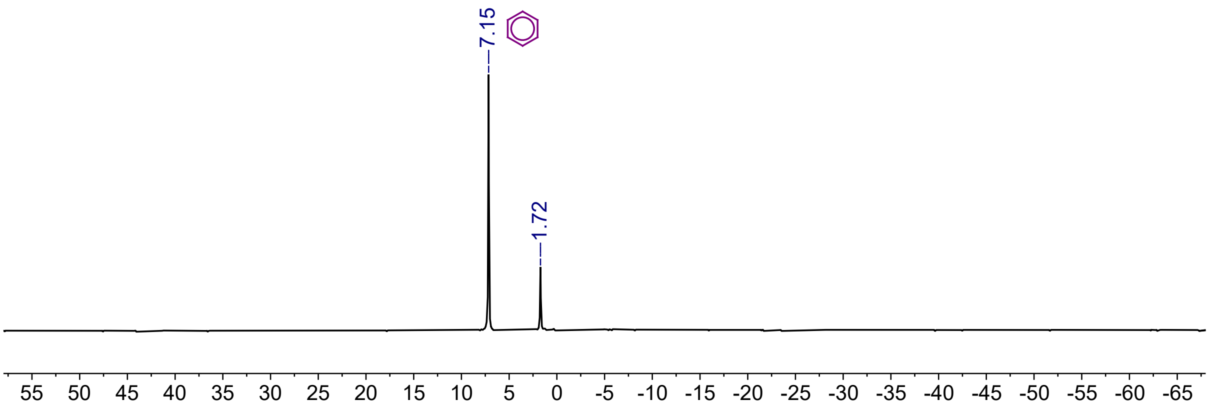

Figure S34. ^{1}H NMR spectrum (400.07 MHz, C_6D_6) of **3-Dy** at 298 K.

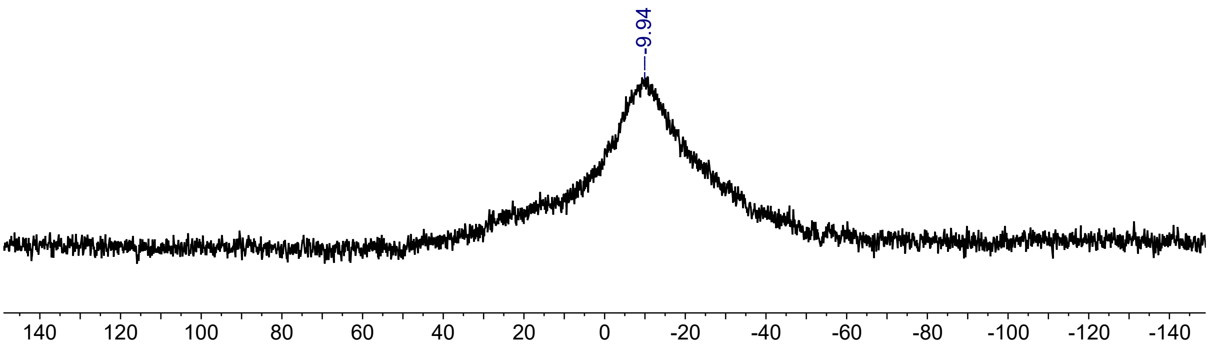

Figure S35. ^{11}B NMR spectrum (128 MHz, C_6D_6) of **3-Dy** at 298 K.

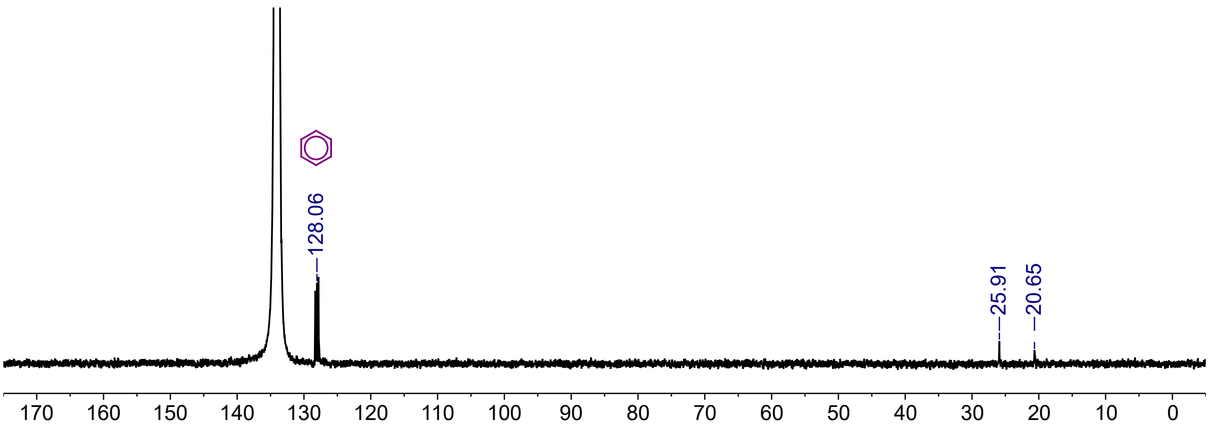

Figure S36. ^{13}C{^{1}H} DEPTQ NMR spectrum (100.60 MHz, C_6D_6) of **3-Dy** at 298 K.

2.9 [Y{N(Si^{i}Pr$_3$)$_2$}$_2$(BH$_4$)] (4-Y)

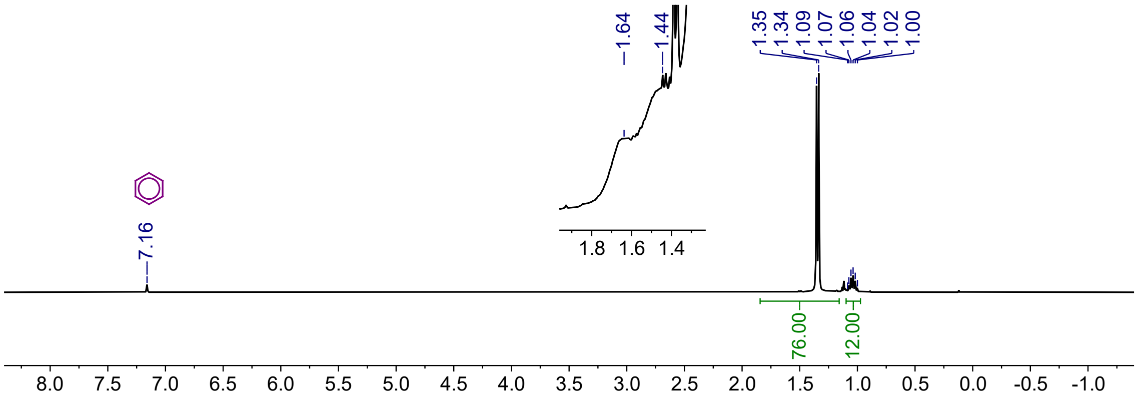

Figure S37. ^{1}H NMR spectrum (400.07 MHz, C_6D_6) of **4-Y** at 298 K.

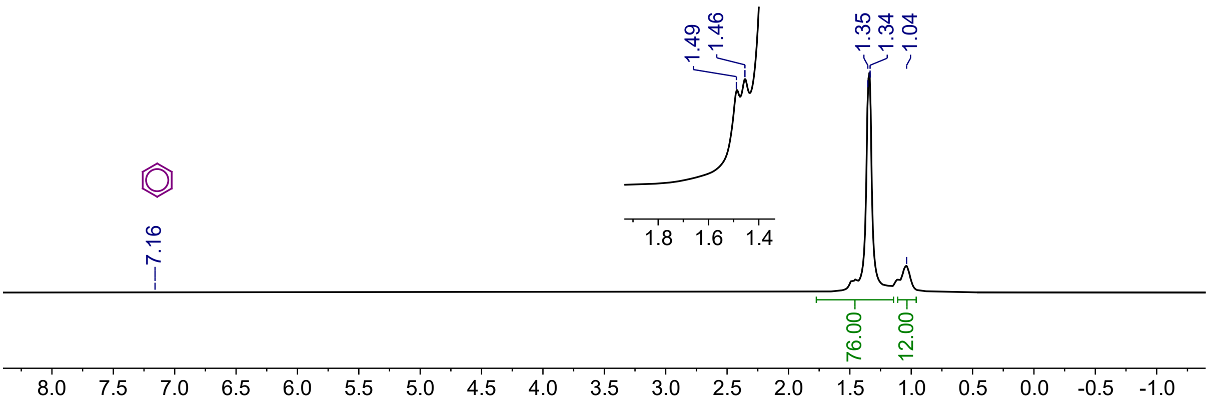

Figure S38. ^{1}H{^{11}B} NMR spectrum (400.07 MHz, C_6D_6) of **4-Y** at 298 K.

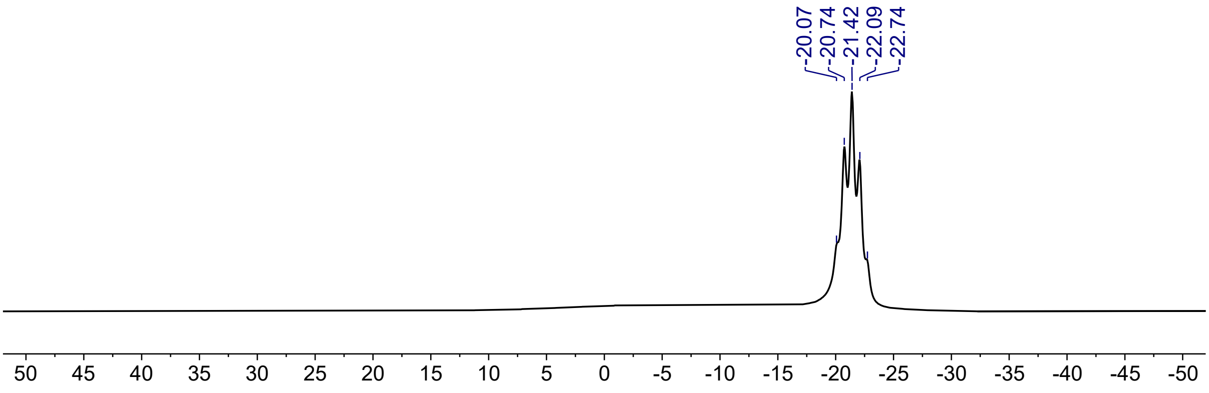

Figure S39. ^{11}B NMR spectrum (128 MHz, C_6D_6) of **4-Y** at 298 K.

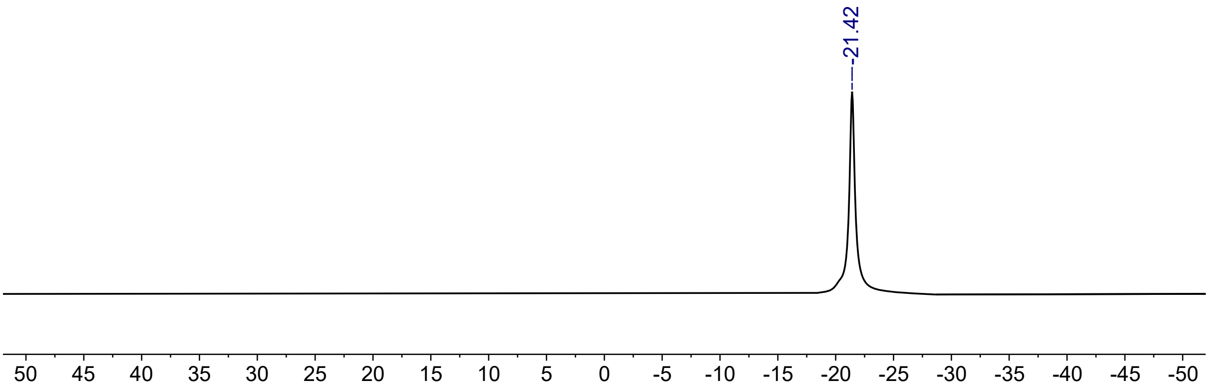

Figure S40. $^{11}B\{^1H\}$ NMR spectrum (128 MHz, C_6D_6) of **4-Y** at 298 K.

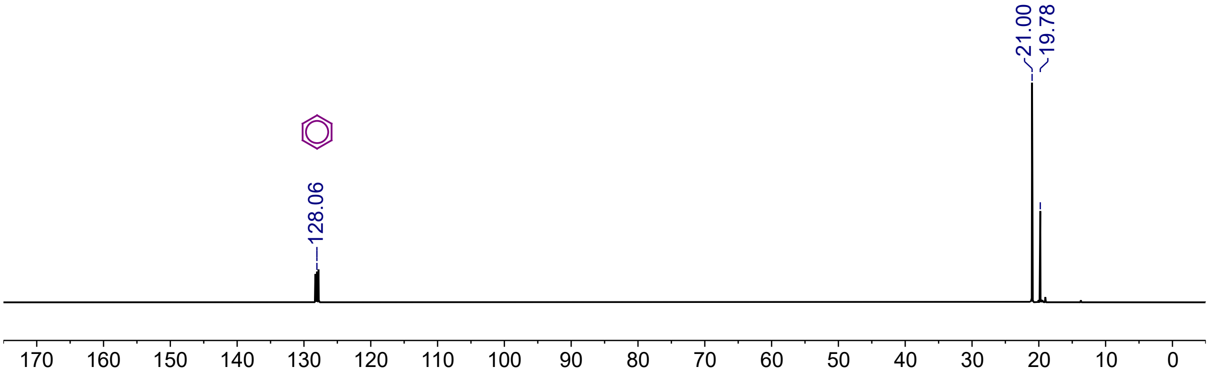

Figure S41. $^{13}C\{^1H\}$ DEPTQ NMR spectrum (100.60 MHz, C_6D_6) of **4-Y** at 298 K.

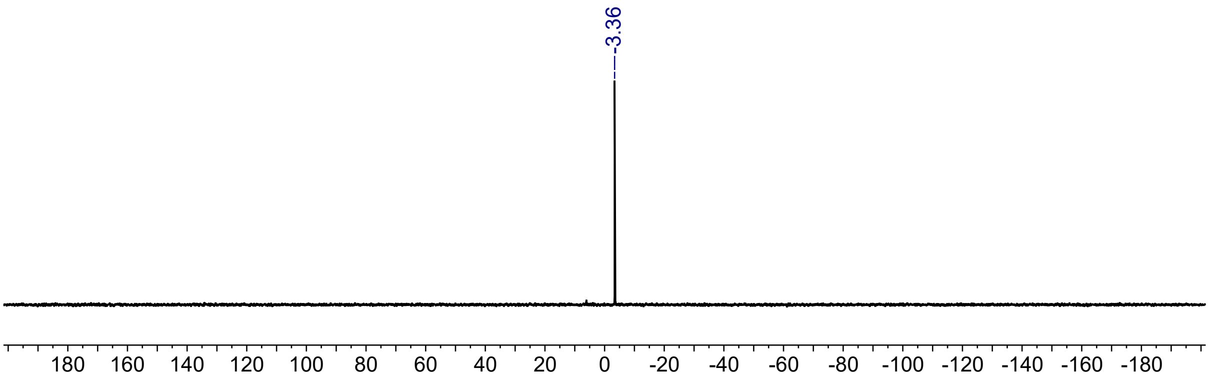

Figure S42. $^{29}Si\{^1H\}$ DEPT90 NMR spectrum (79.48 MHz, C_6D_6) of **4-Y** at 298 K.

2.10 [Dy{N(Si^{i}Pr$_3$)$_2$}$_2$(BH$_4$)] (4-Dy)

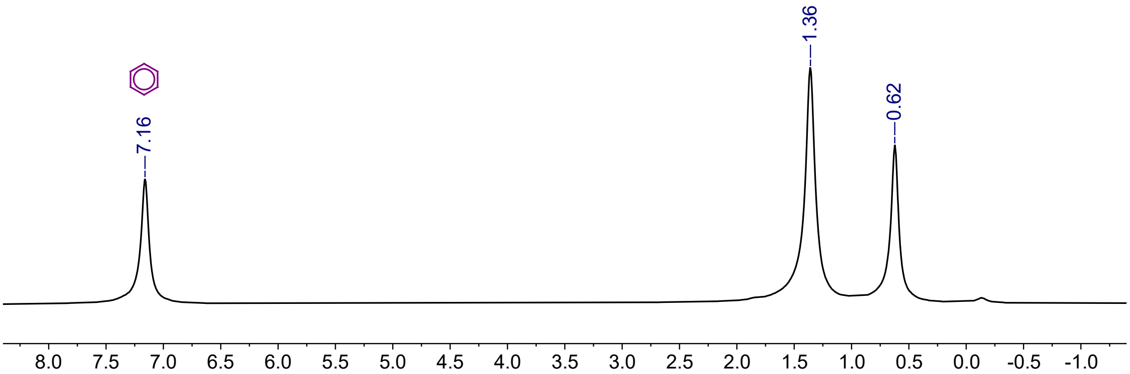

Figure S43. ^{1}H NMR spectrum (400.07 MHz, C_6D_6) of **4-Dy** at 298 K.

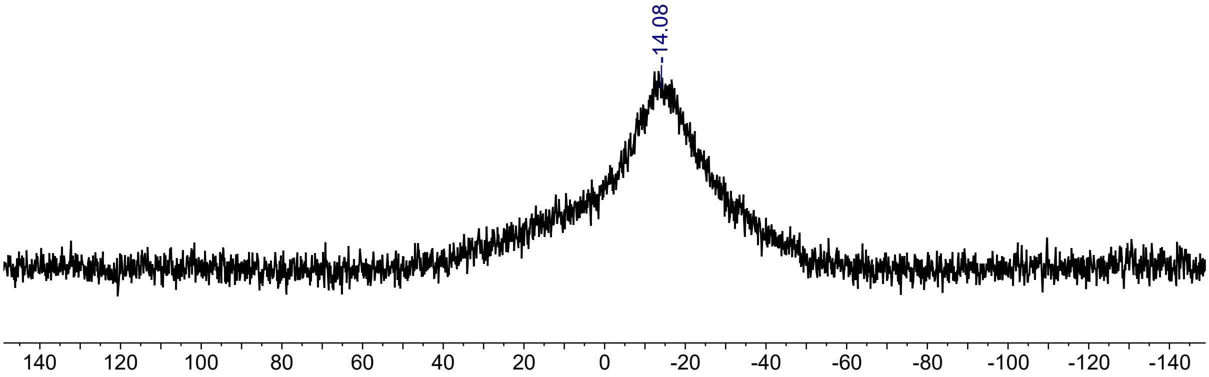

Figure S44. ^{11}B NMR spectrum (128 MHz, C_6D_6) of **4-Dy** at 298 K.

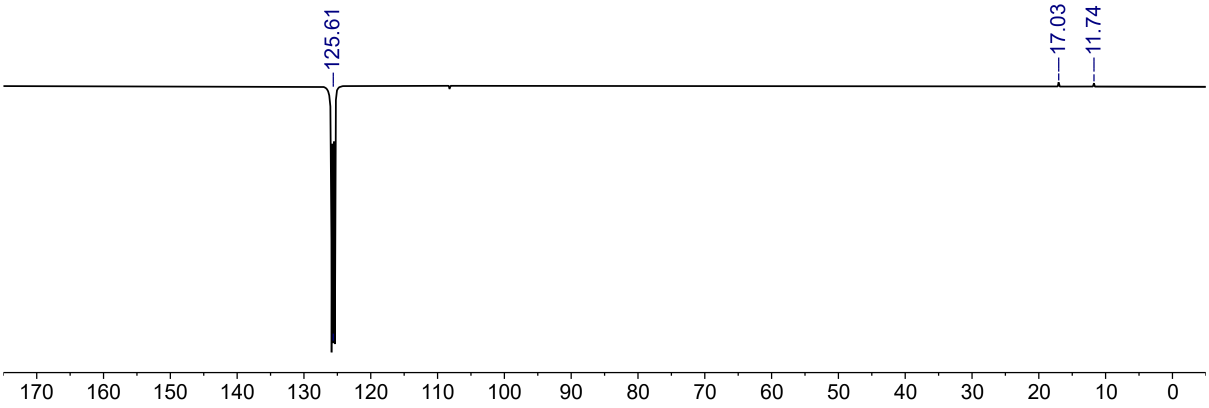

Figure S45. ^{13}C{^{1}H} DEPTQ NMR spectrum (100.60 MHz, C_6D_6) of **4-Dy** at 298 K.

2.11 [Y{N(Si^{i}Pr$_3$)$_2$}{N(Si^{i}Pr$_3$)[Si(^{i}Pr)$_2${CH(Me)CH$_2$}]-κ^2-N,C}] (5-Y)/HN(Si^{i}Pr$_3$)$_2$

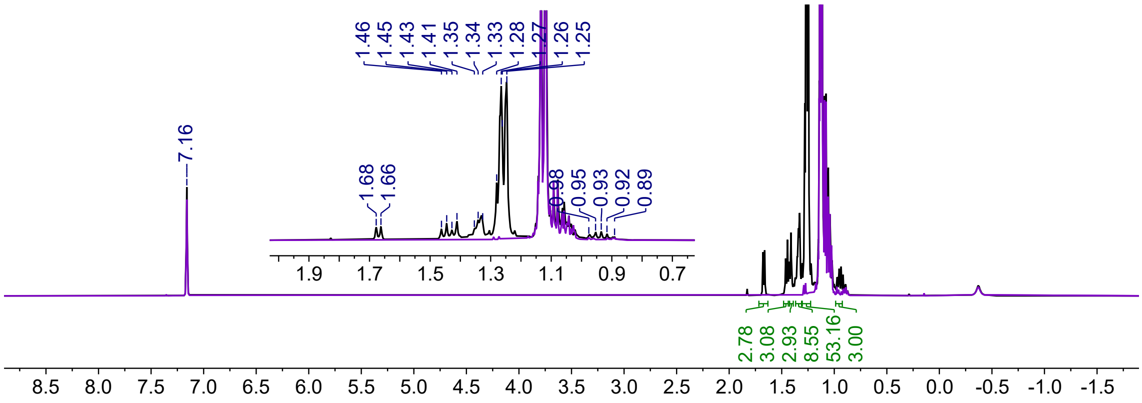

Figure S46. ^{1}H NMR spectrum (400.07 MHz, C_6D_6) of **5-Y** + HN(Si^{i}Pr$_3$)$_2$ at 298 K, overlaid with a spectrum of isolated HN(Si^{i}Pr$_3$)$_2$ (purple) under the same conditions.

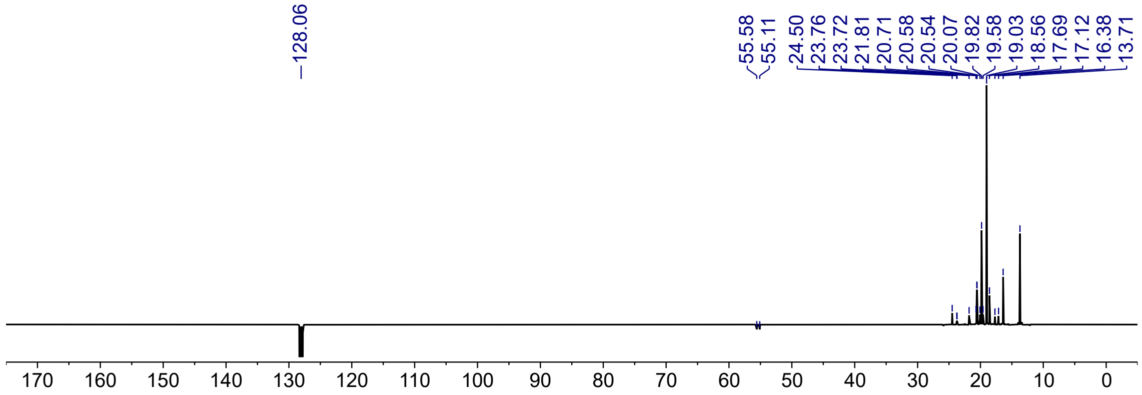

Figure S47. ^{13}C{^{1}H} DEPTQ NMR spectrum (100.60 MHz, C_6D_6) of **5-Y** + HN(Si^{i}Pr$_3$)$_2$ at 298 K.

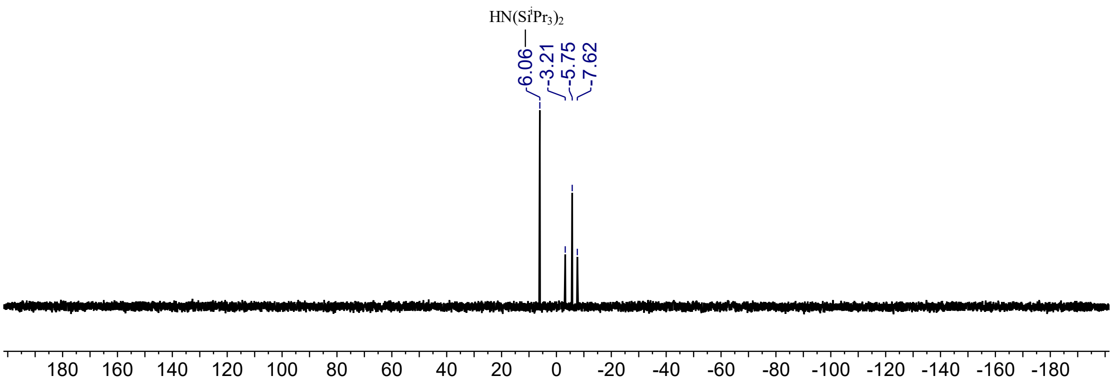

Figure S48. ^{29}Si{^{1}H} DEPT90 NMR spectrum (79.48 MHz, C_6D_6) of **5-Y** + HN(Si^{i}Pr$_3$)$_2$ at 298 K.

3. IR spectra

3.1 [Ln{N(Si^{i}Pr$_3$)$_2$}$_2$][Al{OC(CF$_3$)$_3$}$_4$] (1-Ln)

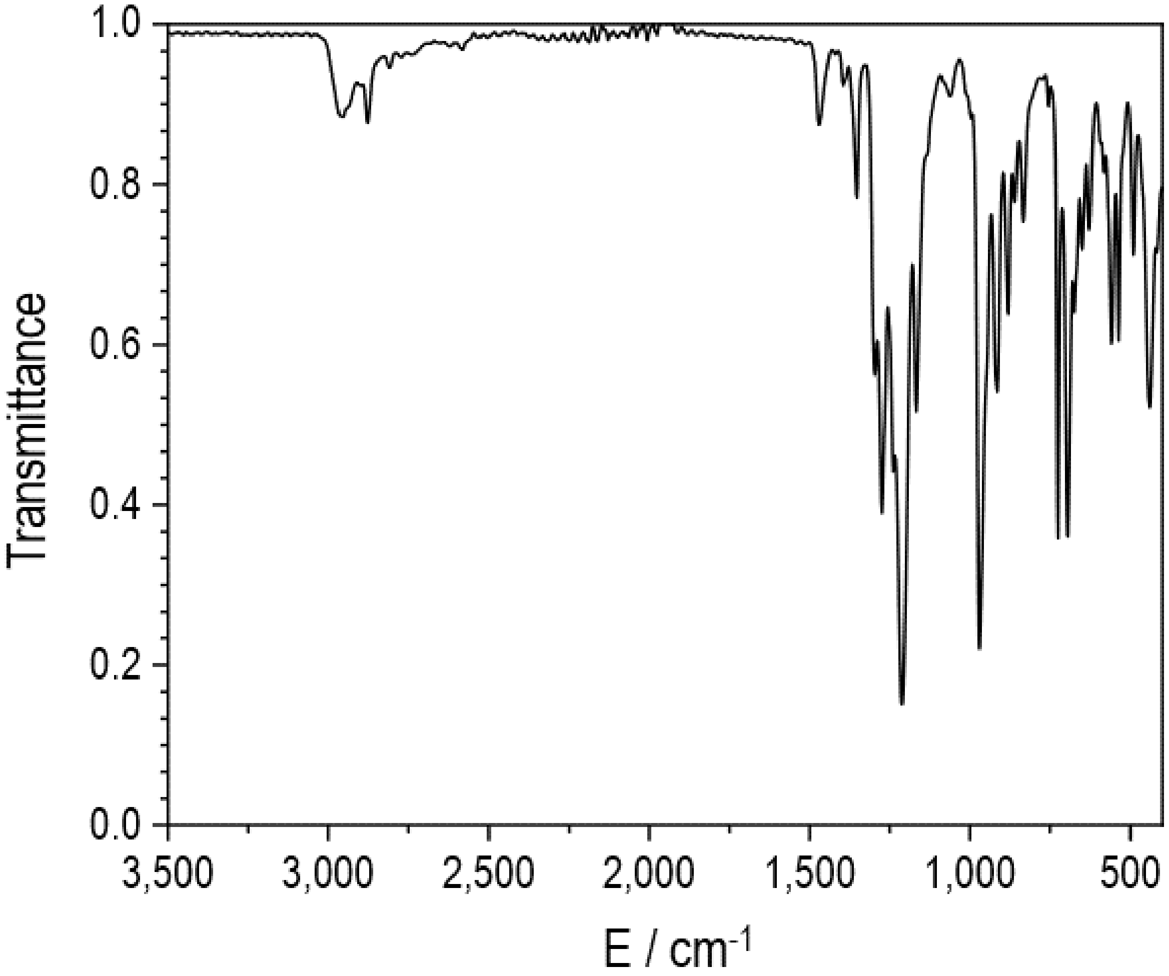

Figure S49. FT-IR (ATR, microcrystalline solid) spectrum of **1-Y** at ambient temperature.

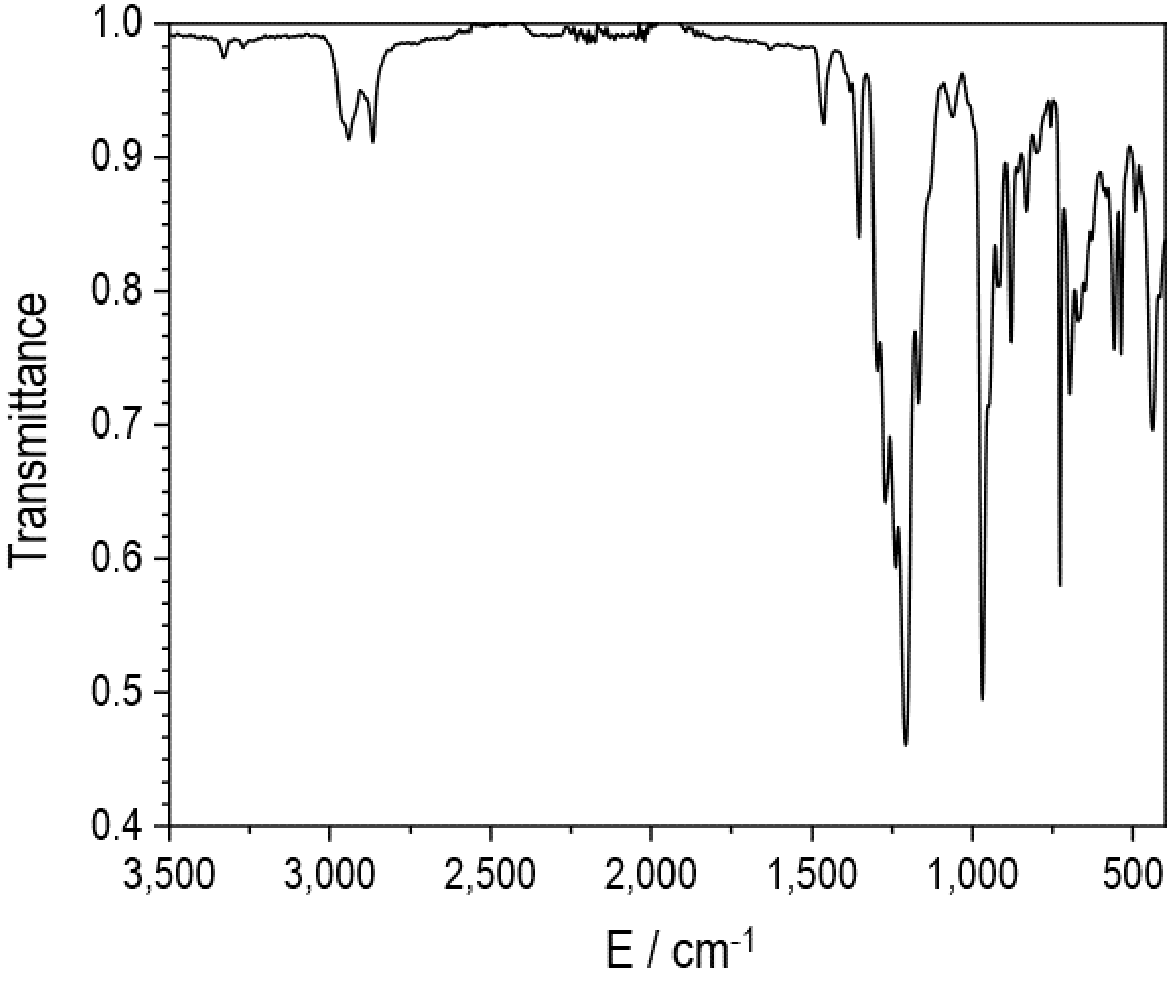

Figure S50. FT-IR (ATR, microcrystalline solid) spectrum of **1-Dy** at ambient temperature.

3.2 [Ln{N(Si^{i}Pr$_3$)$_2$}(BH$_4$)$_2$(THF)] (2-Ln)

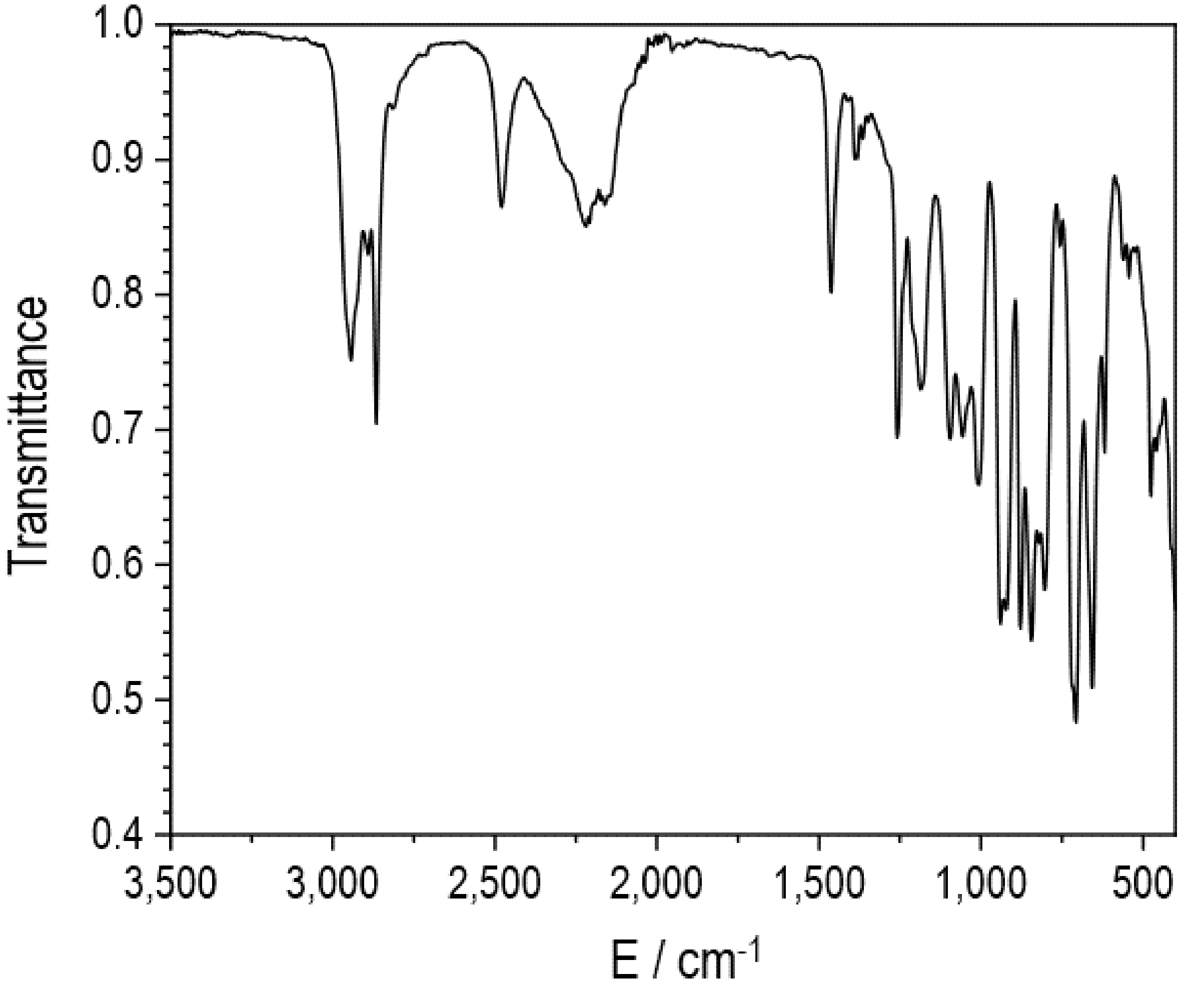

Figure S51. FT-IR (ATR, microcrystalline solid) spectrum of **2-Y** at ambient temperature.

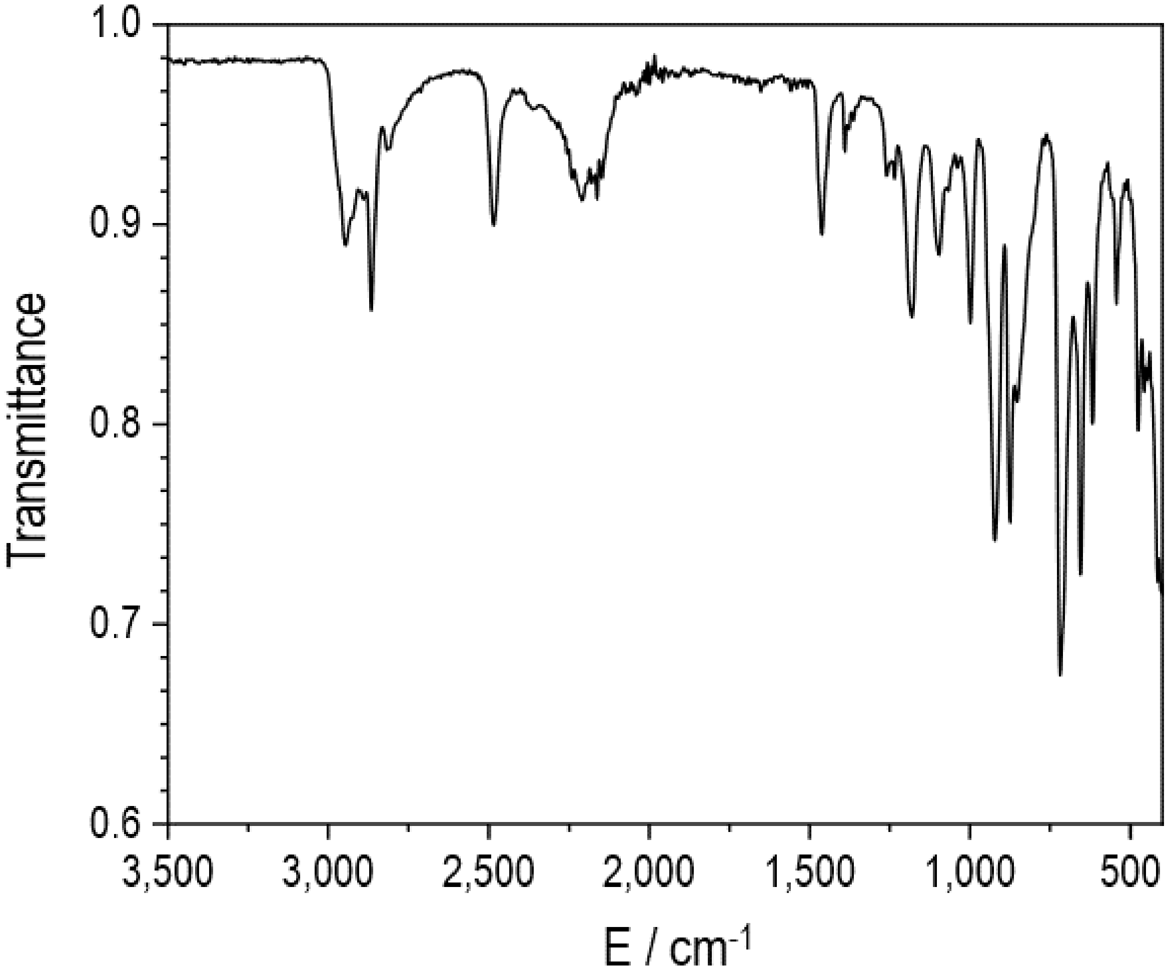

Figure S52. FT-IR (ATR, microcrystalline solid) spectrum of **2-Dy** at ambient temperature.

3.3 [Ln{N(Si^{i}Pr$_3$)$_2$}(BH$_4$)(μ-BH$_4$)]$_4$ (3-Ln)

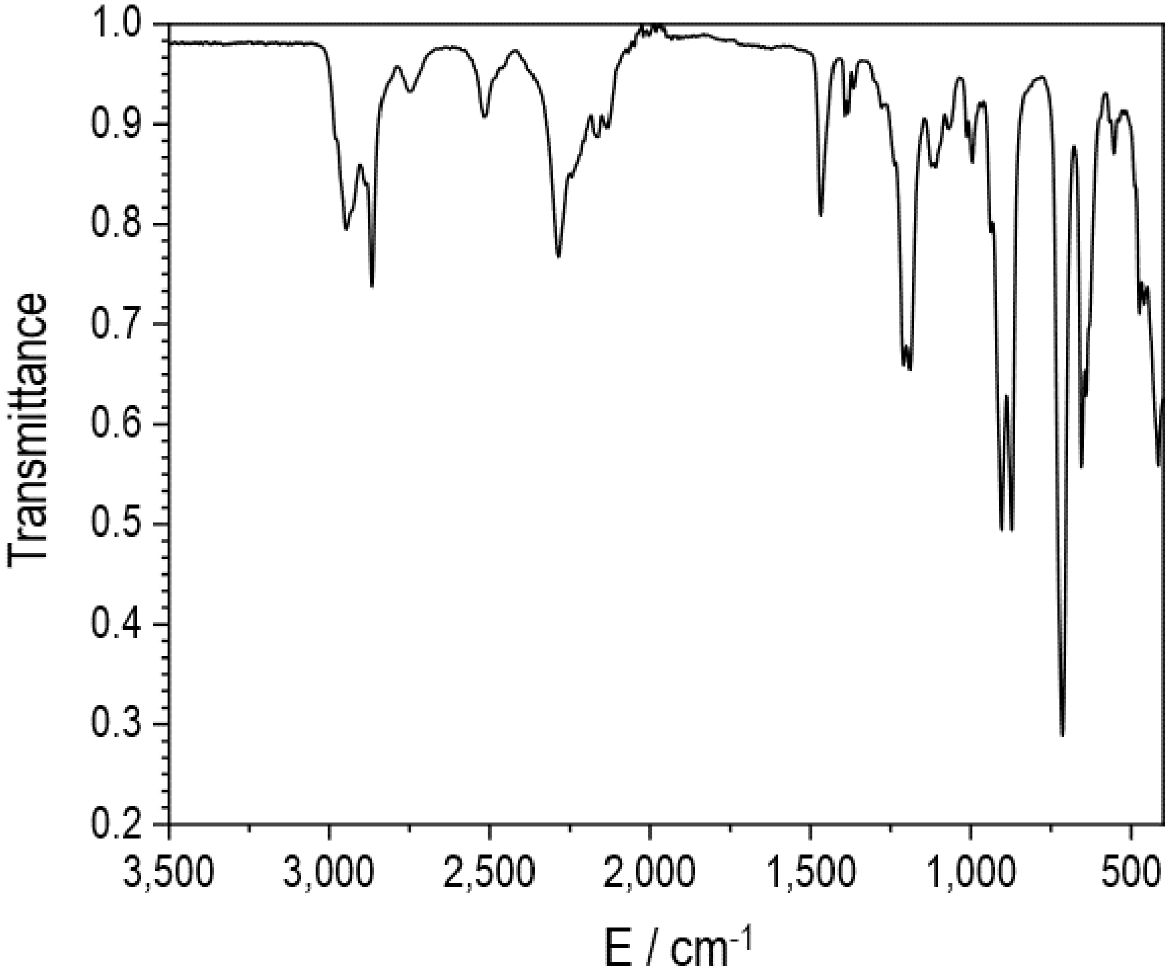

Figure S53. FT-IR (ATR, microcrystalline solid) spectrum of **3-Y** at ambient temperature.

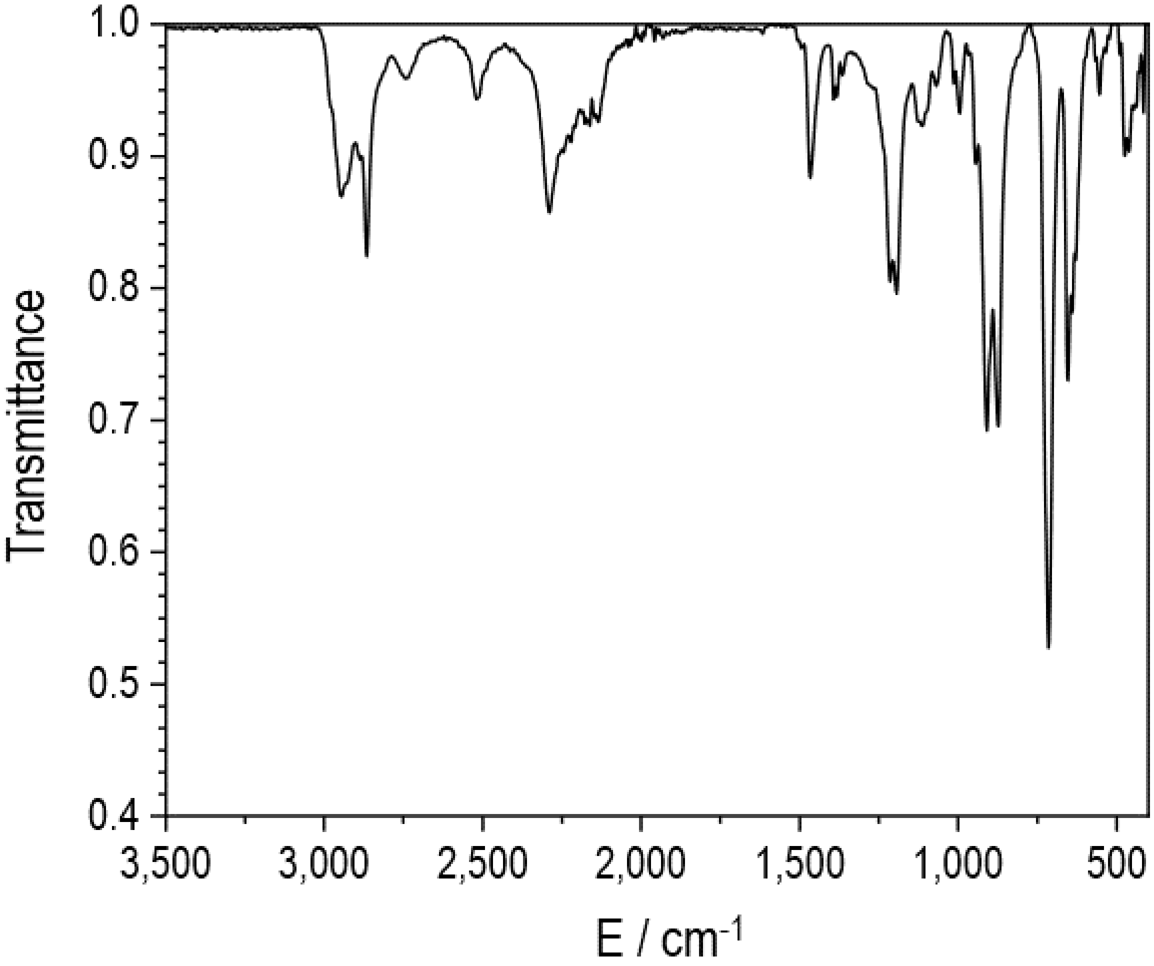

Figure S54. FT-IR (ATR, microcrystalline solid) spectrum of **3-Dy** at ambient temperature.

3.4 [Ln{N(Si^{i}Pr$_3$)$_2$}$_2$(BH$_4$)] (4-Ln)

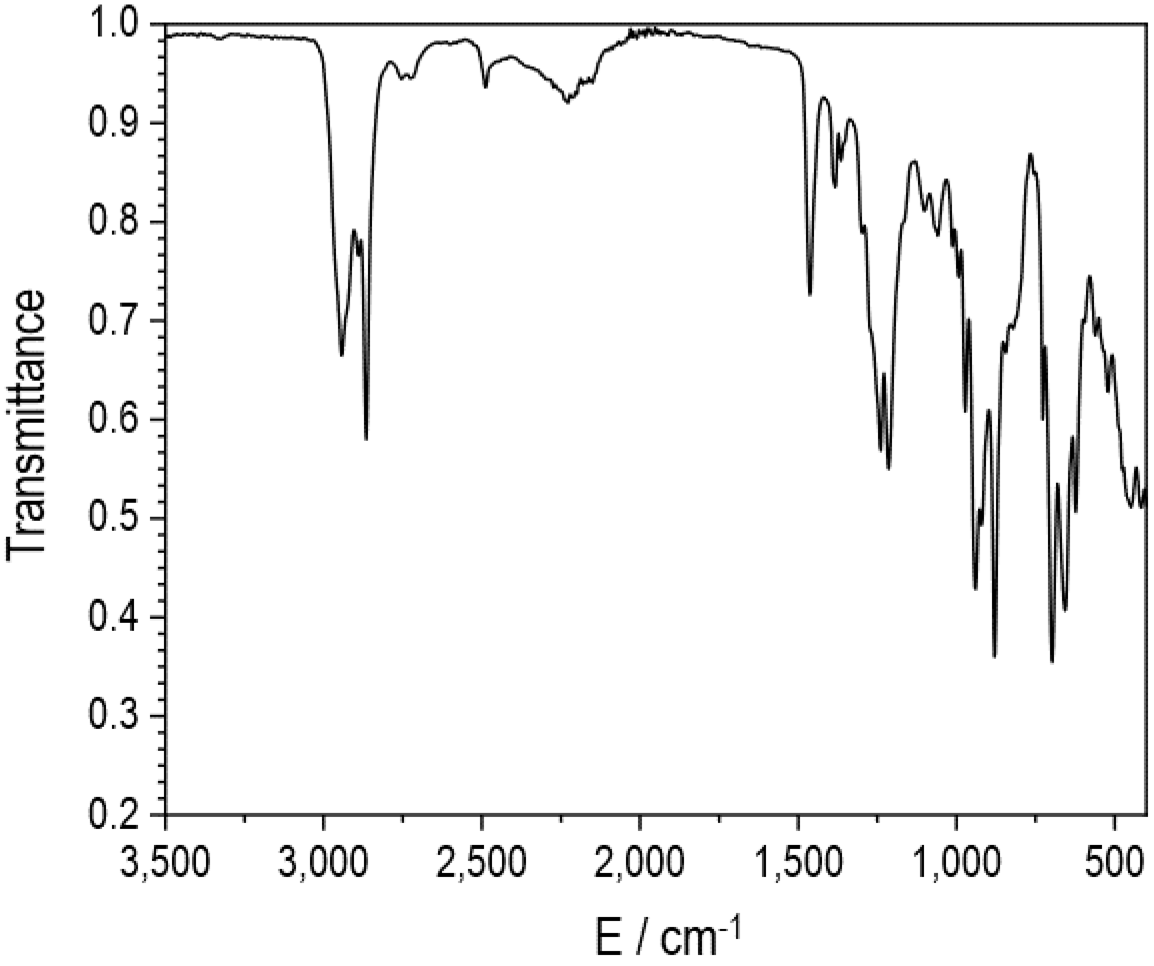

Figure S55. FT-IR (ATR, microcrystalline solid) spectrum of **4-Y** at ambient temperature.

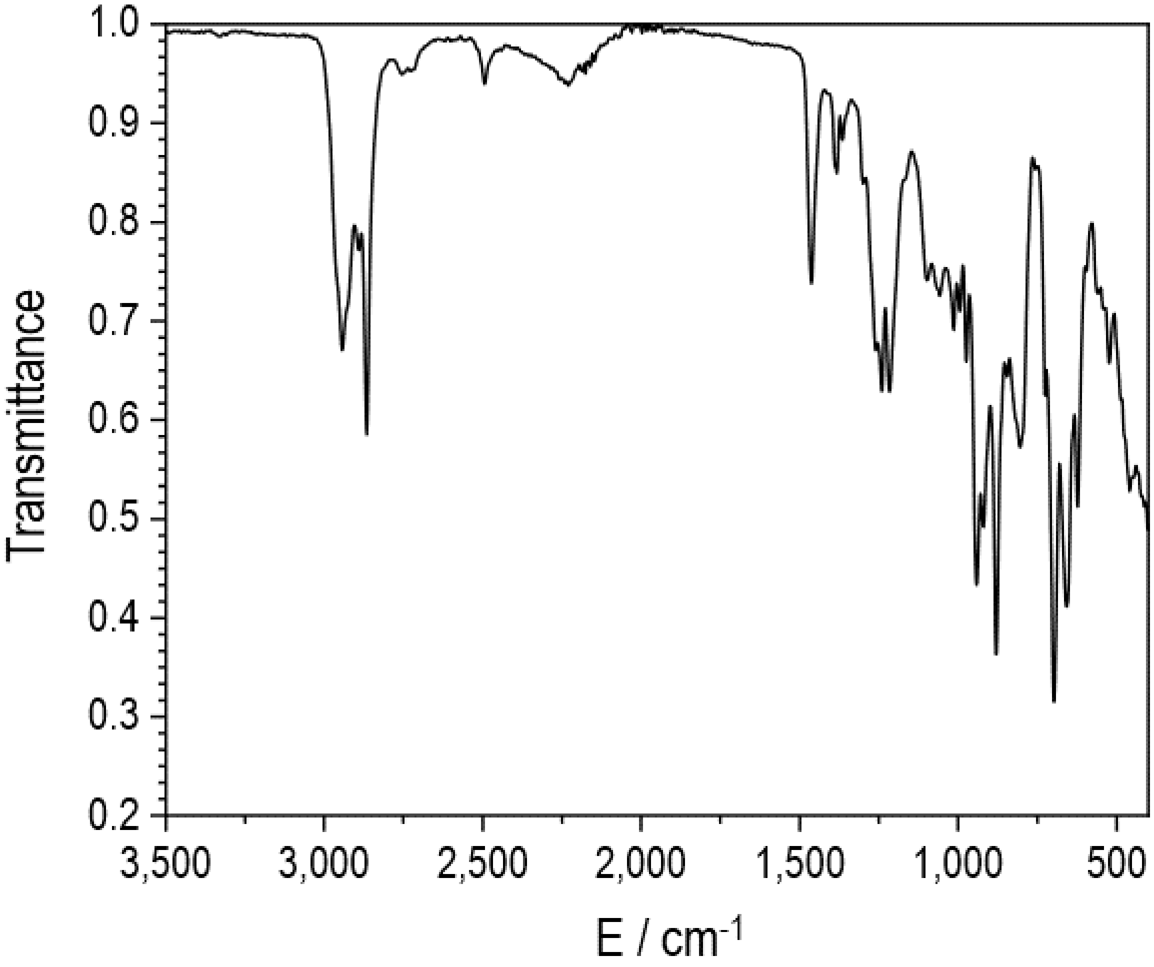

Figure S56. FT-IR (ATR, microcrystalline solid) spectrum of **4-Dy** at ambient temperature.

4. DFT calculated IR spectra

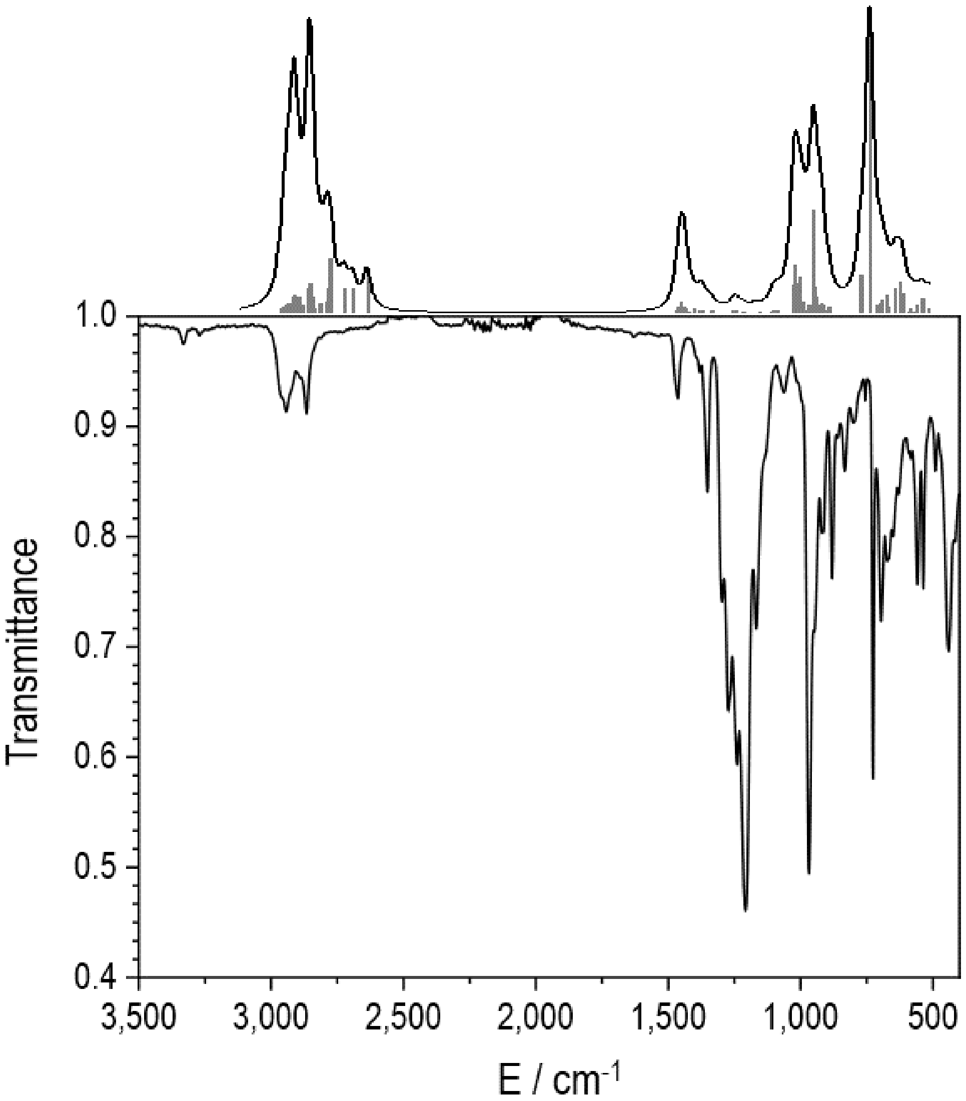

Figure S57. Comparison of the DFT-calculated IR spectrum of the cation of **1-Y** (top) with the experimentally derived spectra of **1-Y** at ambient temperature (bottom). A Lorentzian convolution (full width half maximum = 40 cm^{-1}) is applied to all calculated vibrational modes.

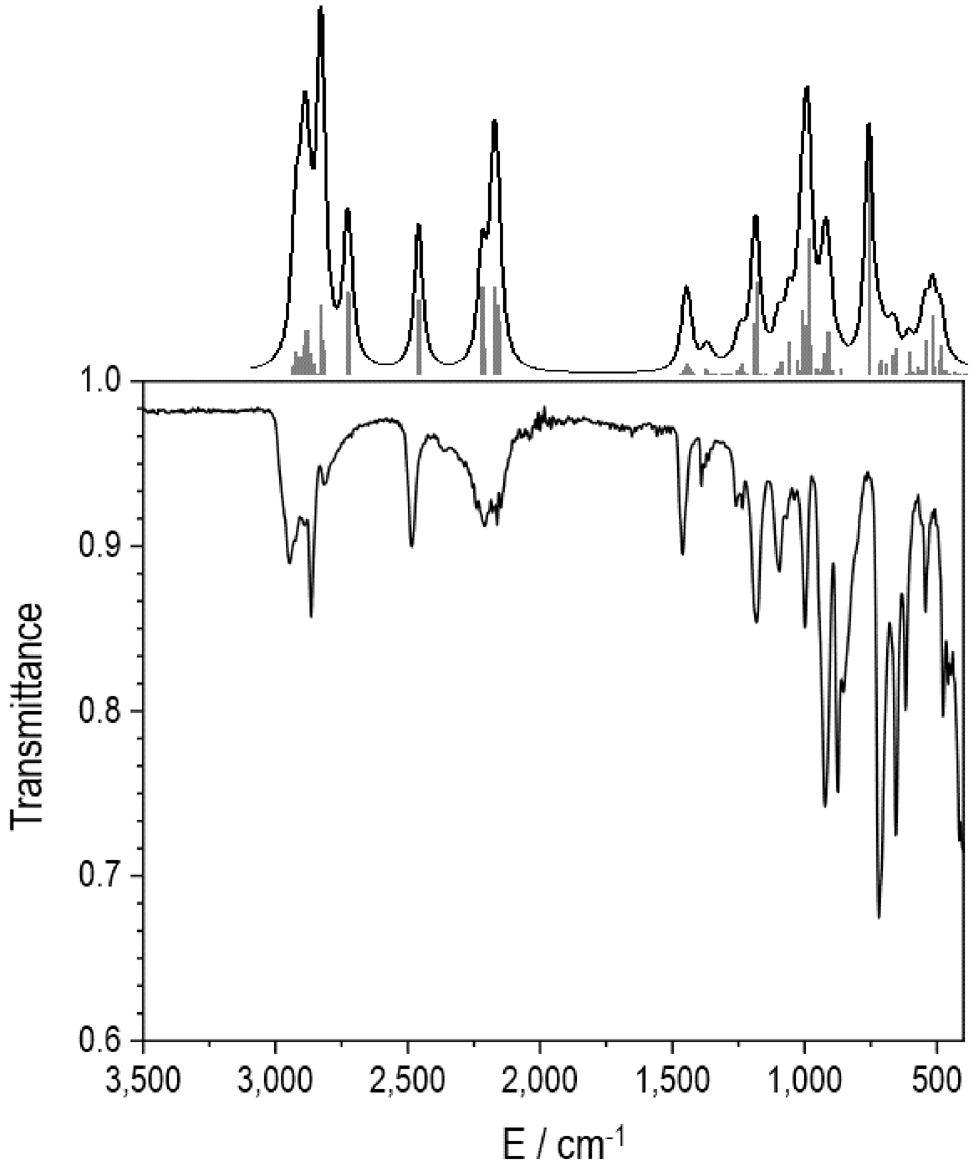

Figure S58. Comparison of the DFT-calculated IR spectrum of **2-Y** (top) with the experimentally derived spectra of **2-Y** at ambient temperature (bottom). A Lorentzian convolution (full width half maximum = 40 cm^{-1}) is applied to all calculated vibrational modes.

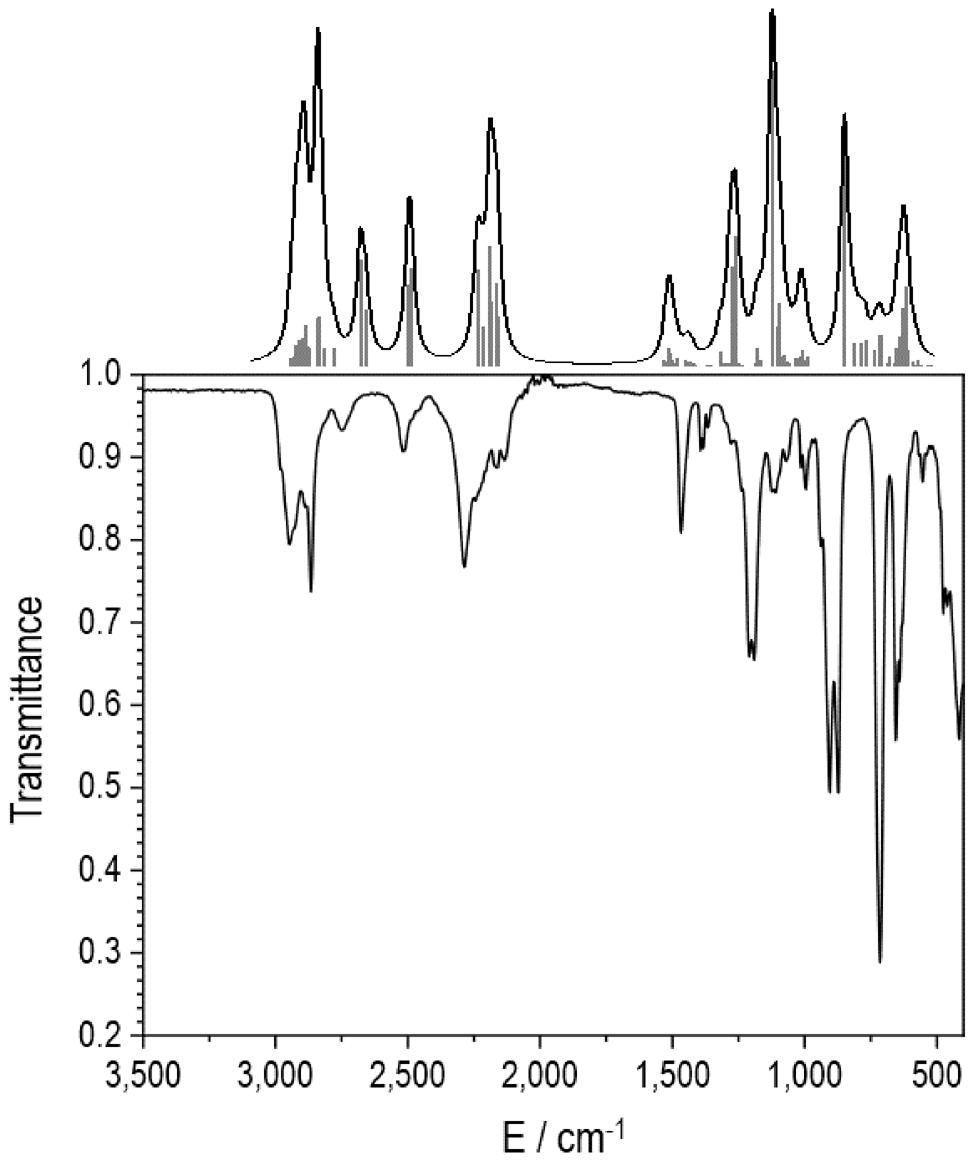

Figure S59. Comparison of the DFT-calculated IR spectrum of a monomer of **3-Y** (top) with the experimentally derived spectra of **3-Y** at ambient temperature (bottom). A Lorentzian convolution (full width half maximum = 40 cm^{-1}) is applied to all calculated vibrational modes.

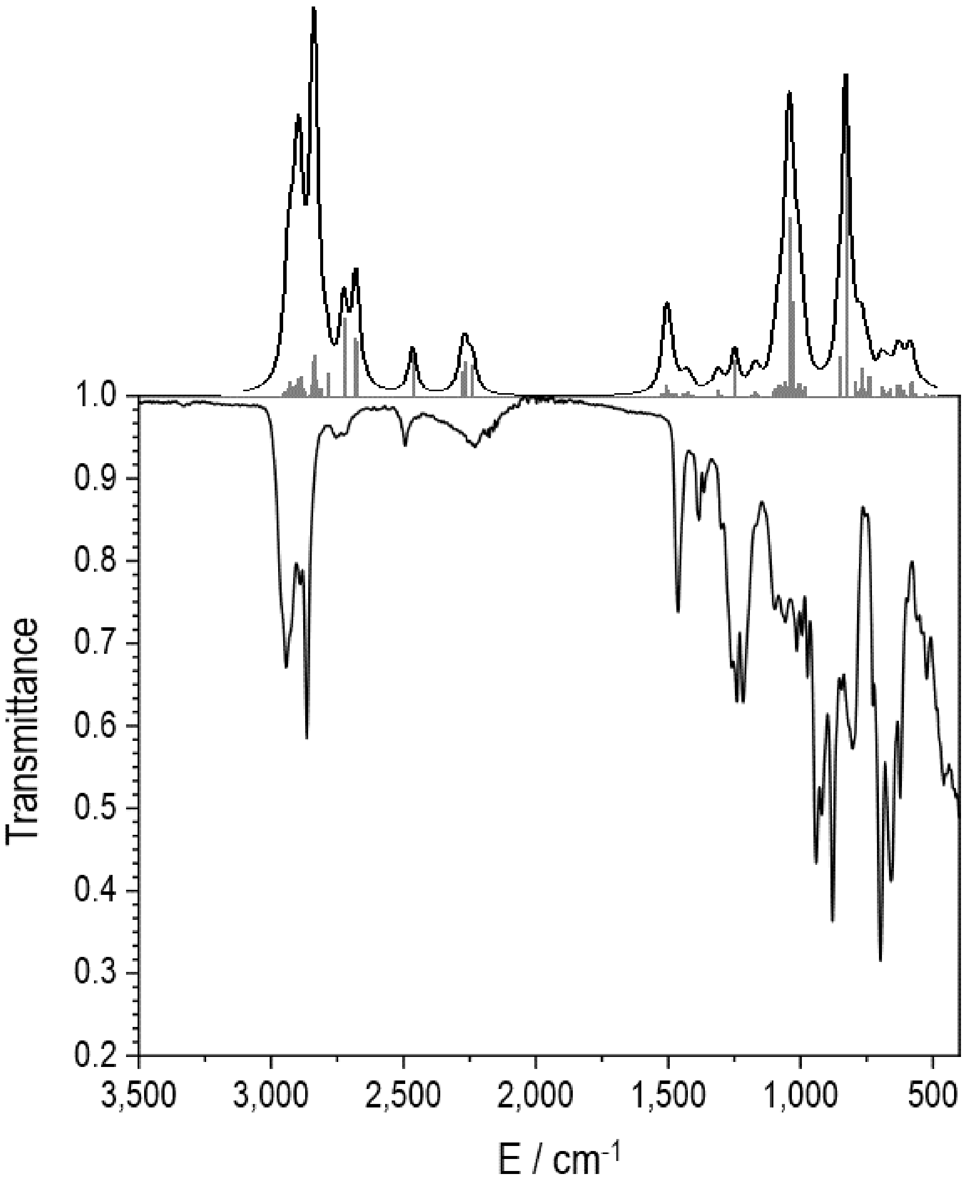

Figure S60. Comparison of the DFT-calculated IR spectrum of **4-Y** (top) with the experimentally derived spectra of **4-Y** at ambient temperature (bottom). A Lorentzian convolution (full width half maximum = 40 cm^{-1}) is applied to all calculated vibrational modes.

5. Single crystal X-ray diffraction

Table S1. Crystallographic data for **1-Ln** and **2-Ln**.

	**1-Y**	**1-Dy**	**2-Y**	**2-Dy**
Formula	$C_{52}H_{84}AlF_{36}N_2O_4Si_4Y$	$C_{52}H_{84}AlDyF_{36}N_2O_4S_{i4}$	$C_{22}H_{58}B_2NOSi_2Y$	$C_{22}H_{58}B_2DyNOSi_2$
Fw	1713.46	1787.05	519.40	592.99
Crystal size, mm	0.344 × 0.25 × 0.157	0.141 × 0.093 × 0.009	0.37 × 0.079 × 0.03	0.647 × 0.208 × 0.169
Crystal system	orthorhombic	orthorhombic	monoclinic	orthorhombic
Space group	*P*bca	*P*bca	$P2_1/c$	$P2_12_12_1$
Collection temperature, K	100(2)	100(2)	100(2)	100.00(10)
a, Å	19.4403(5)	19.4972(5)	8.8125(2)	13.0581(2)
b, Å	23.6130(9)	23.6772(8)	14.6779(3)	13.3893(2)
c, Å	31.6874(7)	31.7746(8)	23.0295(5)	16.8519(3)
α, °	90	90	90	90
β, °	90	90	92.139(2)	90
γ, °	90	90	90	90
V, Å^3	14545.9(8)	14668.4(7)	2976.77(11)	2946.37(8)
Z	8	8	4	4
ρ_{calcd}, g cm^{-3}	1.565	1.618	1.159	1.337
μ, mm^{-1}	1.014	7.418	3.603	2.630
No. of reflections made	76726	51591	18752	23774
No. of unique reflns, R_{int}	17384, 0.0982	9214, 0.0965	5985, 0.0323	6986, 0.0303
No. of reflns with $F^2 > 2\sigma(F^2)$	10292	7179	5517	6605
Transmn coeff range	0.08940-1.00000	0.462-1.000	0.737-1.000	0.648-1.000
R, R_w[a] ($F^2 > 2\sigma(F^2)$)	0.0579, 0.1020	0.0574, 0.1518	0.0339, 0.0990	0.0211, 0.0404
R, R_w[a] (all data)	0.1281, 0.1243	0.0743, 0.1647	0.0363, 0.1004	0.0239, 0.0417
S[a]	1.012	0.985	1.116	1.047
Parameters, Restraints	1289, 5001	1043, 1624	310, 0	317, 49
Max., min. diff map, e Å^{-3}	0.762, –0.499	1.143, –1.806	0.550, –1.365	0.557, –0.431

[a] Conventional $R = \Sigma||F_o| - |F_c||/\Sigma|F_o|$; $R_w = [\Sigma w(F_o^2 - F_c^2)^2/\Sigma w(F_o^2)^2]^{1/2}$; $S = [\Sigma w(F_o^2 - F_c^2)^2/\text{no. data} - \text{no. params})]^{1/2}$ for all data.

Table S2. Crystallographic data for **3-Ln** and **3-Ln·0.5KBH$_4$**.

	**3-Y**	**3-Dy**	**3-Y·0.5KBH$_4$·C$_6$H$_6$**	**3-Dy·0.5KBH$_4$·1,2-C$_6$H$_4$F$_2$**
Formula	$C_{72}H_{200}B_8N_4Si_8Y_4$	$C_{72}H_{200}B_8Dy_4N_4Si_8$	$C_{39}H_{107}B_5KN_2Si_4Y_2$	$C_{39}H_{105}B_5Dy_2FKN_2Si_4$
Fw	1789.19	2083.55	987.59	1151.75
Crystal size, mm	0.118 × 0.088 × 0.029	0.048 × 0.029 × 0.018	0.195 × 0.087 × 0.041	0.227 × 0.113 × 0.102
Crystal system	monoclinic	monoclinic	triclinic	Triclinic
Space group	$P2_1$/c	$P2_1$/c	*P*-1	*P*-1
Collection temperature, K	100(2)	100.01(15)	100(2)	100(2)
a, Å	18.0513(2)	17.9951(16)	8.56937(5)	8.5475(2)
b, Å	28.9661(3)	28.790(3)	18.02045(9)	18.0650(5)
c, Å	21.0674(2)	20.982(3)	18.50778(7)	18.4576(7)
α, °	90	90	89.5896(4)	91.009(3)
β, °	109.8967(13)	109.999(13)	82.7373(4)	98.027(3)
γ, °	90	90	86.5114(4)	93.085(2)
V, Å^3	10358.1(2)	10214(2)	2829.86(2)	2817.12(15)
Z	4	4	2	2
ρ_{calcd}, g cm^{-3}	1.147	1.355	1.159	1.358
μ, mm^{-1}	4.039	16.533	4.383	2.820
No. of reflections made	136906	16460	46757	24819
No. of unique reflns, R_{int}	21445, 0.0558	8302, 0.0972	11540, 0.0251	12813, 0.0347
No. of reflns with $F^2 > 2\sigma(F^2)$	18368	4715	10918	9853
Transmn coeff range	0.887-1.000	0.04783-1.00000	0.608-1.000	0.622-1.000
R, R_w[a] ($F^2 > 2\sigma(F^2)$)	0.0520, 0.1375	0.0738, 0.1793	0.0234, 0.0615	0.0338, 0.0603
R, R_w[a] (all data)	0.0590, 0.1419	0.1453, 0.2084	0.0248, 0.0622	0.0557, 0.0674
S[a]	1.078	0.898	1.075	0.983
Parameters, Restraints	1119, 412	961, 1616	682, 363	699, 1049
Max., min. diff map, e Å^{-3}	0.935, –1.908	1.728, –0.722	0.403, –0.474	1.427, –0.954

[a] Conventional $R = \Sigma||F_o| - |F_c||/\Sigma|F_o|$; $R_w = [\Sigma w(F_o^2 - F_c^2)^2/\Sigma w(F_o^2)^2]^{1/2}$; $S = [\Sigma w(F_o^2 - F_c^2)^2$/no. data – no. params)$]^{1/2}$ for all data.

Table S3. Crystallographic data for **4-Ln**.

	**4-Y**[b]	**4-Y**[c]	**4-Dy**
Formula	$C_{36}H_{88}BN_2Si_4Y$	$C_{36}H_{88}BN_2Si_4Y$	$C_{36}H_{88}BDyN_2Si_4$
Fw	761.16	761.16	834.75
Crystal size, mm	0.205 × 0.184 × 0.103	0.086 × 0.071 × 0.022	0.171 × 0.127 × 0.054
Crystal system	monoclinic	orthorhombic	orthorhombic
Space group	$P2_1/c$	*P*bca	*P*bca
Collection temperature, K	100(2)	100(2)	100(2)
a, Å	15.9332(13)	20.3776(3)	20.3179(5)
b, Å	13.1915(8)	20.0917(4)	19.9900(5)
c, Å	22.5401(14)	21.6614(4)	21.7278(4)
α, °	90	90	90
β, °	108.911(8)	90	90
γ, °	90	90	90
V, Å^3	4481.8(6)	8868.6(3)	8824.9(3)
Z	4	8	8
ρ_{calcd}, g cm^{-3}	1.128	1.140	1.257
μ, mm^{-1}	1.433	3.055	10.268
No. of reflections made	22250	47249	51634
No. of unique reflns, R_{int}	9146, 0.0578	9116, 0.0566	7995, 0.0790
No. of reflns with $F^2 > 2\sigma(F^2)$	6307	7675	6169
Transmn coeff range	0.719-1.000	0.912-1.000	0.69935-1.00000
R, R_w[a] ($F^2 > 2\sigma(F^2)$)	0.0496, 0.0845	0.0389, 0.0997	0.0474, 0.1084
R, R_w[a] (all data)	0.0928, 0.0974	0.0473, 0.1041	0.0677, 0.1169
S[a]	1.004	0.999	1.031
Parameters, Restraints	473, 164	516, 1314	492, 329
Max., min. diff map, e Å^{-3}	0.442, –0.434	0.591, –1.147	2.149, –1.182

[a] Conventional $R = \Sigma||F_o| - |F_c||/\Sigma|F_o|$; $R_w = [\Sigma w(F_o^2 - F_c^2)^2/\Sigma w(F_o^2)^2]^{1/2}$; $S = [\Sigma w(F_o^2 - F_c^2)^2/\text{no. data} - \text{no. params})]^{1/2}$ for all data. [b] Polymorph in $P2_1/c$. [c] Polymorph in *P*bca.

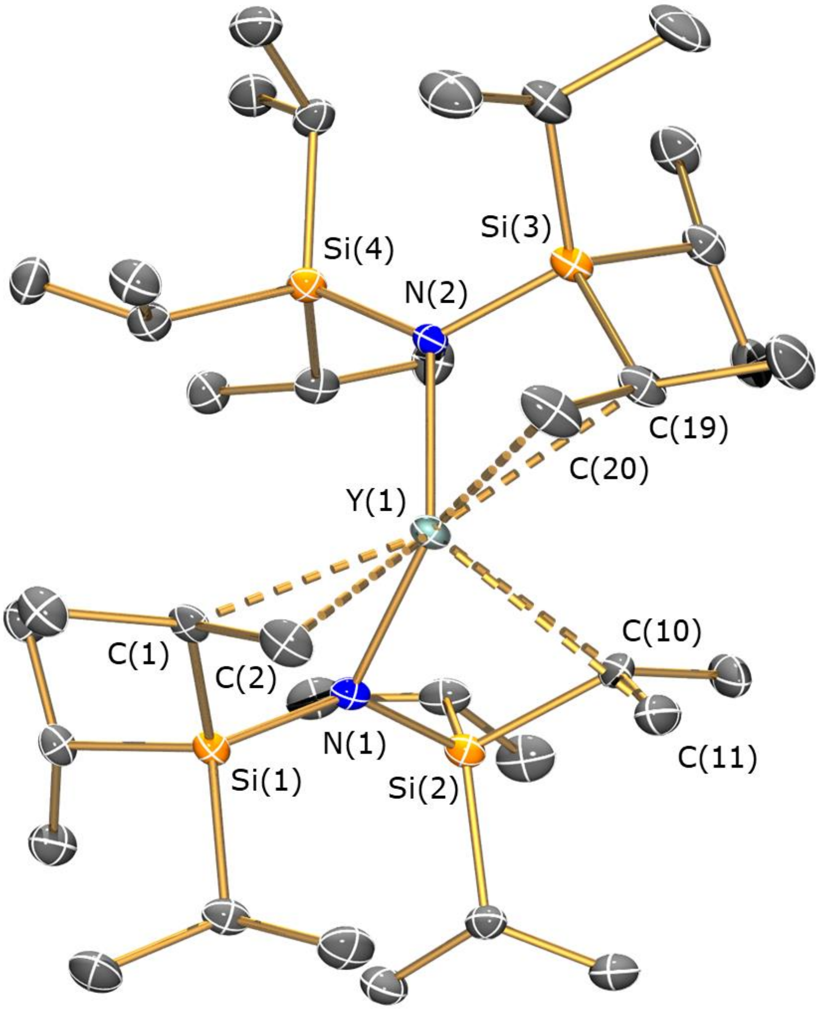

Figure S61. Solid-state crystal structure of the cation of **1-Y** at 100(2) K. Displacement ellipsoids set at 50% probability level. Hydrogen atoms and $[Al\{OC(CF_3)_3\}_4]^-$ counter-anion omitted for clarity. Selected bond distances (Å) and angles (°): Y(1)–N(1): 2.194(2); Y(1)–N(2): 2.182(3); Y(1)···C(1): 2.838(3); Y(1)···C(2): 2.962(3); Y(1)···C(10): 2.852(4); Y(1)···C(11): 3.010(4); Y(1)···C(19): 2.826(3); Y(1)···C(20): 2.872(3); Y(1)···Si(1): 3.2023(9); Y(1)···Si(2): 3.2108(11); Y(1)···Si(3): 3.1822(9); N(1)–Y(1)–N(2): 126.70(9).

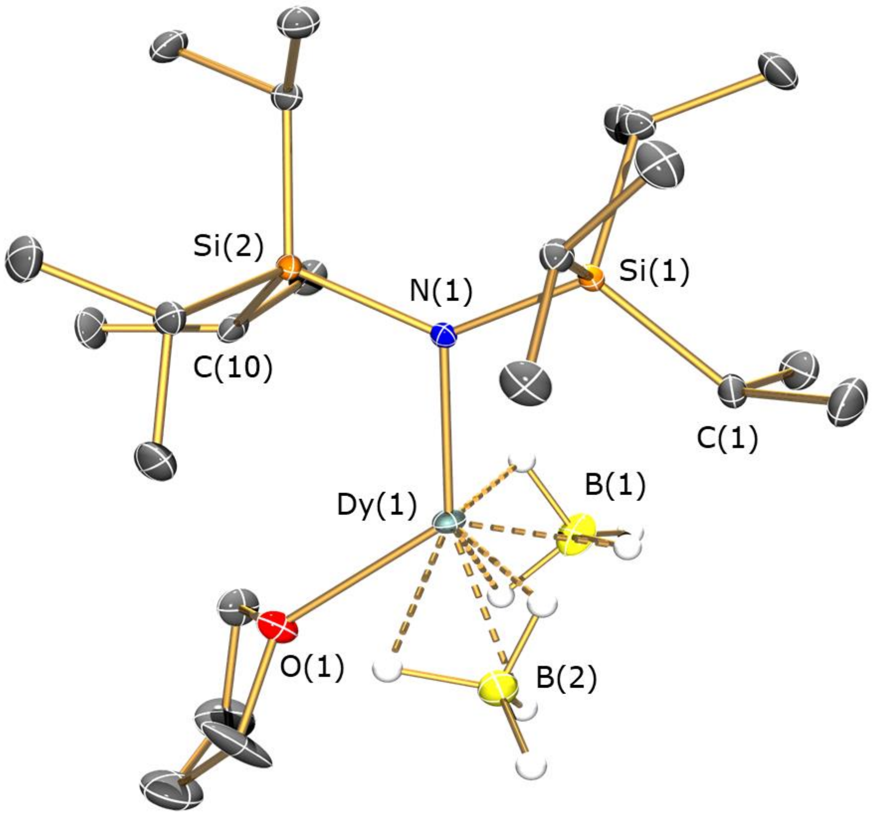

Figure S62. Solid-state crystal structure of [Dy{N(Si^{i}Pr$_3$)$_2$}(BH$_4$)$_2$(THF)] (**2-Dy**) at 100(2) K. Displacement ellipsoids set at 50% probability level. Non-borohydride hydrogen atoms omitted for clarity. Selected bond distances (Å) and angles (°): Dy(1)–N(1): 2.211(2); Dy(1)···B(1): 2.498(5); Dy(1)···B(2): 2.516(5); Dy(1)–O(1): 2.367(2); Dy(1)···C(1): 3.370(3); Dy(1)···C(10): 3.341(3); Dy(1)···Si(1): 3.3768(8); Dy(1)···Si(2): 3.3404(8); N(1)–Dy(1)···B(1): 112.00(16); N(1)–Dy(1)···B(2): 115.64(16); N(1)–Dy(1)–O(1): 127.22(8); B(1)···Dy(1)···B(2): 113.82(14); B(1)···Dy(1)–O(1): 93.68(15); B(2)···Dy(1)–O(1): 91.85(15).

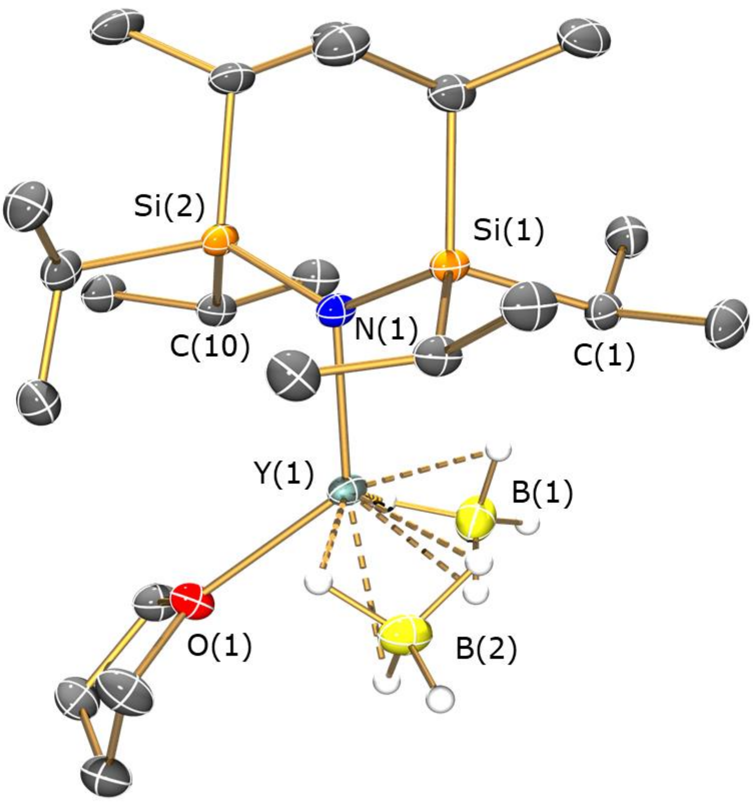

Figure S63. Solid-state crystal structure of [Y{N(Si^{i}Pr$_3$)$_2$}(BH$_4$)$_2$(THF)] (**2-Y**) at 100(2) K. Displacement ellipsoids set at 50% probability level. Non-borohydride hydrogen atoms and minor disordered THF component omitted for clarity. Selected bond distances (Å) and angles (°): Y(1)–N(1): 2.254(2); Y(1)···B(1): 2.484(3); Y(1)···B(2): 2.484(3); Y(1)–O(1): 2.3449(13); Y(1)···C(1): 3.6506(19); Y(1)···C(10): 3.1241(19); Y(1)···Si(1): 3.4356(5); Y(1)···Si(2): 3.2404(5); N(1)–Y(1)···B(1): 118.95(8); N(1)–Y(1)···B(2): 107.89(7); N(1)–Y(1)–O(1): 128.21(6); B(1)···Y(1)···B(2): 109.13(9); B(1)···Y(1)–O(1): 95.31(8); B(2)···Y(1)–O(1): 94.02(7).

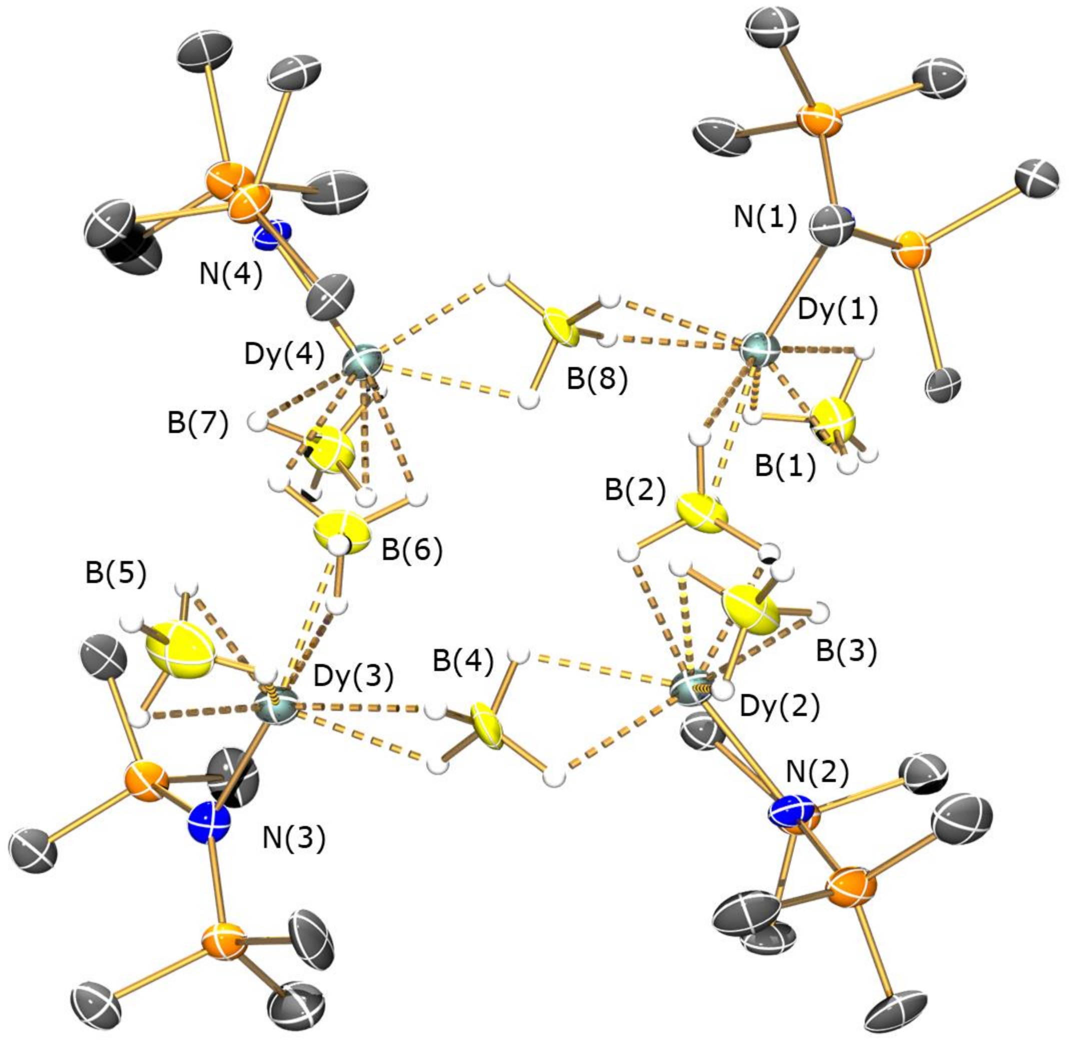

Figure S64. Solid-state crystal structure of $[Dy\{N(Si^iPr_3)_2\}(BH_4)_2]_4$ (**3-Dy**) at 100(2) K. Displacement ellipsoids set at 30% probability level. Non-borohydride hydrogen atoms and ^{i}Pr CH_3 carbon atoms omitted for clarity. Selected bond distances (Å) and angles (°): Dy(1)–N(1): 2.187(11); Dy(1)···B(1): 2.41(2); Dy(1)···B(2): 2.72(2); Dy(1)···B(8): 2.77(2); N(1)–Dy(1)···B(1): 116.6(6); N(1)–Dy(1)···B(2): 112.0(5); N(1)–Dy(1)···B(8): 126.0(5); B(1)···Dy(1)···B(2): 119.1(7); B(1)···Dy(1)···B(8): 99.1(8); B(2)···Dy(1)···B(8): 79.1(6).

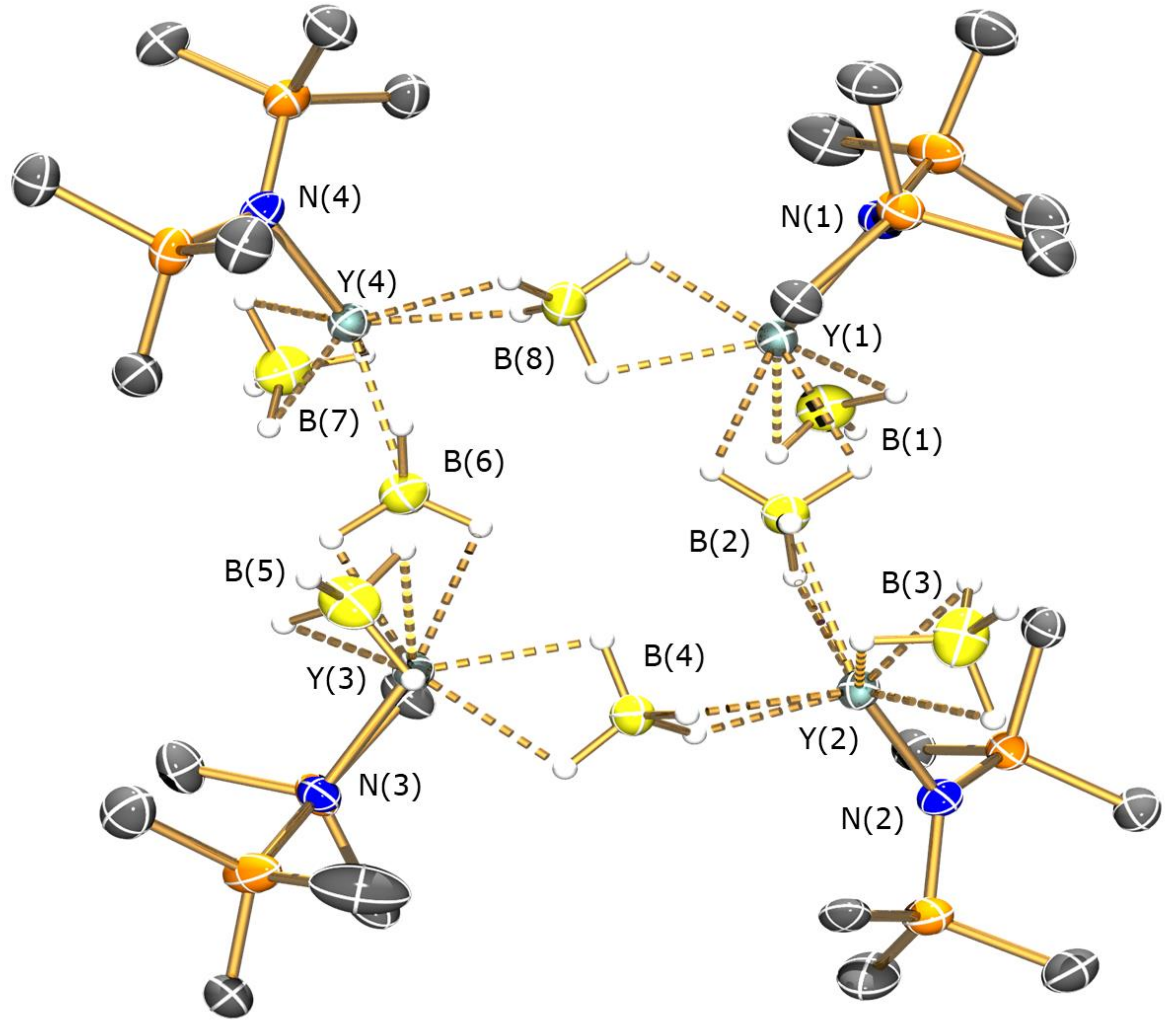

Figure S65. Solid-state crystal structure of [Y{N(Si^{i}Pr$_3$)$_2$}(BH$_4$)$_2$]$_4$ (**3-Y**) at 100(2) K. Displacement ellipsoids set at 50% probability level. Non-borohydride hydrogen atoms and ^{i}Pr CH$_3$ carbon atoms omitted for clarity. Selected bond distances (Å) and angles (°): Y(1)–N(1): 2.183(3); Y(1)···B(1): 2.458(6); Y(1)···B(2): 2.785(5); Y(1)···B(8): 2.737(4); N(1)–Y(1)···B(1): 116.1(2); N(1)–Y(1)···B(2): 126.44(11); N(1)–Y(1)···B(8): 112.04(12); B(1)···Y(1)···B(2): 99.3(2); B(1)···Y(1)···B(8): 119.2(2); B(2)···Y(1)···B(8): 79.02(11).

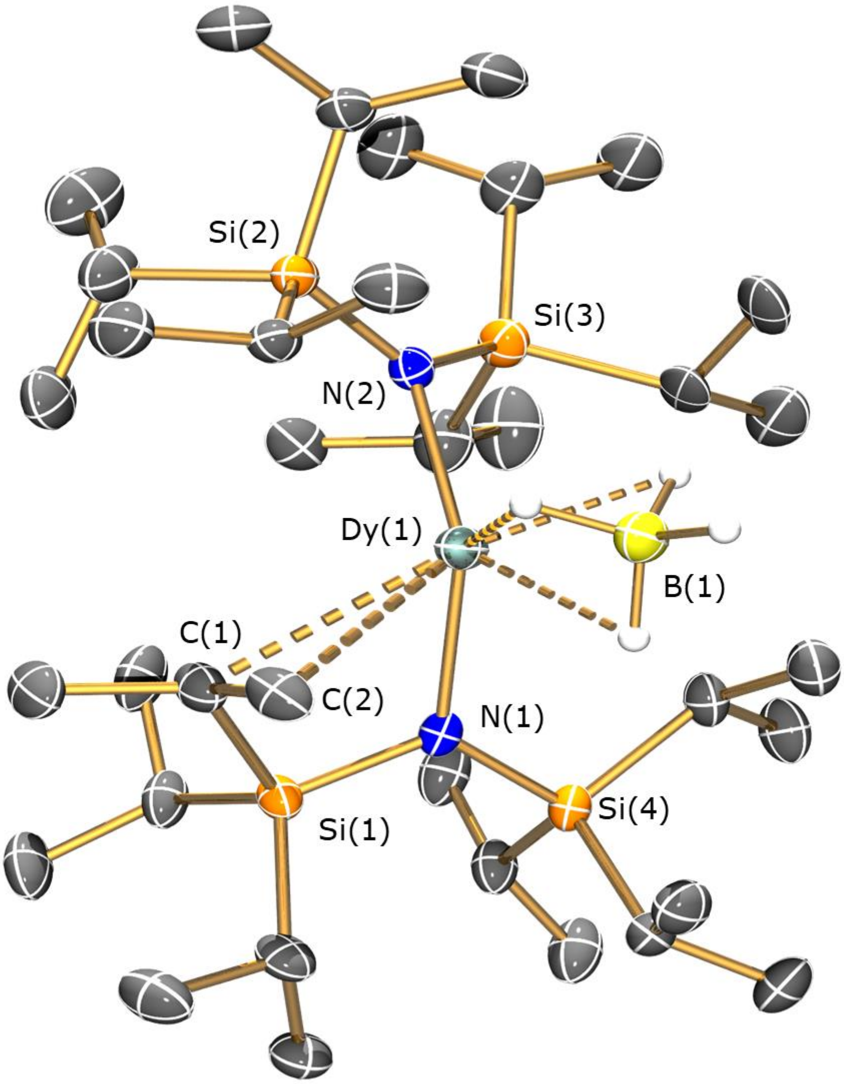

Figure S66. Solid-state crystal structure of [Dy{N(Si^{i}Pr$_3$)$_2$}$_2$(BH$_4$)] (**4-Dy**) at 100(2) K. Displacement ellipsoids set at 50% probability level. Non-borohydride hydrogen atoms and minor ^{i}Pr disordered components omitted for clarity. Selected bond distances (Å) and angles (°): Dy(1)–N(1): 2.266(4); Dy(1)–N(2): 2.288(4); Dy(1)···B(1): 2.483(6); Dy(1)–C(1): 2.989(4); Dy(1)–C(1): 3.128(4); Dy(1)–Si(1): 3.3152(11); Dy(1)–Si(2): 3.4092(12); Dy(1)–Si(3): 3.4072(13); Dy(1)–Si(4): 3.5734(11); N(1)–Dy(1)–N(2): 130.41(13); N(1)–Dy(1)···B(1): 112.40(15); N(2)–Dy(1)···B(1): 113.93(15).

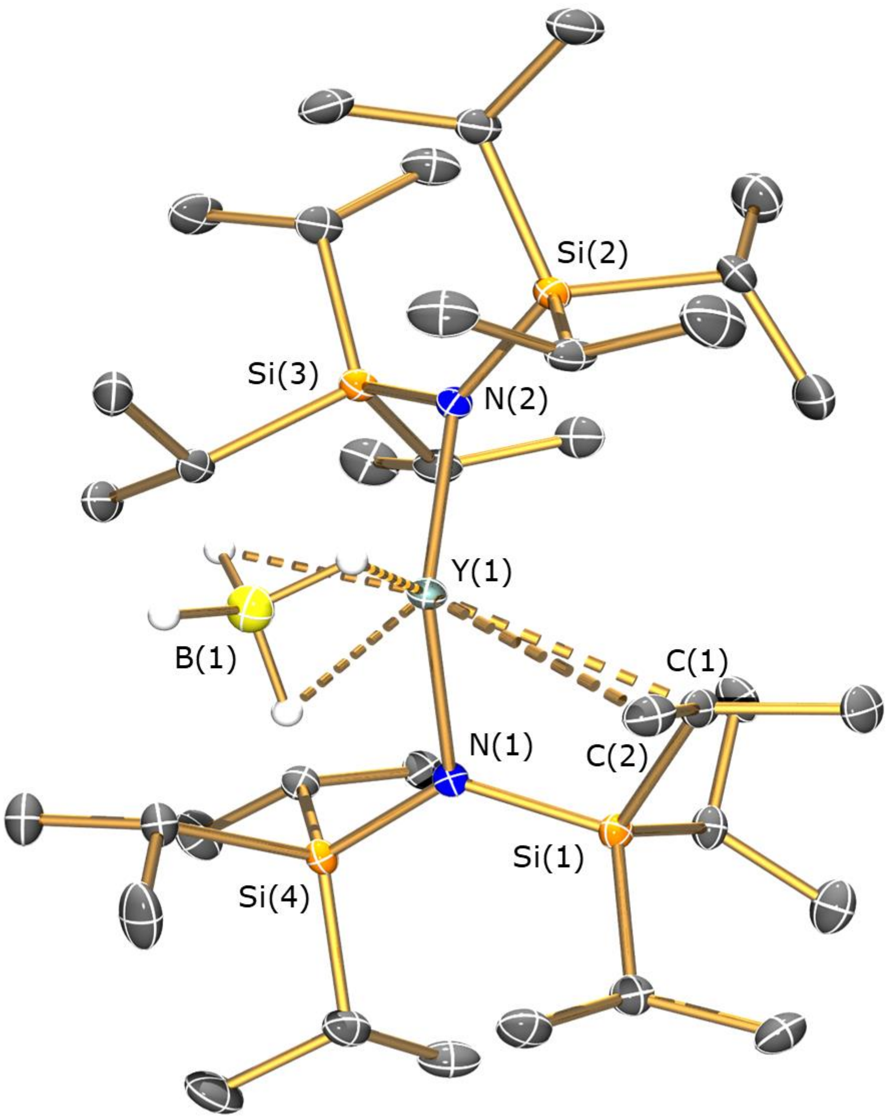

Figure S67. Solid-state crystal structure of the $P2_1$/c polymorph of $[Y\{N(Si^iPr_3)_2\}_2(BH_4)]$ (**4-Y**) at 100(2) K. Displacement ellipsoids set at 50% probability level. Non-borohydride hydrogen atoms and minor ^{i}Pr disordered components omitted for clarity. Selected bond distances (Å) and angles (°): Y(1)–N(1): 2.251(2); Y(1)–N(2): 2.283(2); Y(1)···B(1): 2.483(5); Y(1)–C(1): 2.907(3); Y(1)–C(2): 3.078(3); Y(1)–Si(1): 3.2238(10); Y(1)–Si(2): 3.4025(9); Y(1)–Si(3): 3.4013(10); Y(1)–Si(4): 3.5618(9); N(1)–Y(1)–N(2): 127.36(8); N(1)–Y(1)···B(1): 110.02(11); N(2)–Y(1)···B(1): 116.58(11).

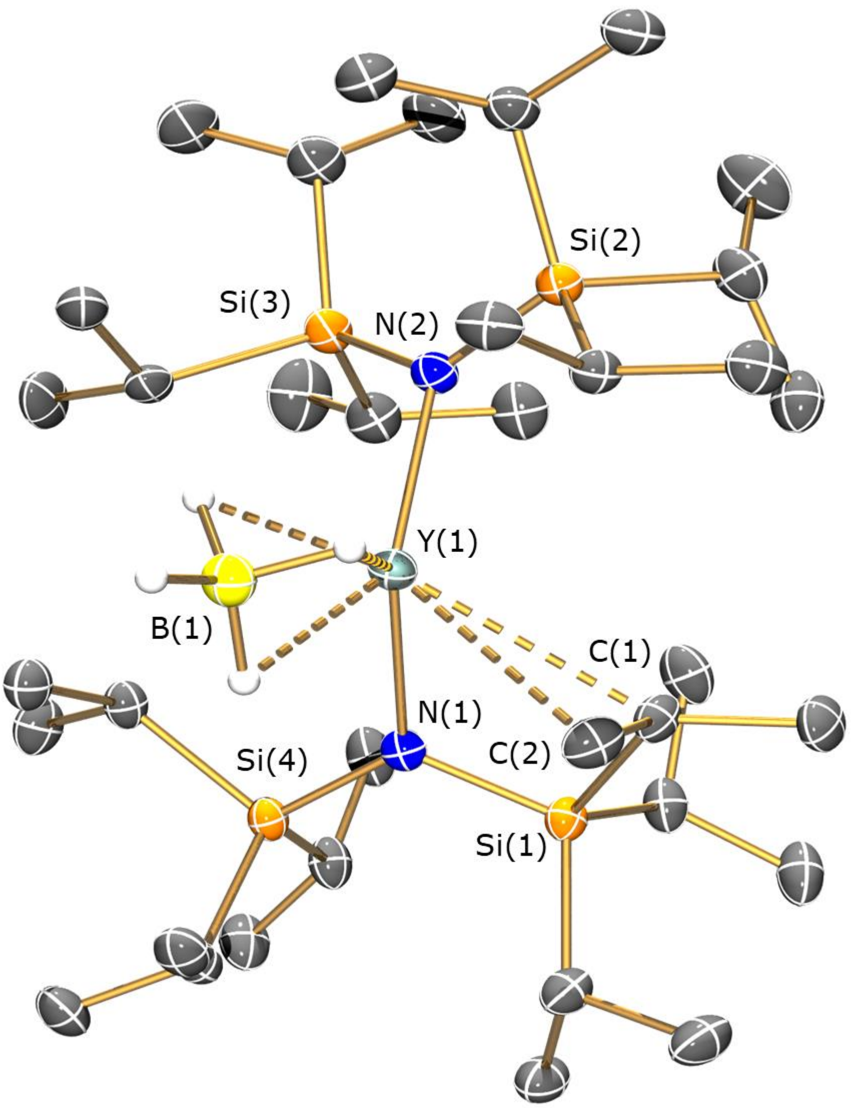

Figure S68. Solid-state crystal structure of the *P*bca polymorph of [Y{N(Si^{i}Pr$_3$)$_2$}$_2$(BH$_4$)] (**4-Y**) at 100(2) K. Displacement ellipsoids set at 50% probability level. Non-borohydride hydrogen atoms and minor ^{i}Pr disordered components omitted for clarity. Selected bond distances (Å) and angles (°): Y(1)–N(1): 2.288(2); Y(1)–N(2): 2.268(2); Y(1)···B(1): 2.493(3); Y(1)–C(1): 2.968(2); Y(1)–C(2): 3.104(2); Y(1)–Si(1): 3.3109(7); Y(1)–Si(2): 3.5865(7); Y(1)–Si(3): 3.4046(7); Y(1)–Si(4): 3.4206(6); N(1)–Y(1)–N(2): 128.20(7); N(1)–Y(1)···B(1): 113.15(8); N(2)–Y(1)···B(1): 114.72(8).

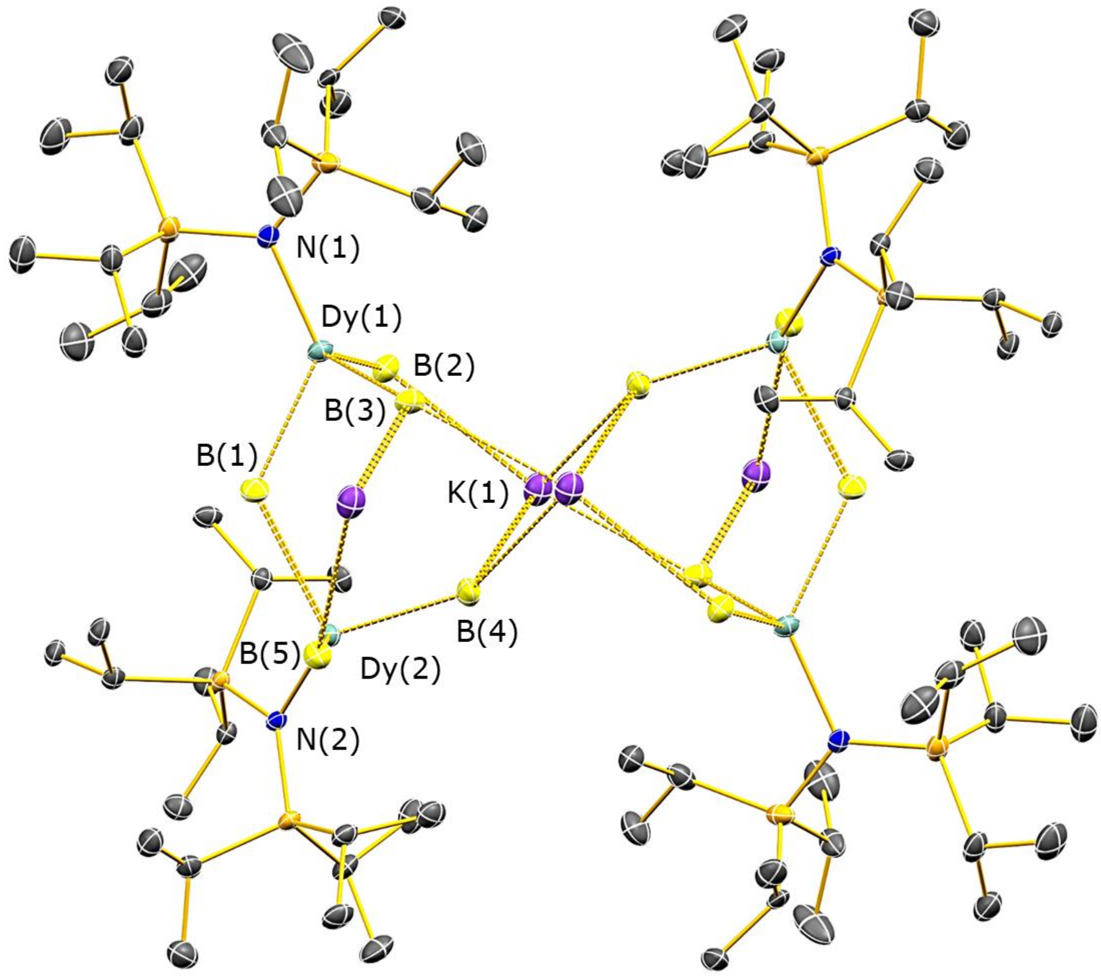

Figure S69. Solid-state crystal structure of a dimeric unit of polymeric $[\{Dy\{N(Si^{i}Pr_3)_2\}(BH_4)(\mu\text{-}BH_4)\}_2\{K(\mu\text{-}BH_4)\}]_\infty\cdot 1,2\text{-}C_6H_4F_2$ (**3-Dy·0.5KBH4·1,2-C6H4F2**) at 100(2) K, viewed along the principal axis of the extended 1D coordination polymer. Displacement ellipsoids set at 50% probability level. All hydrogen atoms and co-crystallized 1,2-$C_6H_4F_2$ omitted for clarity. Selected bond distances (Å): Dy(1)–N(1): 2.185(3); Dy(2)–N(2): 2.185(3); Dy(1)···B(1): 2.656(5); Dy(1)···B(2): 2.739(5); Dy(1)···B(3): 2.516(5); Dy(2)···B(1): 2.842(5); Dy(2)···B(4): 2.509(5); Dy(2)···B(5): 2.558(5); K(1)···B(2): 3.252(5); K(1)···B(4): 3.576(5).

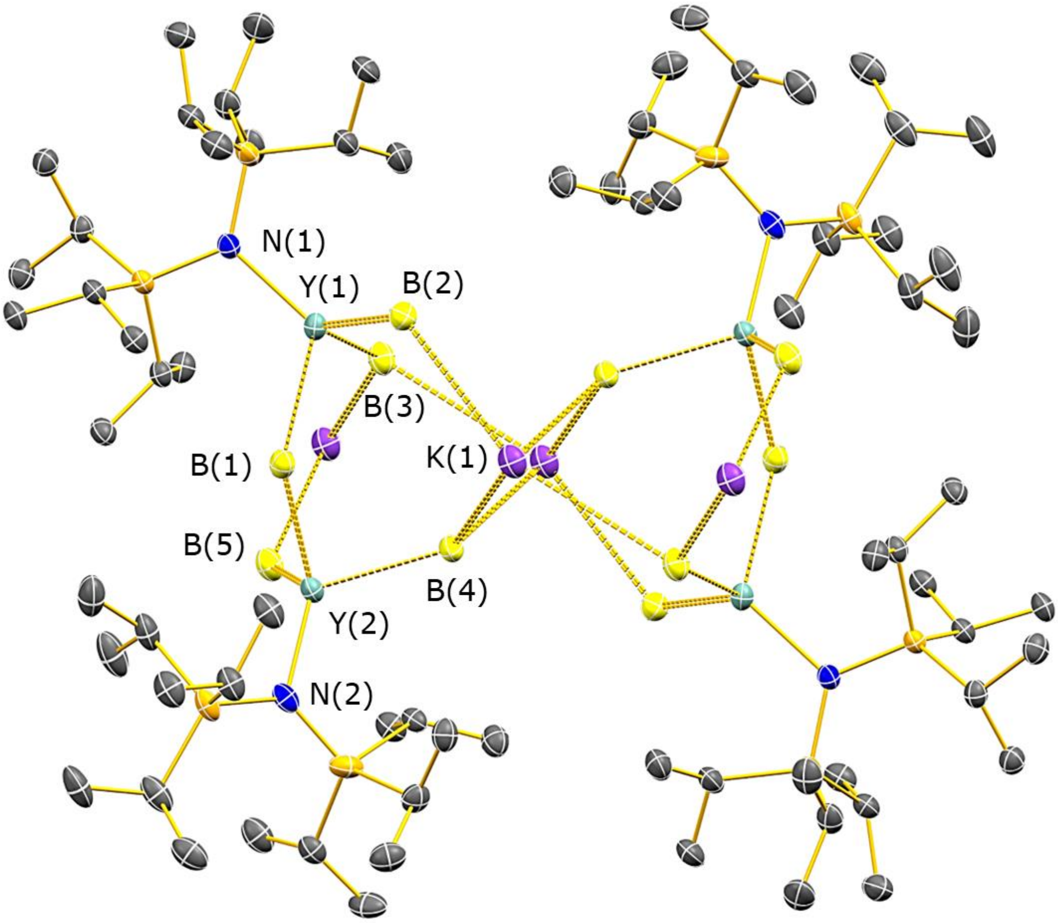

Figure S70. Solid-state crystal structure of a dimeric unit of polymeric $[\{Y\{N(Si^{i}Pr_3)_2\}(BH_4)(\mu\text{-}BH_4)\}_2\{K(\mu\text{-}BH_4)\}]_\infty\cdot C_6H_6$ (**3-Y·0.5KBH$_4$·C$_6$H$_6$**) at 100(2) K, viewed along the principal axis of the extended 1D coordination polymer. Displacement ellipsoids set at 50% probability level. All hydrogen atoms and co-crystallized C_6H_6 omitted for clarity. Selected bond distances (Å): Y(1)–N(1): 2.1952(13); Y(2)–N(2): 2.2060(13); Y(1)···B(1): 2.650(2); Y(1)···B(2): 2.732(2); Y(1)···B(3): 2.506(2); Y(2)···B(1): 2.824(2); Y(2)···B(4): 2.550(2); Y(2)···B(5): 2.495(2); K(1)···B(2): 3.138(2); K(1)···B(2): 3.617(2).

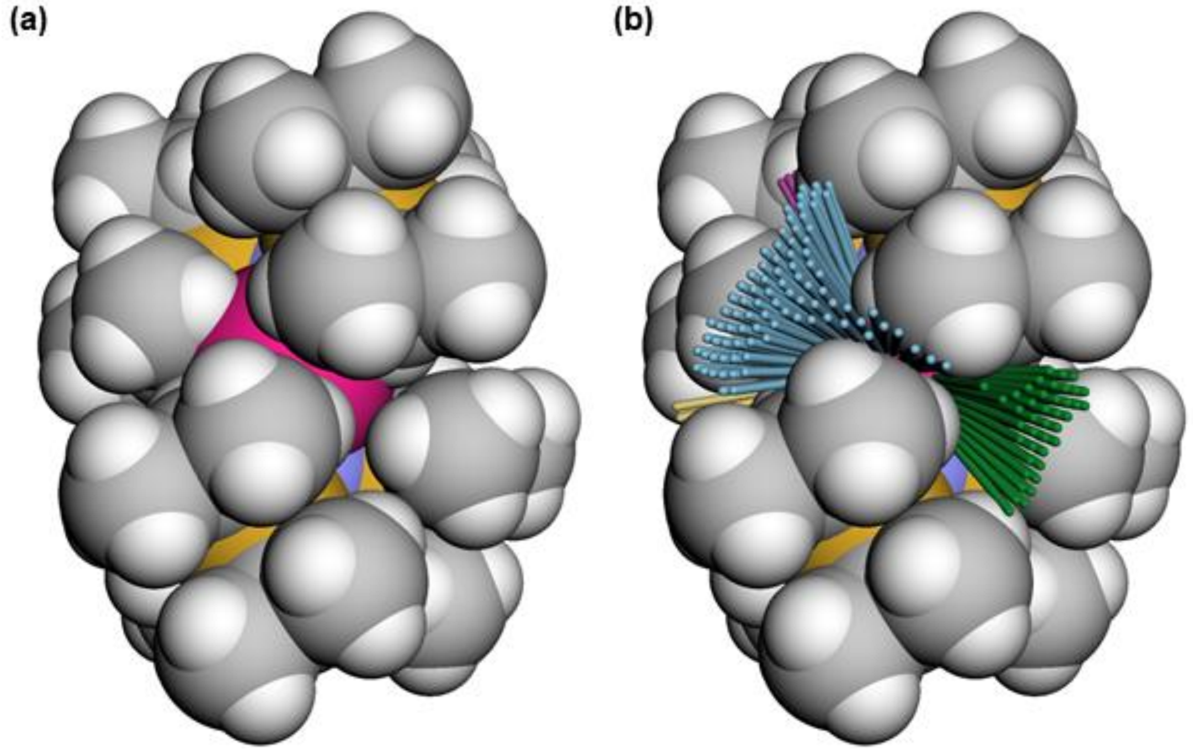

Figure S71. (a) Space-filling model of the cation of **1-Dy**. Color code: Dy (pink), silicon (gold), nitrogen (violet), carbon (grey) and hydrogen (white). (b) Using the *AtomAccess* program,[2] the accessibility of the Dy atom in the cation of **1-Dy** is determined by ray-tracing, and the unblocked rays are grouped into adjacent clusters (light blue, green, yellow, magenta). At the Dy atom, a total of 6.5% of the solid angle is exposed, with the largest cluster (light blue) comprising of 3.4% of the solid angle.

6. Powder X-ray diffraction

(a)

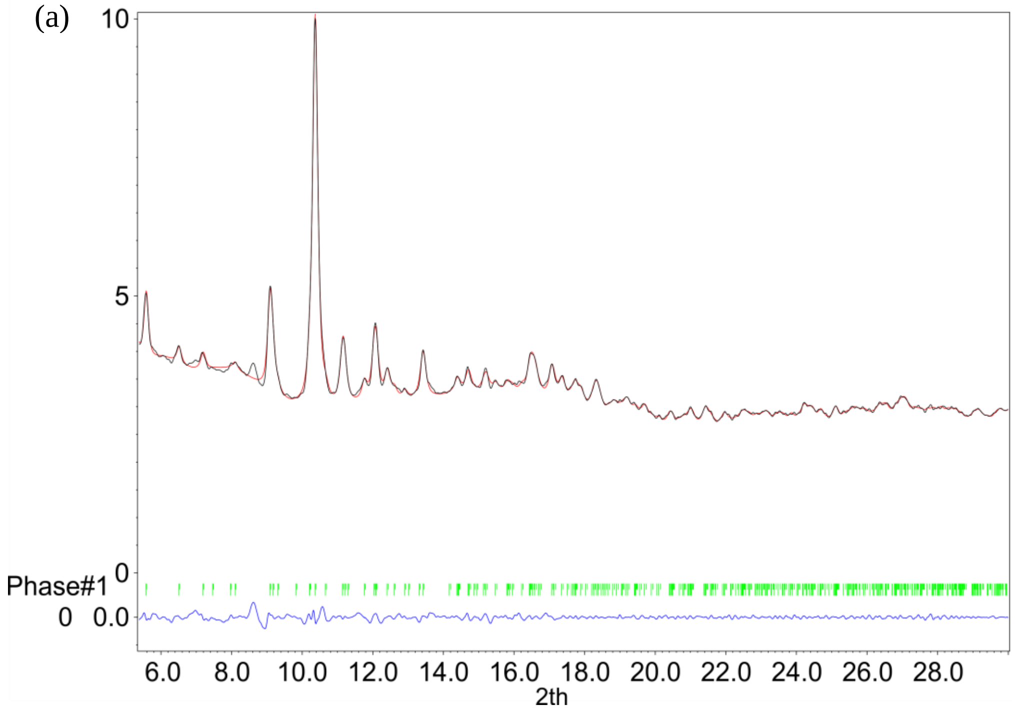

(b)

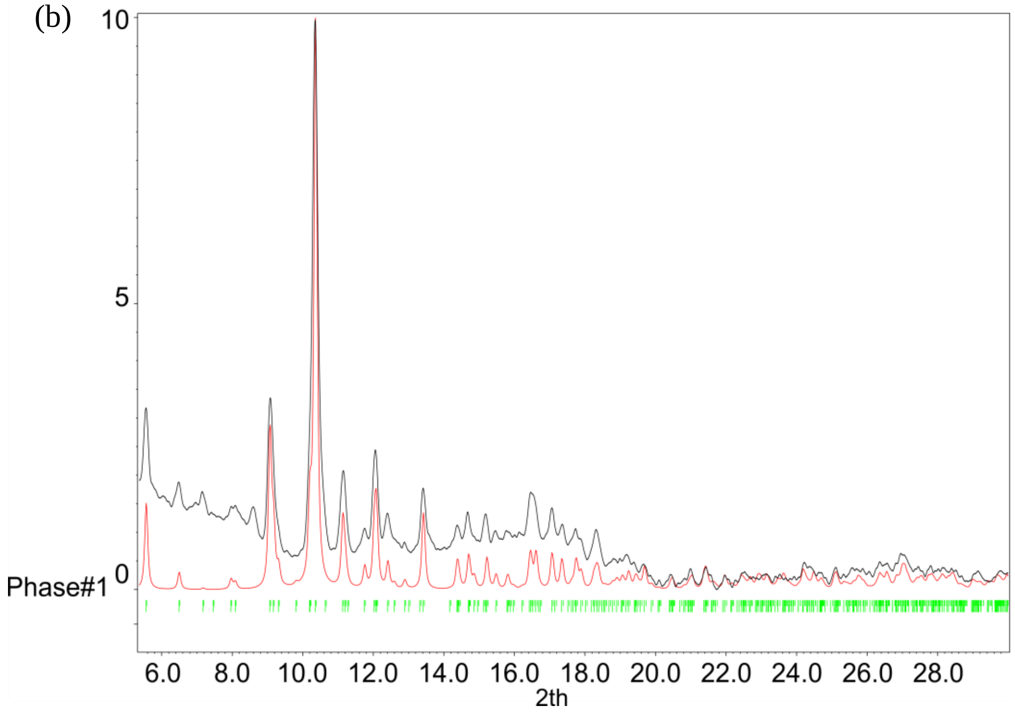

Figure S72. (a) Le Bail refinement analysis of **1-Dy** at 100 K. Experimental powder X-ray diffraction pattern (black), modelled data (red) and difference (blue). (b) Theoretical powder X-ray diffraction pattern simulated from single crystal X-ray diffraction at 100 K (red) compared to experimental pattern at 100 K (black).

Table S4. Unit cell values obtained from Le Bail refinement results for **1-Dy**.

**Rwp**	**Rwp′**	***a***	***b***	***c***	***α***	***β***	***γ***
2.222	11.385	19.53(3)	23.74(4)	31.80(5)	90	90	90

7. Magnetic measurements

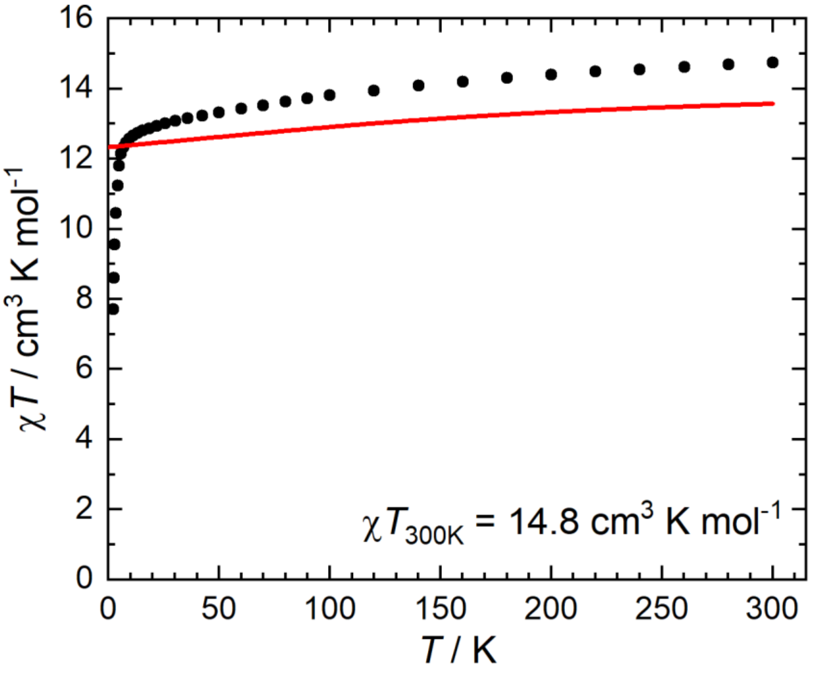

Figure S73. Temperature dependence of the molar magnetic susceptibility product (χT) for **1-Dy** suspended in eicosane (black circles) measured under a 0.1 T dc magnetic field and calculated CASSCF susceptibility (red line).

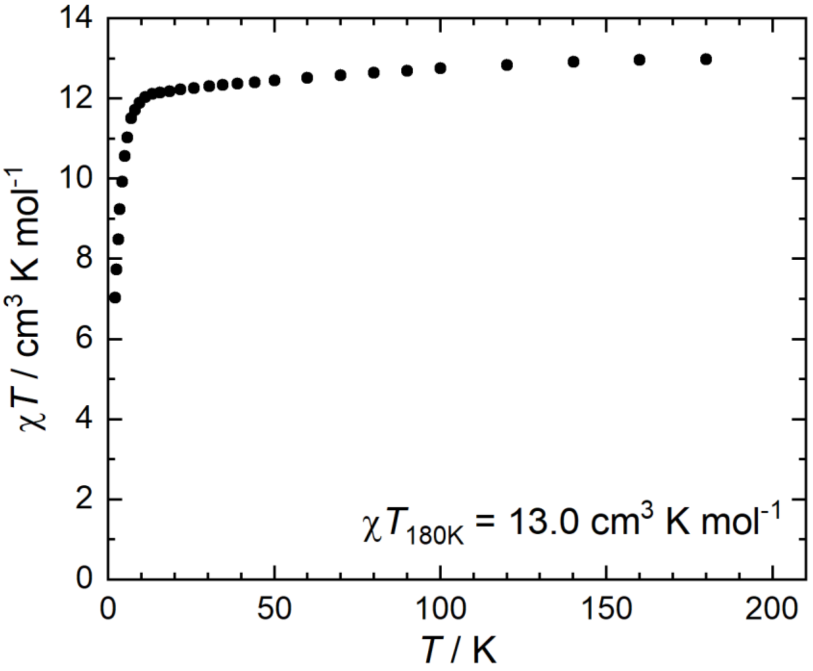

Figure S74. Temperature dependence of the molar magnetic susceptibility product (χT) for frozen solution of 200 mM **1-Dy** in fluorobenzene measured under a 0.1 T dc magnetic field.

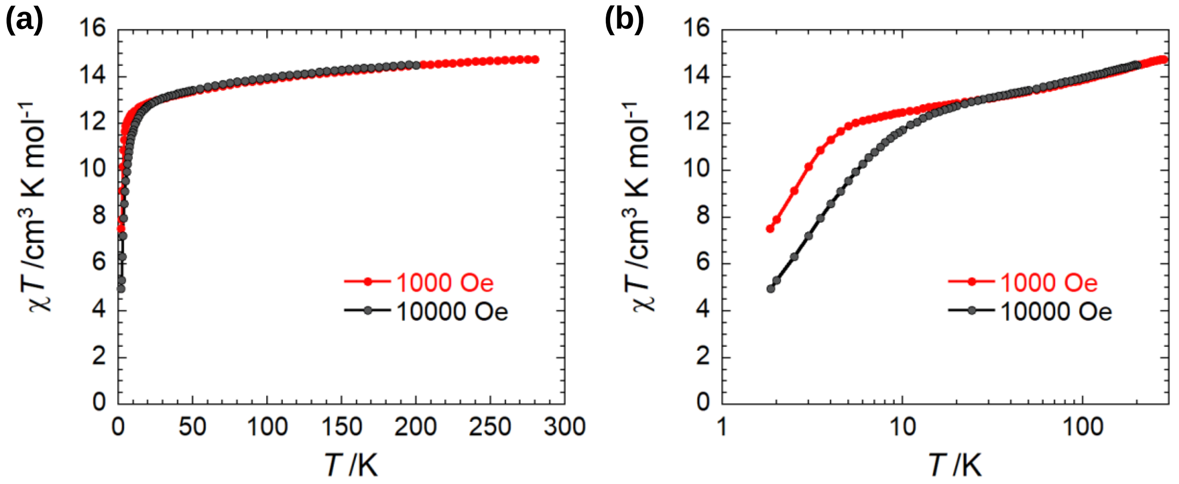

Figure S75. Temperature dependence of the molar magnetic susceptibility product (χT) for **1-Dy** suspended in mineral oil in a polypropylene bag measured under a 0.1 T (red) or 1 T (black) applied dc magnetic field, on (a) linear or (b) semi-logarithmic scale.

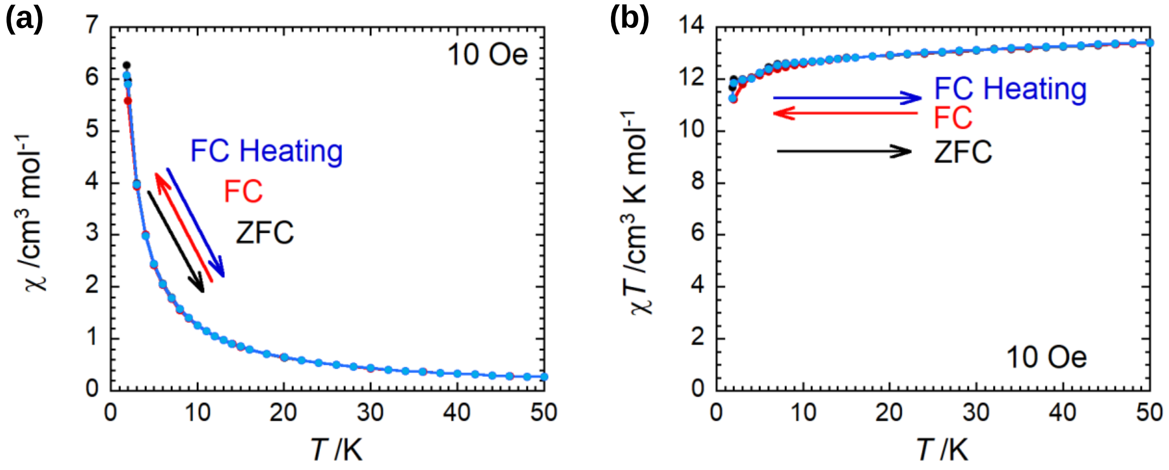

Figure S76. (a) Magnetic susceptibility (χ) and (b) molar magnetic susceptibility product (χT) *vs.* temperature (T) for **1-Dy** suspended in mineral oil in a polypropylene bag, under an applied dc field of 0.001 T, measured on warming after cooling in zero field (ZFC, black), on cooling in field (FC cool, red) and on warming in field (FC warm, blue).

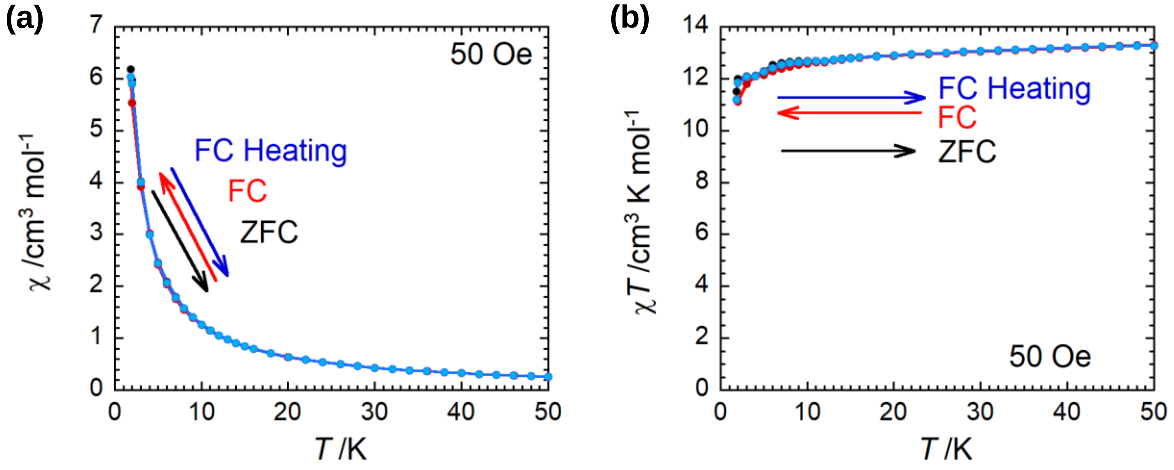

Figure S77. (a) Magnetic susceptibility (χ) and (b) molar magnetic susceptibility product (χ*T*) *vs.* temperature (*T*) for **1-Dy** suspended in mineral oil in a polypropylene bag, under an applied dc field of 0.005 T, measured on warming after cooling in zero field (ZFC, black), on cooling in field (FC cool, red) and on warming in field (FC warm, blue).

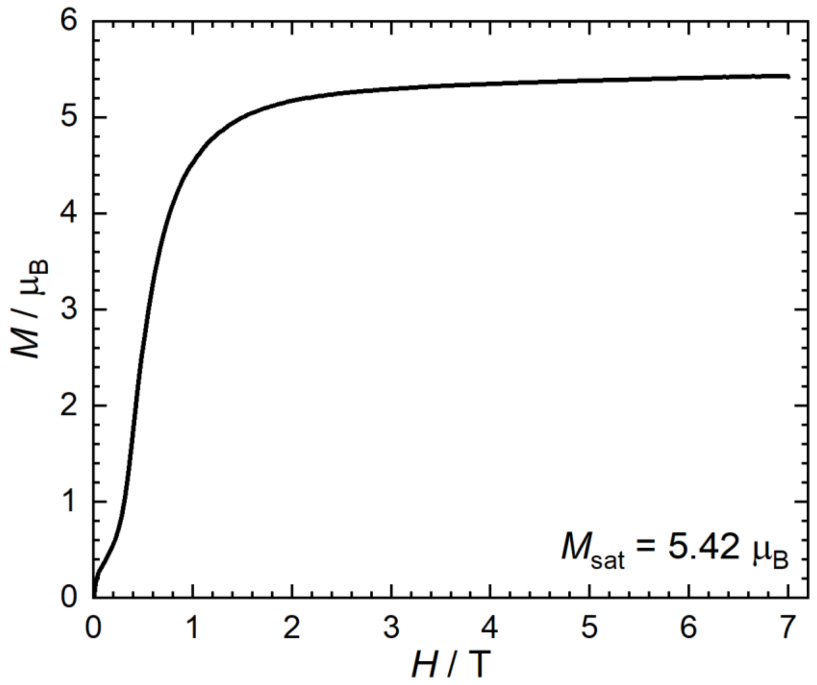

Figure S78. Field (*H*) dependence of the magnetization (*M*) (0–7 T) of **1-Dy** suspended in eicosane at 2 K after cooling in zero field. Sweep rate is 22 Oe s^{-1} (0.132 T min^{-1}).

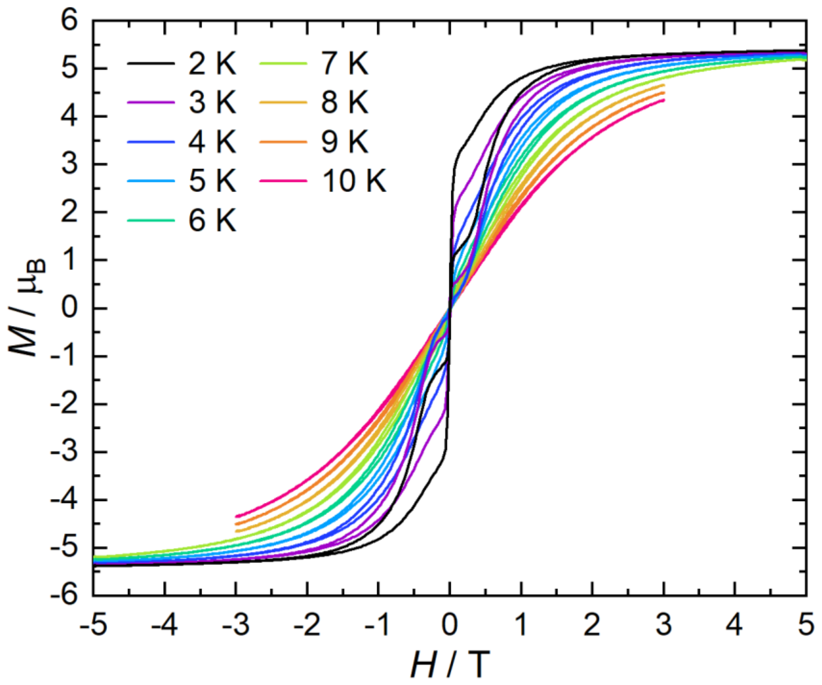

Figure S79. *M vs. H* hysteresis loops of **1-Dy** suspended in eicosane from 2 to 7 K in between –5 T to +5 T and from 8 to 10 K in between –3 T to +3 T. Sweep rate is 22 Oe s^{-1} (0.132 T min^{-1}).

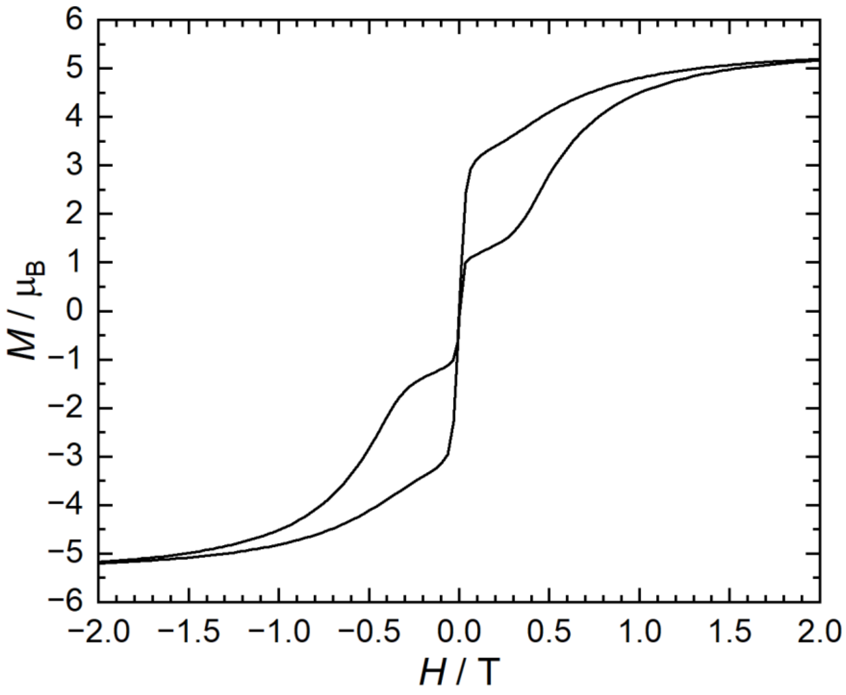

Figure S80. *M vs. H* hysteresis loop of **1-Dy** suspended in eicosane at 2 K, zoomed in between –2 T and 2 T. Sweep rate is 22 Oe s^{-1} (0.132 T min^{-1}).

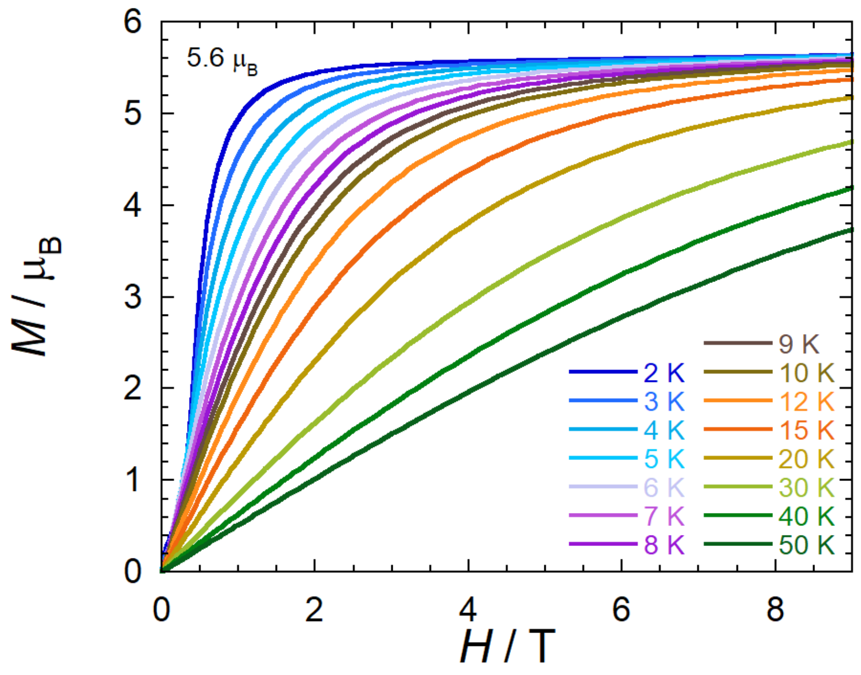

Figure S81. Field (*H*) dependence of the magnetization (*M*) (0–9 T) of **1-Dy** suspended in mineral oil in a polypropylene bag at 2–50 K after cooling in zero field. Sweep rate is 20 Oe s^{-1} (0.12 T min^{-1}).

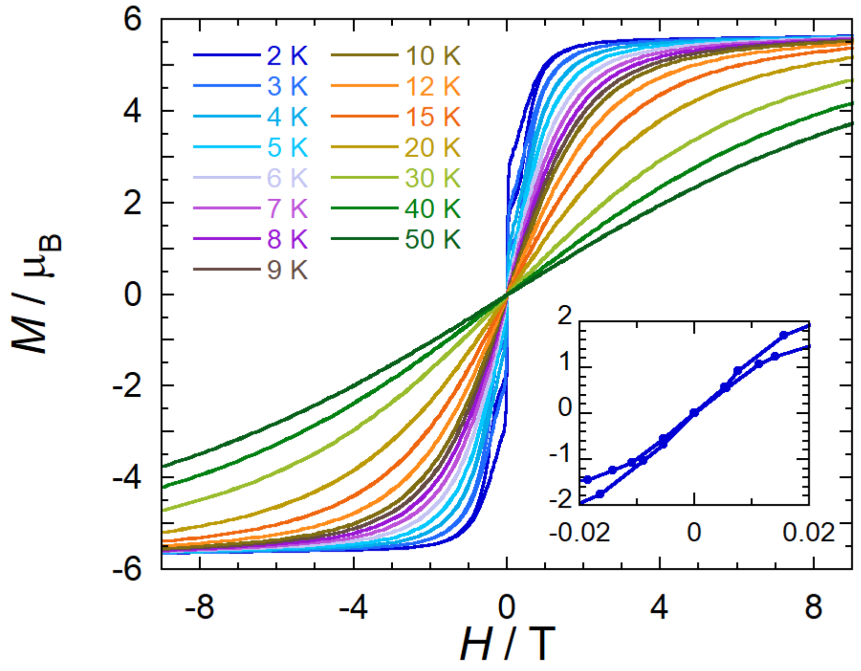

Figure S82. *M vs. H* hysteresis loops of **1-Dy** suspended in mineral oil in a polypropylene bag from 2 to 50 K in between –9 T to +9 T. Inset shows 2 K hysteresis closing at zero field. Sweep rate is 20 Oe s^{-1} (0.12 T min^{-1}).

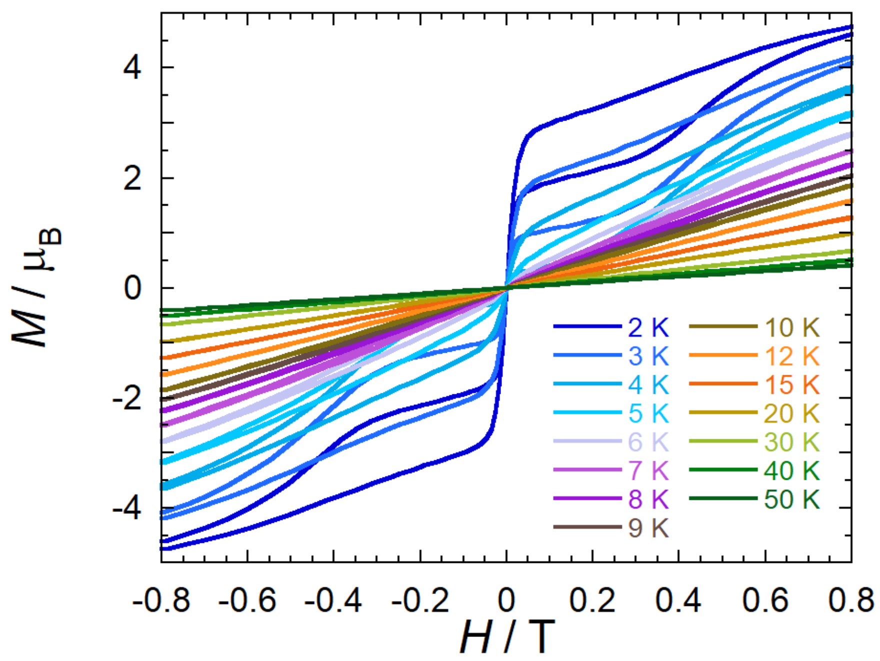

Figure S83. *M vs. H* hysteresis loops of **1-Dy** suspended in mineral oil in a polypropylene bag from 2 to 50 K, zoomed in to –0.8 T to +0.8 T. Sweep rate is 20 Oe s^{-1} (0.12 T min^{-1}).

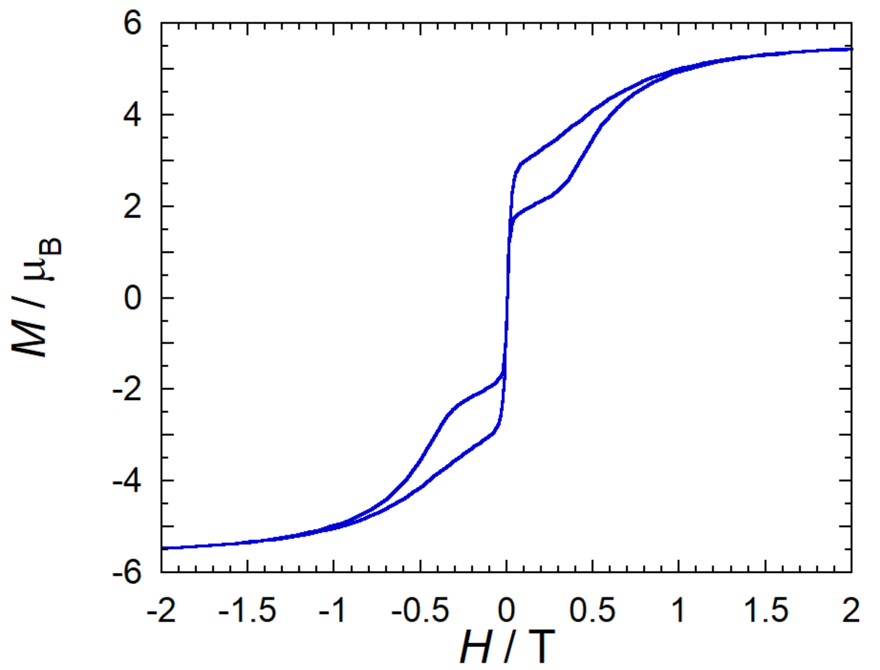

Figure S84. *M vs. H* hysteresis loops of **1-Dy** suspended in mineral oil in a polypropylene bag at 2 K, zoomed in to –2 T to +2 T. Sweep rate is 20 Oe s^{-1} (0.12 T min^{-1}).

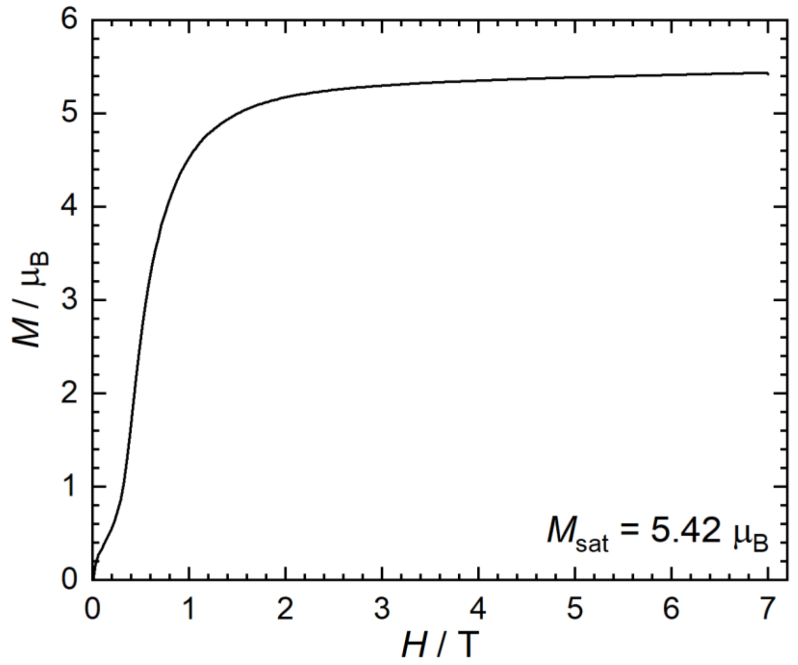

Figure S85. Field (*H*) dependence of the magnetization (0–7 T) of a 200 mM frozen solution of **1-Dy** in fluorobenzene at 2 K after cooling in zero field. Sweep rate is 22 Oe s^{-1} (0.132 T min^{-1}).

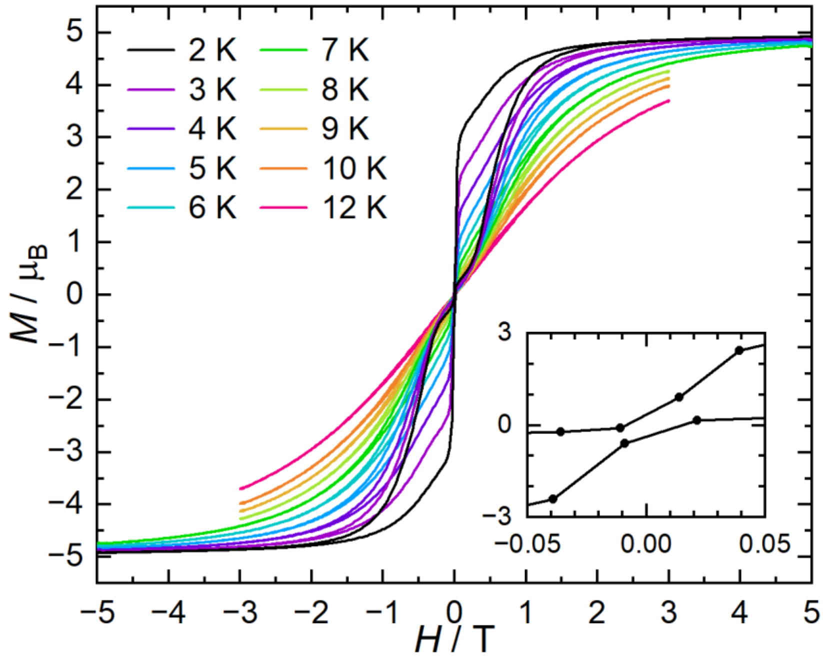

Figure S86. *M vs. H* hysteresis loops of a 200 mM frozen solution of **1-Dy** in fluorobenzene from 2 to 7 K in between –5 T to +5 T and from 8 to 12 K in between –3 T to +3 T. Inset shows 2 K hysteresis at low field. Sweep rate is 22 Oe s^{-1} (0.132 T min^{-1}).

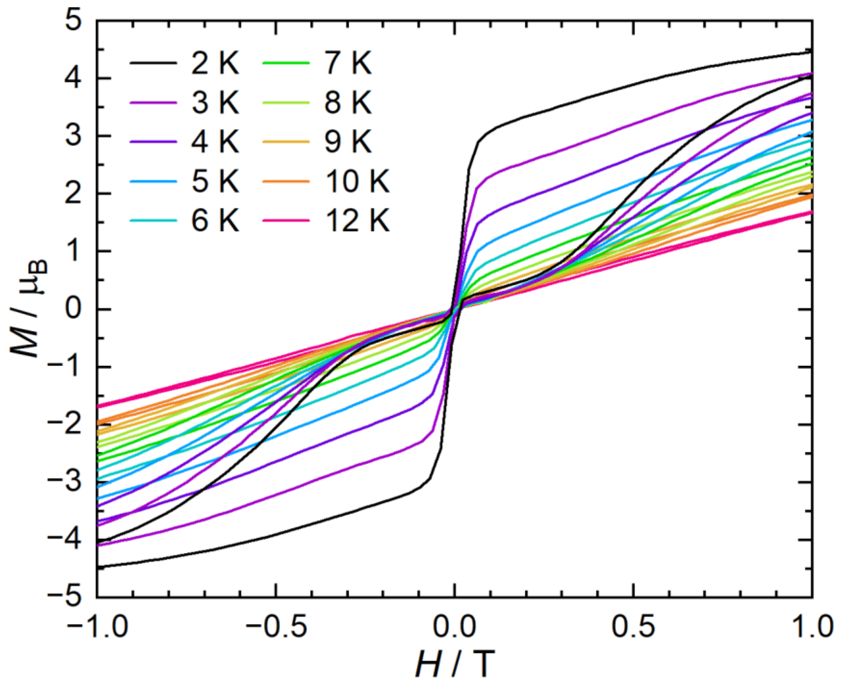

Figure S87. *M vs. H* hysteresis loops of a 200 mM frozen solution of **1-Dy** in fluorobenzene from 2–12 K, zoomed in to –1 T to +1 T. Sweep rate is 22 Oe s^{-1} (0.132 T min^{-1}).

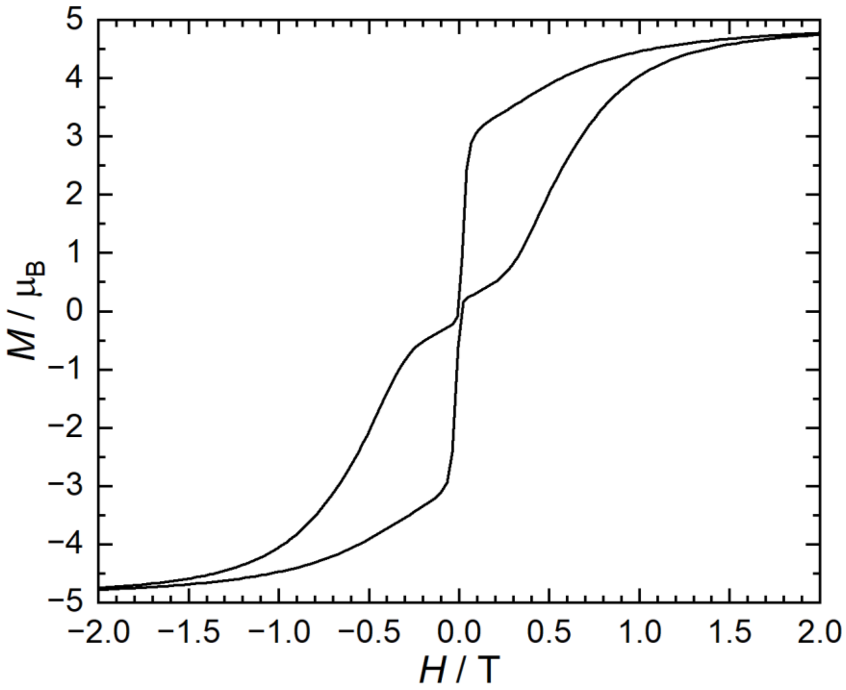

Figure S88. *M vs. H* hysteresis loop a 200 mM frozen solution of **1-Dy** in fluorobenzene at 2 K, zoomed in between –2 and +2 T. Sweep rate is 22 Oe s^{-1} (0.132 T min^{-1}).

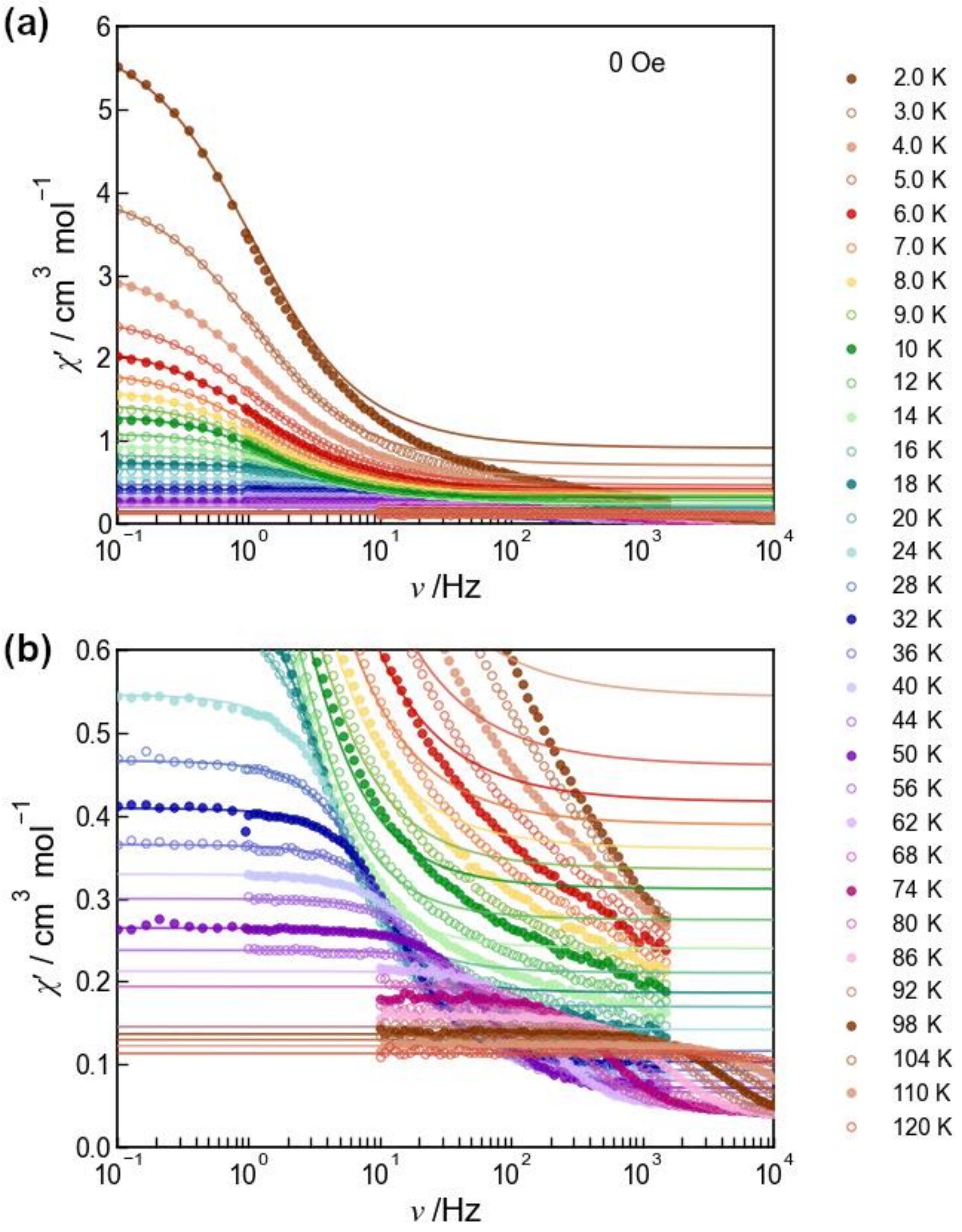

Figure S89. Fitting of the ac frequency dependence of the in-phase component of the ac susceptibility (χ′) for **1-Dy** suspended in mineral oil in a polypropylene bag in zero dc field to the generalized Debye model; (a) all data, (b) zoomed in to the high temperature region.

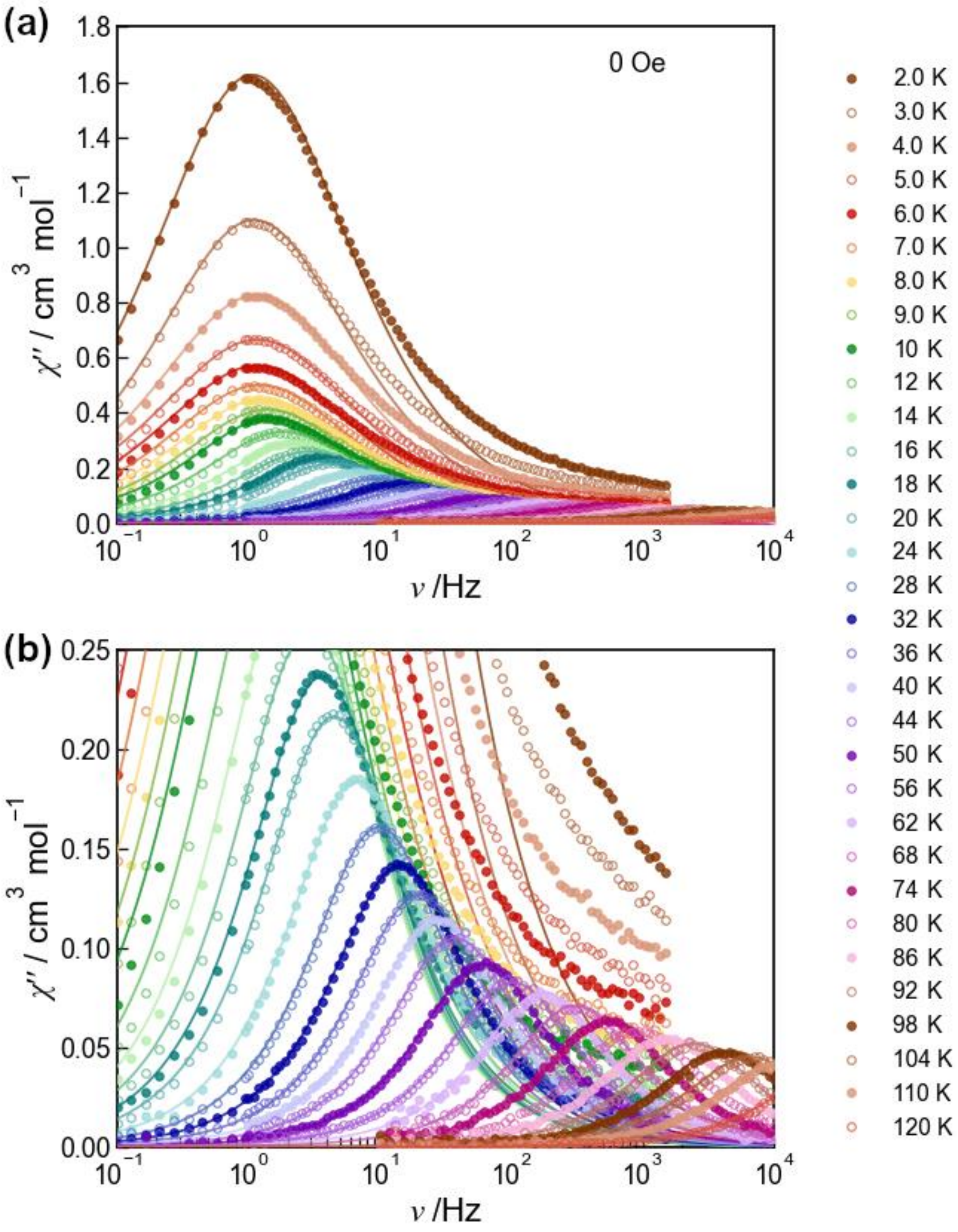

Figure S90. Fitting of the ac frequency dependence of the out-of-phase component of the ac susceptibility (χ″) for **1-Dy** suspended in mineral oil in a polypropylene bag in zero dc field to the generalized Debye model; (a) all data, (b) zoomed in to the high temperature region.

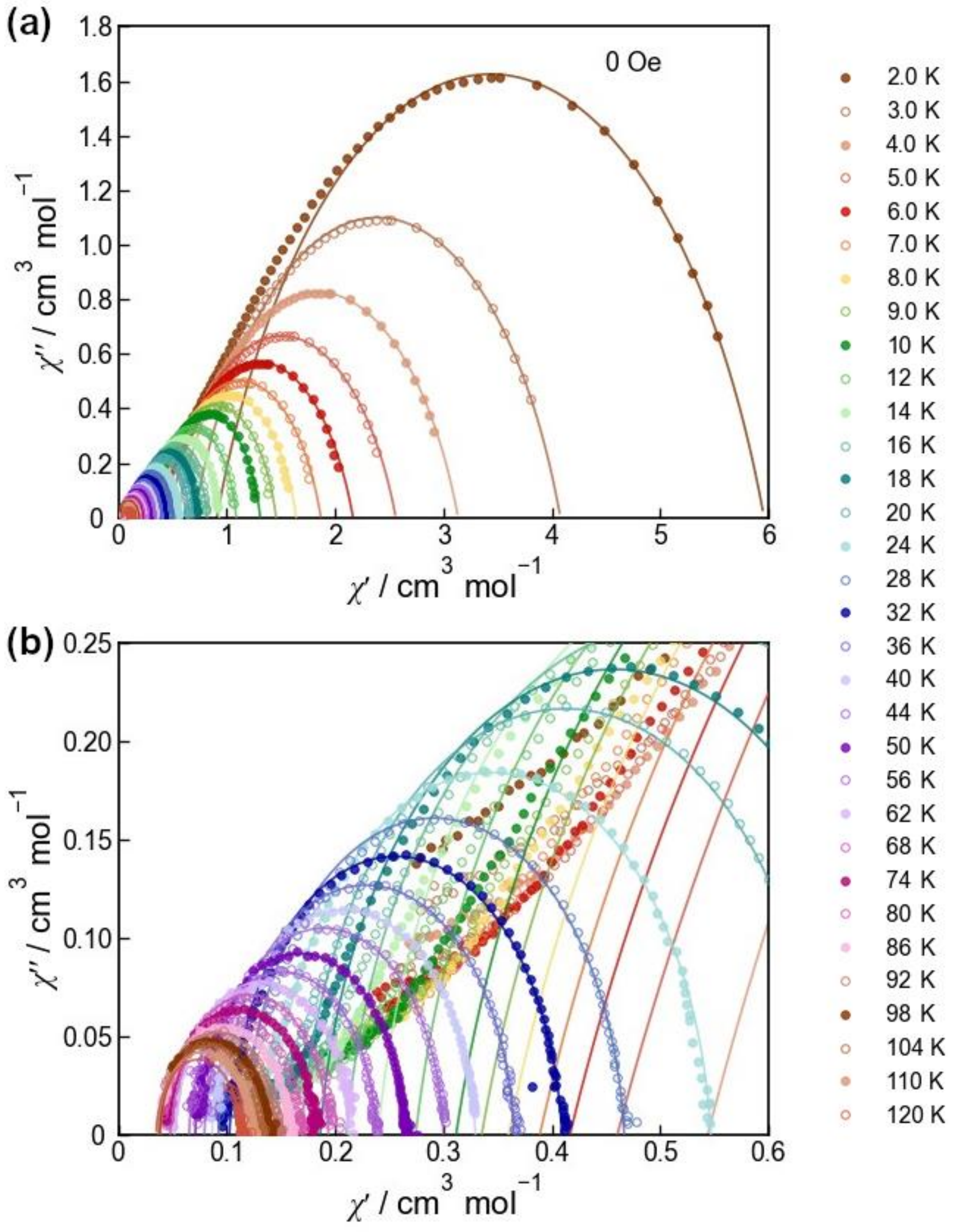

Figure S91. Cole-Cole plot showing fitting of ac data for **1-Dy** suspended in mineral oil in a polypropylene bag in zero dc field to the generalized Debye model; (a) all data, (b) zoomed-in to the high temperature region.

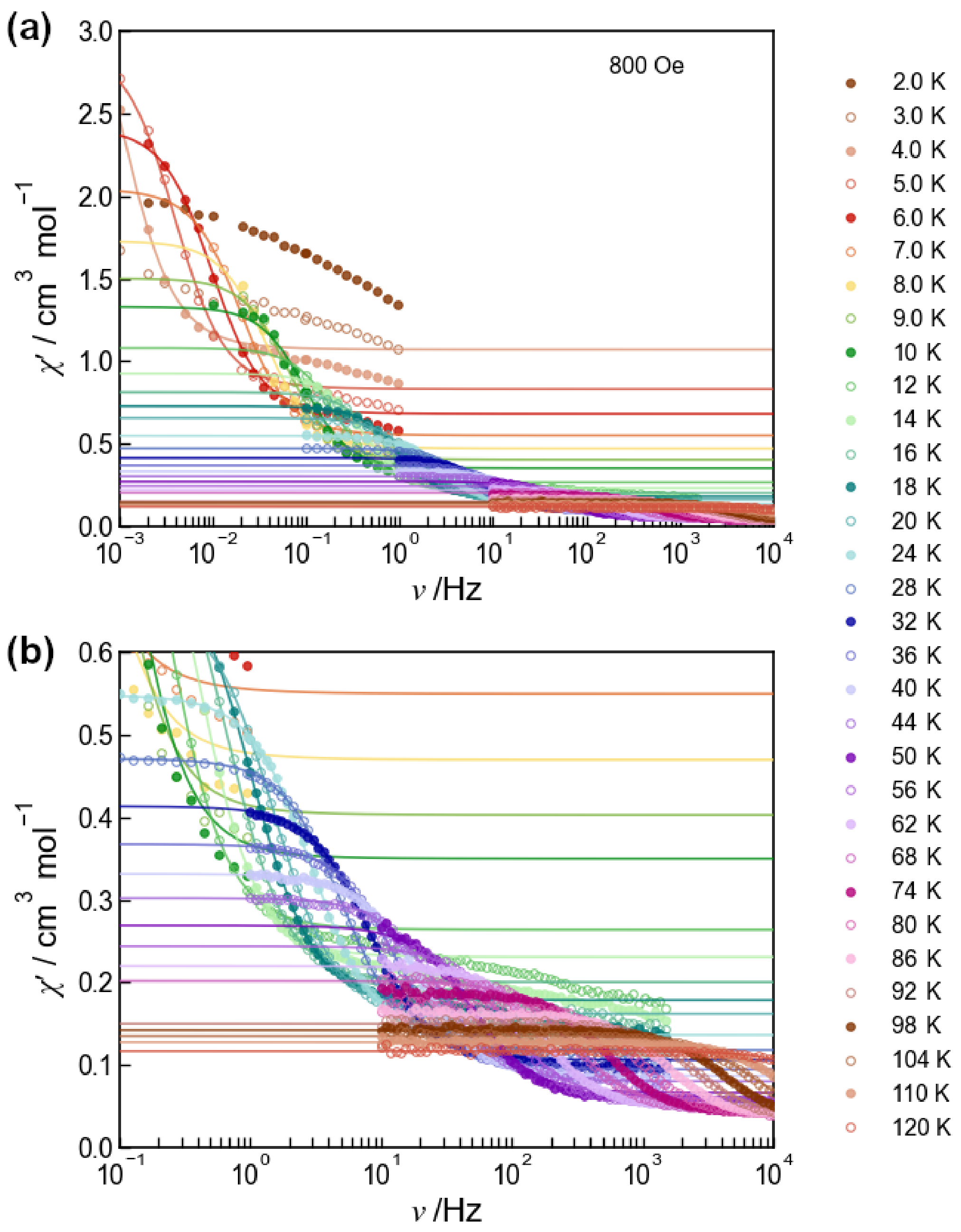

Figure S92. Fitting of the ac frequency dependence of the in-phase component of the ac susceptibility (χ') for **1-Dy** suspended in mineral oil in a polypropylene bag in a 0.08 T dc field to the generalized Debye model; (a) all data, (b) zoomed in to the high temperature region.

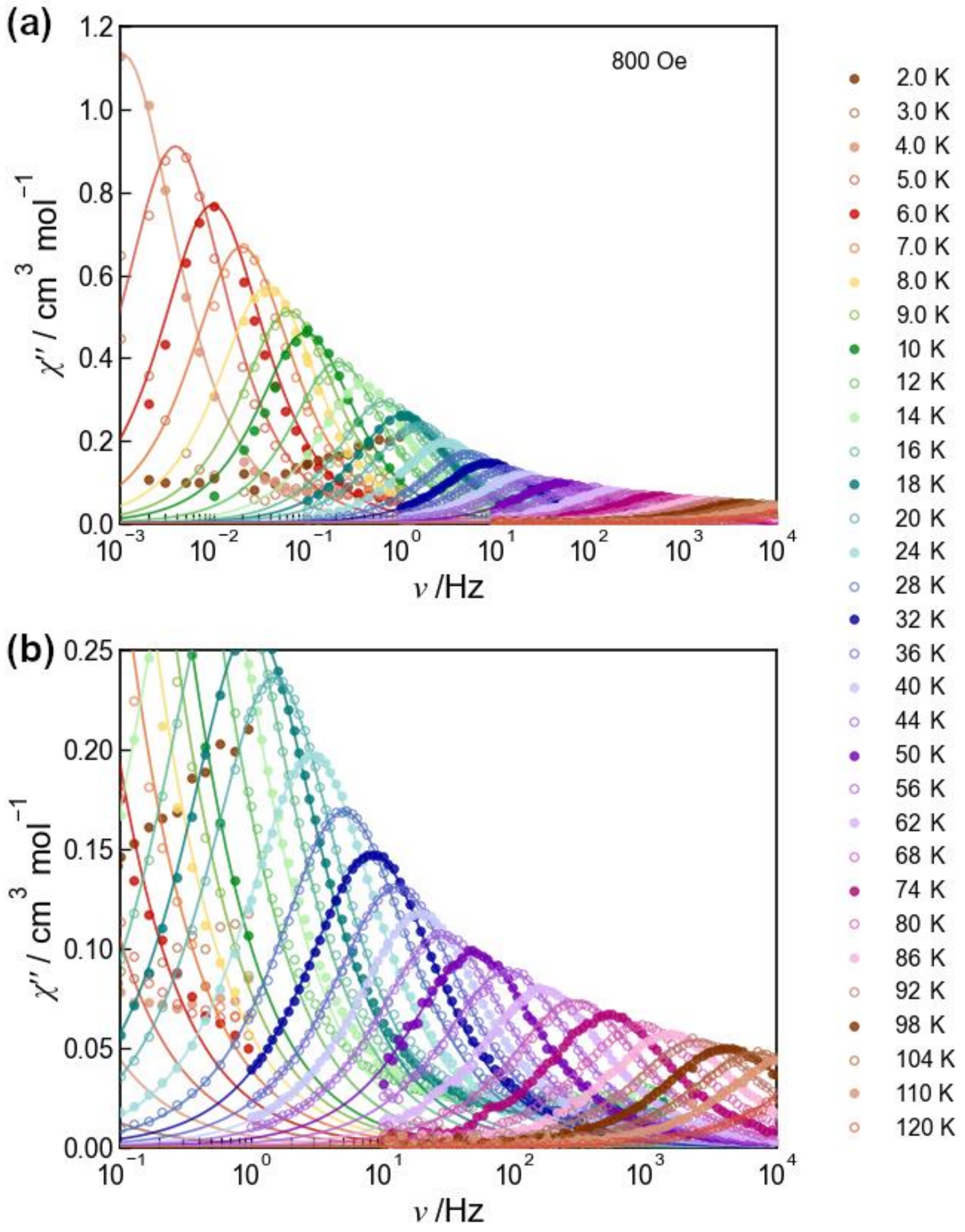

Figure S93. Fitting of the ac frequency dependence of the out-of-phase component of the ac susceptibility (χ′′) for **1-Dy** suspended in mineral oil in a polypropylene bag in a 0.08 T dc field to the generalized Debye model; (a) all data, (b) zoomed in to the high temperature region.

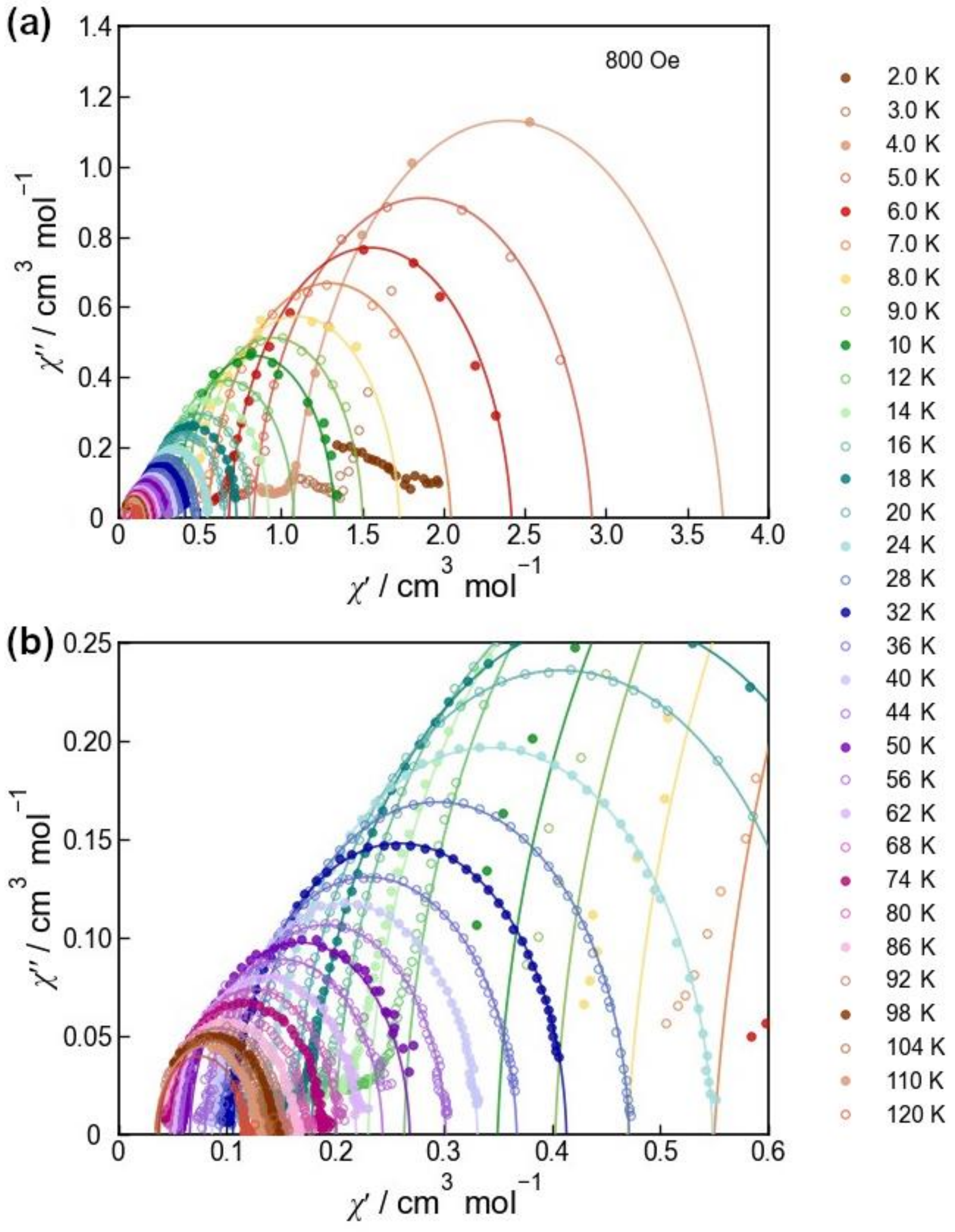

Figure S94. Cole-Cole plot showing fitting of ac data for **1-Dy** suspended in mineral oil in a polypropylene bag in a 0.08 T dc field to the generalized Debye model; (a) all data, (b) zoomed in to the high temperature region.

Table S5. Best fit parameters to the generalized Debye model for **1-Dy** suspended in mineral oil in a polypropylene bag in zero dc field. Note that the α parameter has been fixed at 0.014 for T ≥ 80 K according to its evolution observed at lower temperature.

T (K)	τ (s)	χ_S (cm^3 mol^{-1})	χ_T (cm^3 mol^{-1})	α
2	1.47251E-01	0.9100	5.9600	0.2717
3	1.43259E-01	0.7000	4.0800	0.2654
4	1.37433E-01	0.5440	3.1368	0.2772
5	1.35415E-01	0.4605	2.5618	0.2808
6	1.34738E-01	0.4168	2.1666	0.2633
7	1.30086E-01	0.3891	1.8696	0.2426
8	1.22685E-01	0.3601	1.6428	0.2204
9	1.13390E-01	0.3354	1.4570	0.1929
10	1.04292E-01	0.3118	1.3098	0.1673
12	8.55904E-02	0.2744	1.0885	0.1294
14	6.83684E-02	0.2397	0.9324	0.1060
16	5.43279E-02	0.2103	0.8194	0.1001
18	4.37083E-02	0.1864	0.7288	0.0861
20	3.53695E-02	0.1692	0.6561	0.0739
22	2.87692E-02	0.1541	0.5969	0.0672
24	2.34606E-02	0.1419	0.5467	0.0598
26	1.93606E-02	0.1234	0.5042	0.0617
28	1.60179E-02	0.1158	0.4668	0.0546
30	1.33408E-02	0.1089	0.4362	0.0514
32	1.10888E-02	0.1025	0.4094	0.0511
34	9.38239E-03	0.0969	0.3871	0.0523
36	7.88232E-03	0.0924	0.3653	0.0459
38	6.73419E-03	0.0889	0.3469	0.0443
40	5.75149E-03	0.0865	0.3295	0.0388
42	4.91046E-03	0.0835	0.3141	0.0349
44	4.21559E-03	0.0795	0.3000	0.0305
46	3.55806E-03	0.0775	0.2877	0.0348
48	3.04710E-03	0.0738	0.2751	0.0302
50	2.56982E-03	0.0711	0.2645	0.0310
52	2.18683E-03	0.0698	0.2554	0.0308
54	1.84390E-03	0.0683	0.2456	0.0220
56	1.54242E-03	0.0654	0.2376	0.0254
58	1.30371E-03	0.0645	0.2300	0.0244
60	1.08168E-03	0.0520	0.2187	0.0245
62	9.04788E-04	0.0520	0.2123	0.0192

64	7.52181E-04	0.0510	0.2062	0.0206
66	6.23123E-04	0.0490	0.1994	0.0197
68	5.17515E-04	0.0480	0.1939	0.0163
70	4.34010E-04	0.0465	0.1885	0.0139
72	3.57971E-04	0.0455	0.1824	0.0134
74	2.99011E-04	0.0448	0.1780	0.0134
76	2.55042E-04	0.0450	0.1730	0.0146
78	2.12582E-04	0.0440	0.1690	0.0145
80	1.78283E-04	0.0430	0.1652	0.0140
82	1.50813E-04	0.0420	0.1616	0.0140
84	1.27502E-04	0.0410	0.1586	0.0140
86	1.08386E-04	0.0410	0.1555	0.0140
88	9.07831E-05	0.0400	0.1510	0.0140
90	7.72304E-05	0.0390	0.1480	0.0140
92	6.55410E-05	0.0390	0.1453	0.0140
94	5.53469E-05	0.0380	0.1420	0.0140
96	4.62624E-05	0.0380	0.1393	0.0140
98	3.94997E-05	0.0380	0.1364	0.0140
100	3.31994E-05	0.0370	0.1335	0.0140
102	2.79481E-05	0.0370	0.1315	0.0140
104	2.39388E-05	0.0370	0.1295	0.0140
106	2.01048E-05	0.0370	0.1269	0.0140
108	1.68074E-05	0.0360	0.1251	0.0140
110	1.45339E-05	0.0360	0.1222	0.0140
112	1.19157E-05	0.0360	0.1210	0.0140
114	9.84826E-06	0.0350	0.1190	0.0140
116	8.34653E-06	0.0350	0.1170	0.0140
118	7.12598E-06	0.0350	0.1150	0.0140
120	5.81534E-06	0.0350	0.1130	0.0140

Table S6. Best fit parameters to the generalized Debye model for **1-Dy** suspended in mineral oil in a polypropylene bag in a 0.08 T dc field. Note that the α parameter has been fixed at 0.025 for T ≥ 50 K according to its evolution observed at lower temperature.

T (K)	τ (s)	χ_S (cm^3 mol^{-1})	χ_T (cm^3 mol^{-1})	α
4	1.45957E+02	1.0700	3.7200	0.1000
5	4.04141E+01	0.8300	2.9144	0.0854
6	1.62528E+01	0.6800	2.4200	0.0780
7	7.96232E+00	0.5500	2.0469	0.0722
8	4.19433E+00	0.4700	1.7300	0.0600
9	2.49586E+00	0.4032	1.5032	0.0451
10	1.64717E+00	0.3500	1.3300	0.0390
12	7.51276E-01	0.2639	1.0801	0.0287
14	4.06563E-01	0.2307	0.9253	0.0215
16	2.42777E-01	0.2003	0.8119	0.0234
18	1.56648E-01	0.1785	0.7276	0.0291
20	1.05718E-01	0.1619	0.6558	0.0284
22	7.48106E-02	0.1485	0.5978	0.0267
24	5.45765E-02	0.1361	0.5486	0.0296
26	4.08545E-02	0.1262	0.5072	0.0297
28	3.12743E-02	0.1181	0.4715	0.0279
30	2.42430E-02	0.1112	0.4402	0.0252
32	1.92033E-02	0.1057	0.4136	0.0247
34	1.53055E-02	0.0998	0.3893	0.0257
36	1.23688E-02	0.0942	0.3677	0.0280
38	1.01519E-02	0.0915	0.3492	0.0255
40	8.29934E-03	0.0864	0.3315	0.0273
42	6.85982E-03	0.0838	0.3175	0.0275
44	5.64387E-03	0.0797	0.3022	0.0277
46	4.66941E-03	0.0764	0.2896	0.0271
48	3.83669E-03	0.0734	0.2771	0.0246
50	3.17905E-03	0.0660	0.2690	0.0250
52	2.59928E-03	0.0640	0.2620	0.0250
54	2.13226E-03	0.0610	0.2510	0.0250
56	1.76461E-03	0.0590	0.2440	0.0250
58	1.43620E-03	0.0570	0.2370	0.0250
60	1.20377E-03	0.0530	0.2272	0.0250
62	9.73515E-04	0.0520	0.2197	0.0250
64	8.01638E-04	0.0510	0.2132	0.0250
66	6.55860E-04	0.0480	0.2058	0.0250

68	5.43950E-04	0.0480	0.2017	0.0250
70	4.48051E-04	0.0460	0.1953	0.0250
72	3.69712E-04	0.0455	0.1901	0.0250
74	3.08685E-04	0.0455	0.1855	0.0250
76	2.56518E-04	0.0450	0.1810	0.0250
78	2.13719E-04	0.0430	0.1762	0.0250
80	1.78518E-04	0.0420	0.1710	0.0250
82	1.50733E-04	0.0420	0.1680	0.0250
84	1.26104E-04	0.0410	0.1640	0.0250
86	1.06245E-04	0.0400	0.1600	0.0250
88	8.89631E-05	0.0400	0.1570	0.0250
90	7.69043E-05	0.0390	0.1530	0.0250
92	6.48354E-05	0.0380	0.1500	0.0250
94	5.48439E-05	0.0380	0.1470	0.0250
96	4.64501E-05	0.0380	0.1440	0.0250
98	3.90646E-05	0.0370	0.1420	0.0250
100	3.29244E-05	0.0370	0.1392	0.0250
102	2.71707E-05	0.0360	0.1365	0.0250
104	2.33151E-05	0.0360	0.1350	0.0250
106	1.93660E-05	0.0355	0.1320	0.0250
108	1.62626E-05	0.0345	0.1290	0.0250
110	1.36940E-05	0.0343	0.1274	0.0250
112	1.14634E-05	0.0342	0.1248	0.0250
114	9.57551E-06	0.0343	0.1223	0.0250
116	8.05873E-06	0.0343	0.1205	0.0250
118	6.90180E-06	0.0343	0.1185	0.0250
120	5.62728E-06	0.0343	0.1167	0.0250

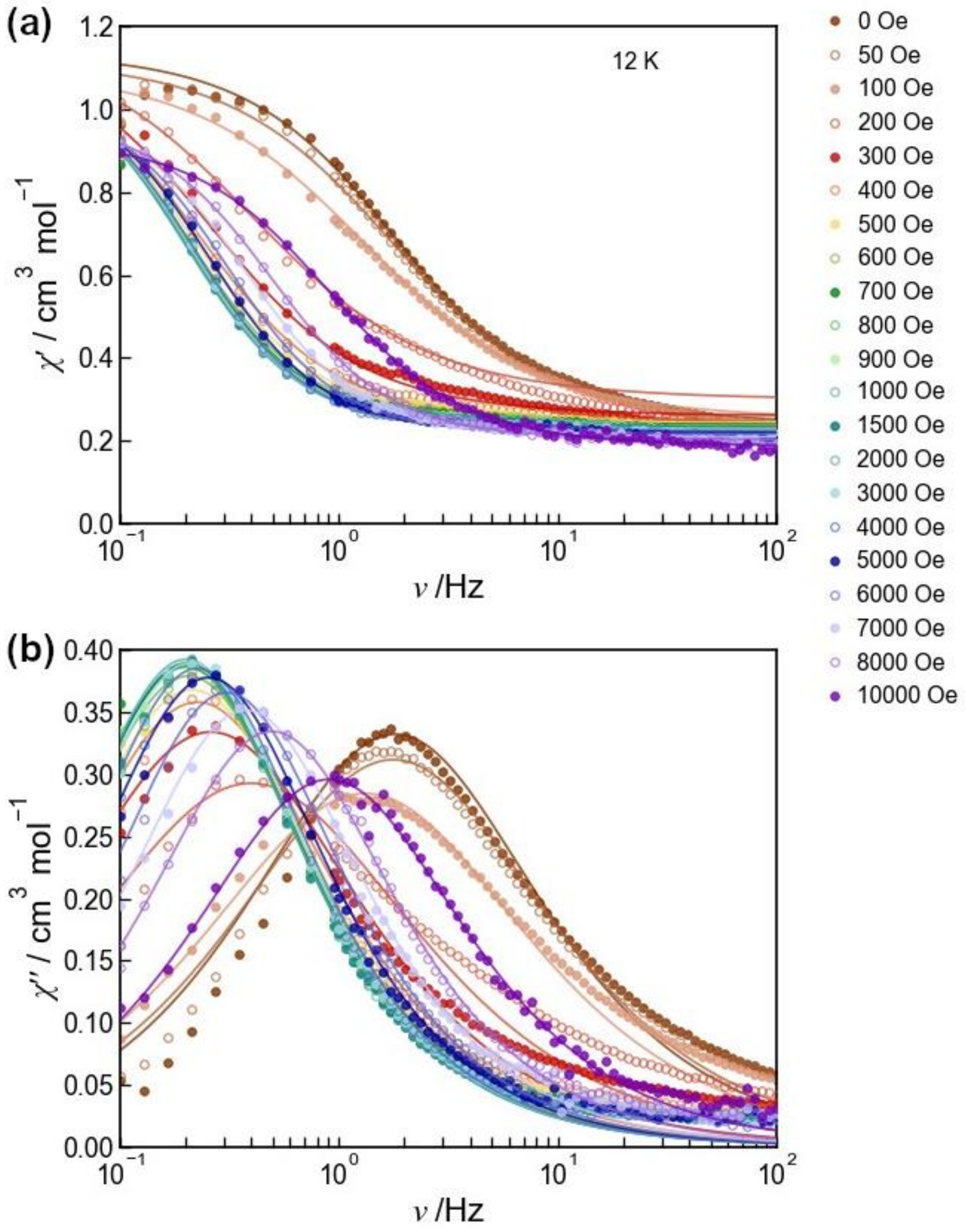

Figure S95. Fitting the ac frequency dependence of the (a) in-phase (χ′) and (b) out-of-phase (χ″) components of the ac susceptibility for **1-Dy** suspended in mineral oil in a polypropylene bag at 12 K and variable dc field to the generalized Debye model.

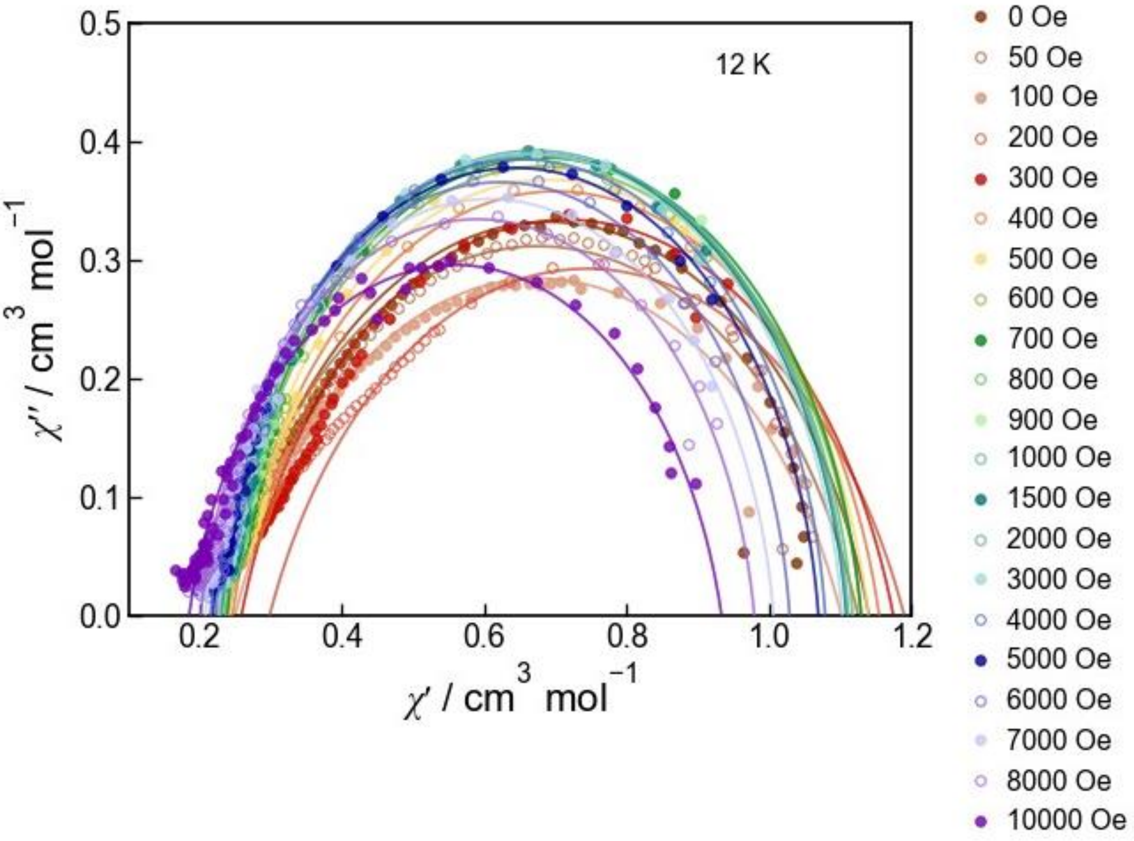

Figure S96. Cole-Cole plot showing fitting of ac data for **1-Dy** suspended in mineral oil in a polypropylene bag at 12 K and variable dc field to the generalized Debye model.

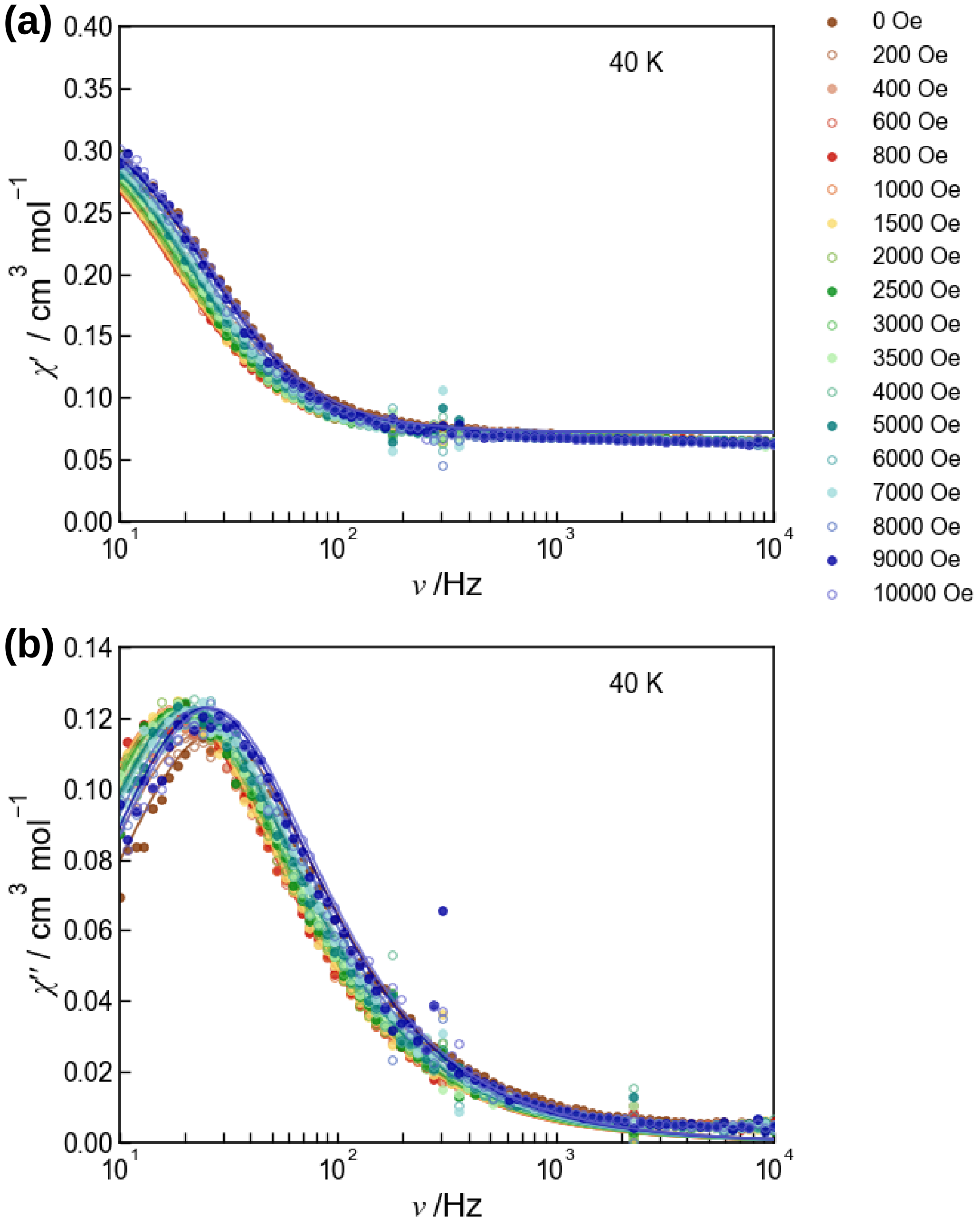

Figure S97. Fitting the ac frequency dependence of the (a) in-phase (χ′) and (b) out-of-phase (χ′′) components of the ac susceptibility for **1-Dy** suspended in mineral oil in a polypropylene bag at 40 K and variable dc field to the generalized Debye model.

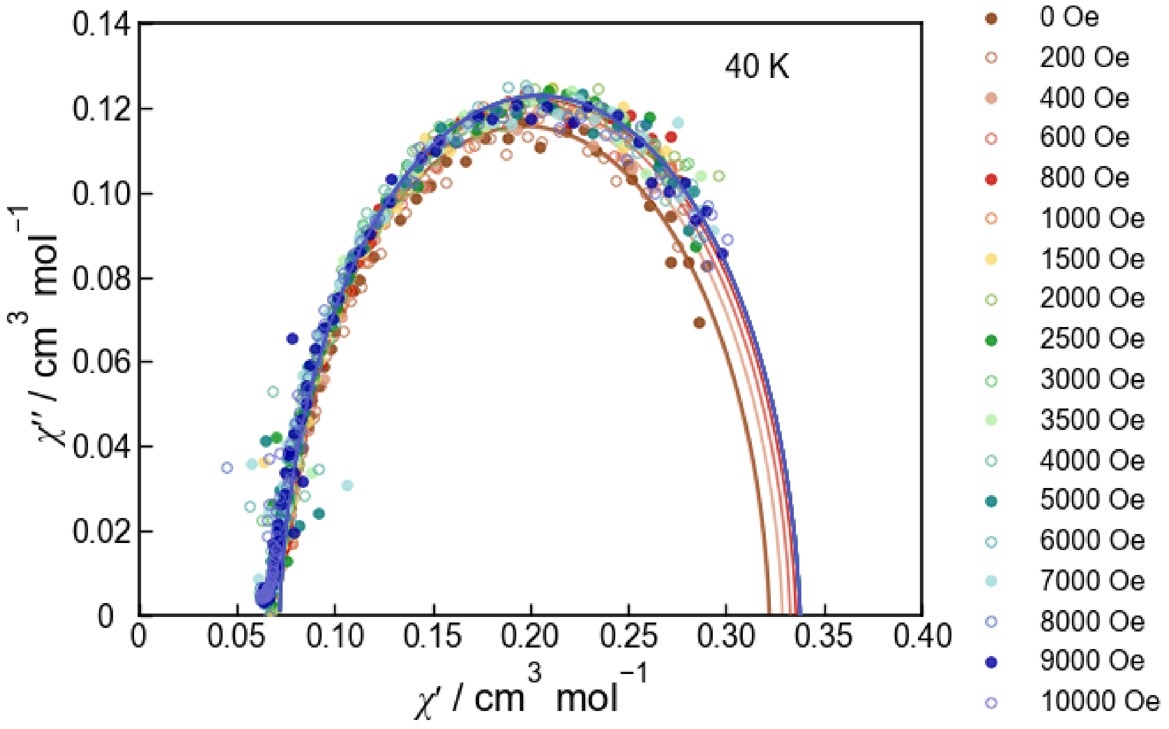

Figure S98. Cole-Cole plot showing fitting of ac data for **1-Dy** suspended in mineral oil in a polypropylene bag at 40 K and variable dc field to the generalized Debye model.

Table S7. Best fit parameters to the generalized Debye model for **1-Dy** suspended in mineral oil in a polypropylene bag in at 12 K and variable dc field.

H (Oe)	τ (s)	χ_S (cm^3 mol^{-1})	χ_T (cm^3 mol^{-1})	α
0	8.76416E-02	0.24000	1.14100	0.19054
50	8.95203E-02	0.22571	1.12571	0.22815
100	1.20253E-01	0.25000	1.10218	0.25000
200	3.99786E-01	0.29856	1.19057	0.26000
300	6.09480E-01	0.25899	1.17527	0.19746
400	7.01920E-01	0.24639	1.15543	0.15000
500	7.42237E-01	0.24205	1.14030	0.12577
600	7.72583E-01	0.23816	1.13025	0.10270
700	7.90682E-01	0.23855	1.13002	0.09000
800	7.95703E-01	0.23602	1.11806	0.08159
900	7.87238E-01	0.23509	1.12188	0.07900
1500	7.81688E-01	0.23127	1.10906	0.07506
2000	7.91849E-01	0.22994	1.11207	0.07373
3000	7.52655E-01	0.22690	1.10312	0.07378
4000	7.01277E-01	0.22324	1.07953	0.06670
5000	6.23681E-01	0.21885	1.06997	0.07496
6000	5.15704E-01	0.21561	1.02939	0.06724
7000	4.21697E-01	0.20850	1.00678	0.08017
8000	3.20551E-01	0.20185	0.97986	0.09527
10000	1.75568E-01	0.18550	0.93379	0.14775

Table S8. Best fit parameters to the generalized Debye model for **1-Dy** suspended in mineral oil in a polypropylene bag in at 40 K and variable dc field. Note that the α parameter has been fixed at 0.05, χ_S has been fixed at 0.072 and for non-zero fields χ_T has been fixed at the value indicated.

H (Oe)	τ (s)	χ_S (cm^3 mol^{-1})	χ_T (cm^3 mol^{-1})	α
0	5.78755E-03	7.2E-02	3.2264E-01	0.05
200	6.70644E-03	7.2E-02	3.22E-01	0.05
400	7.94366E-03	7.2E-02	3.29E-01	0.05
600	8.42191E-03	7.2E-02	3.33E-01	0.05
800	8.83600E-03	7.2E-02	3.36E-01	0.05
1000	8.82572E-03	7.2E-02	3.38E-01	0.05
1500	8.68817E-03	7.2E-02	3.38E-01	0.05
2000	8.41760E-03	7.2E-02	3.38E-01	0.05
2500	8.13101E-03	7.2E-02	3.38E-01	0.05
3000	8.05019E-03	7.2E-02	3.38E-01	0.05
3500	7.82158E-03	7.2E-02	3.38E-01	0.05
4000	7.55457E-03	7.2E-02	3.38E-01	0.05
5000	7.45719E-03	7.2E-02	3.38E-01	0.05
6000	7.23738E-03	7.2E-02	3.38E-01	0.05
7000	7.08064E-03	7.2E-02	3.38E-01	0.05
8000	6.71477E-03	7.2E-02	3.38E-01	0.05
9000	6.30572E-03	7.2E-02	3.38E-01	0.05
10000	6.04170E-03	7.2E-02	3.38E-01	0.05

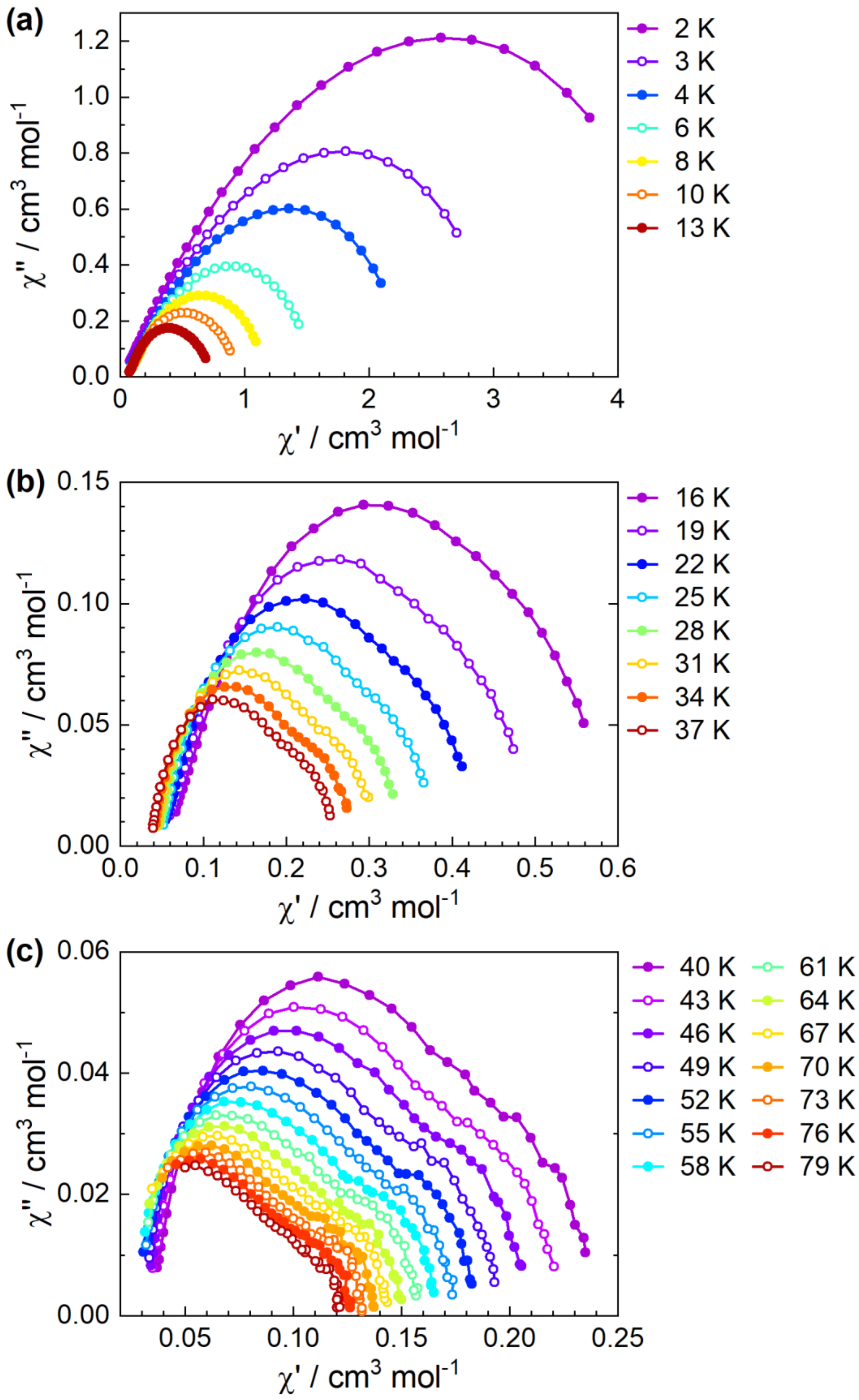

Figure S99. Cole-Cole plot showing ac data (a) 2–13 K, (b) 16–37 K, (c) 40–79 K for 200 mM frozen solution of **1-Dy** in fluorobenzene in zero dc field; lines are guides for the eyes.

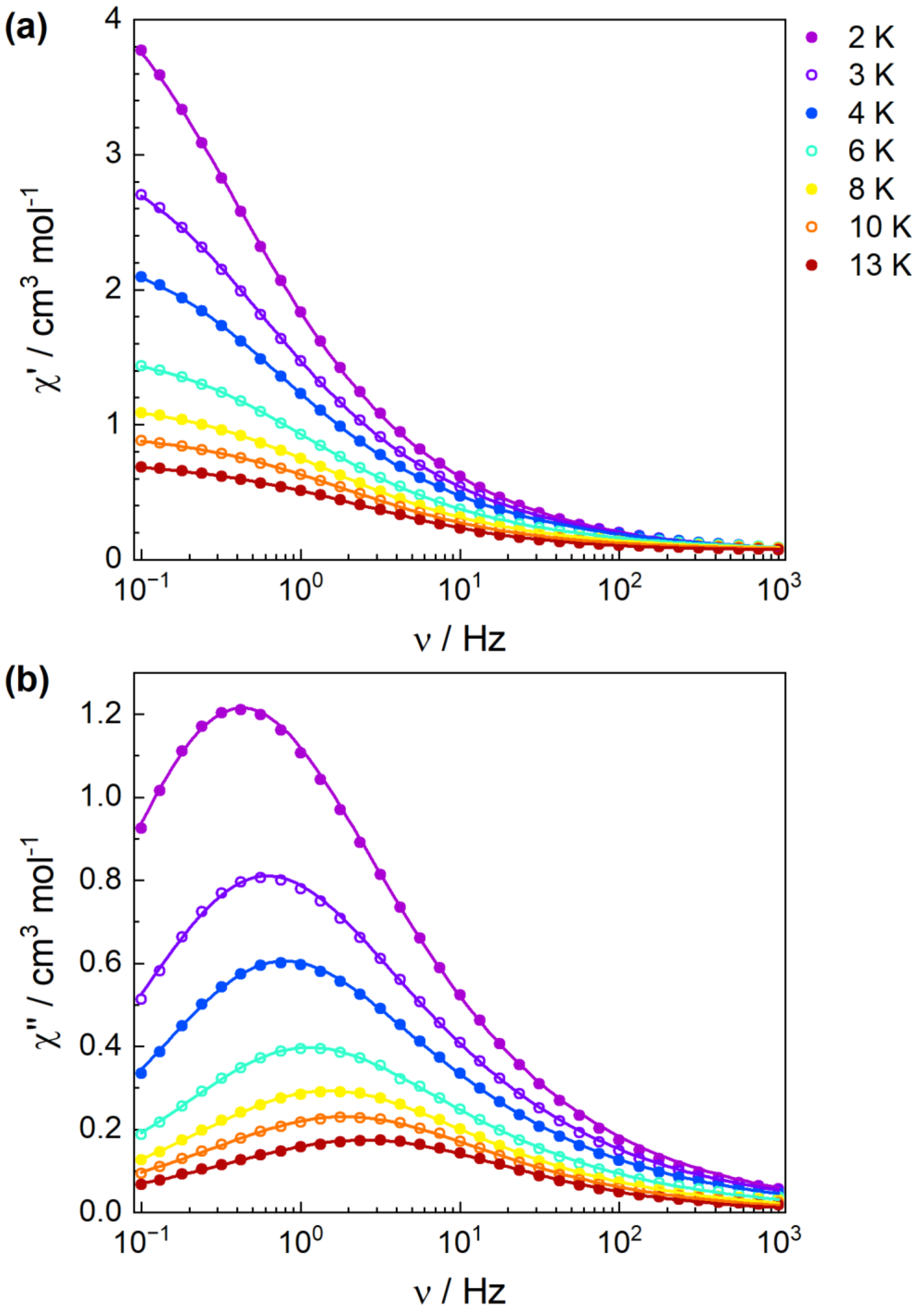

Figure S100. Fitting of the ac frequency dependence of the (a) in-phase (χ′) and (b) out-of-phase (χ″) components of the ac susceptibility for a 200 mM frozen solution of **1-Dy** in fluorobenzene (2–16 K) in zero dc field to Havriliak-Negami model.

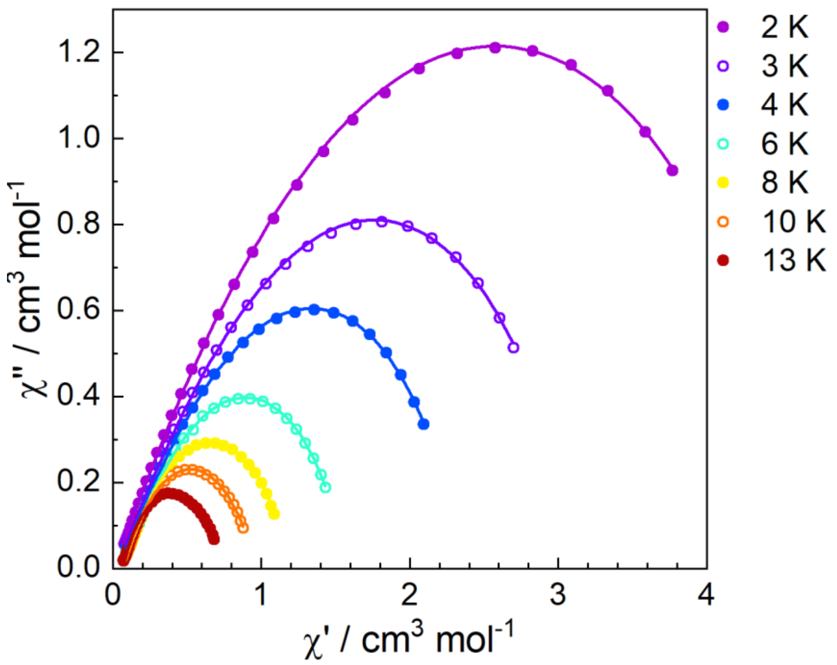

Figure S101. Cole-Cole plot showing fitting of ac data for 200 mM frozen solution of **1-Dy** in fluorobenzene (2–13 K) in zero dc field to Havriliak-Negami model.

Table S9. Best fit parameters to the Havriliak-Negami model for 200 mM frozen solution of **1-Dy** in fluorobenzene in zero dc field.

**T (K)**	**τ (s)**	**χ_S (cm^3 mol^{-1})**	**χ_T (cm^3 mol^{-1})**	**α**	**γ**
2	5.72850E-01	0.01001	4.75035	0.3308	0.7284
3	4.44912E-01	0.02301	3.14835	0.2959	0.6516
4	3.56140E-01	0.03738	2.35697	0.2846	0.6338
6	2.30695E-01	0.05765	1.57645	0.3023	0.6821
8	1.48240E-01	0.06641	1.19181	0.3392	0.7864
10	9.56223E-02	0.06858	0.96249	0.3777	0.9200
13	5.35059E-02	0.06648	0.74819	0.4164	1.1179

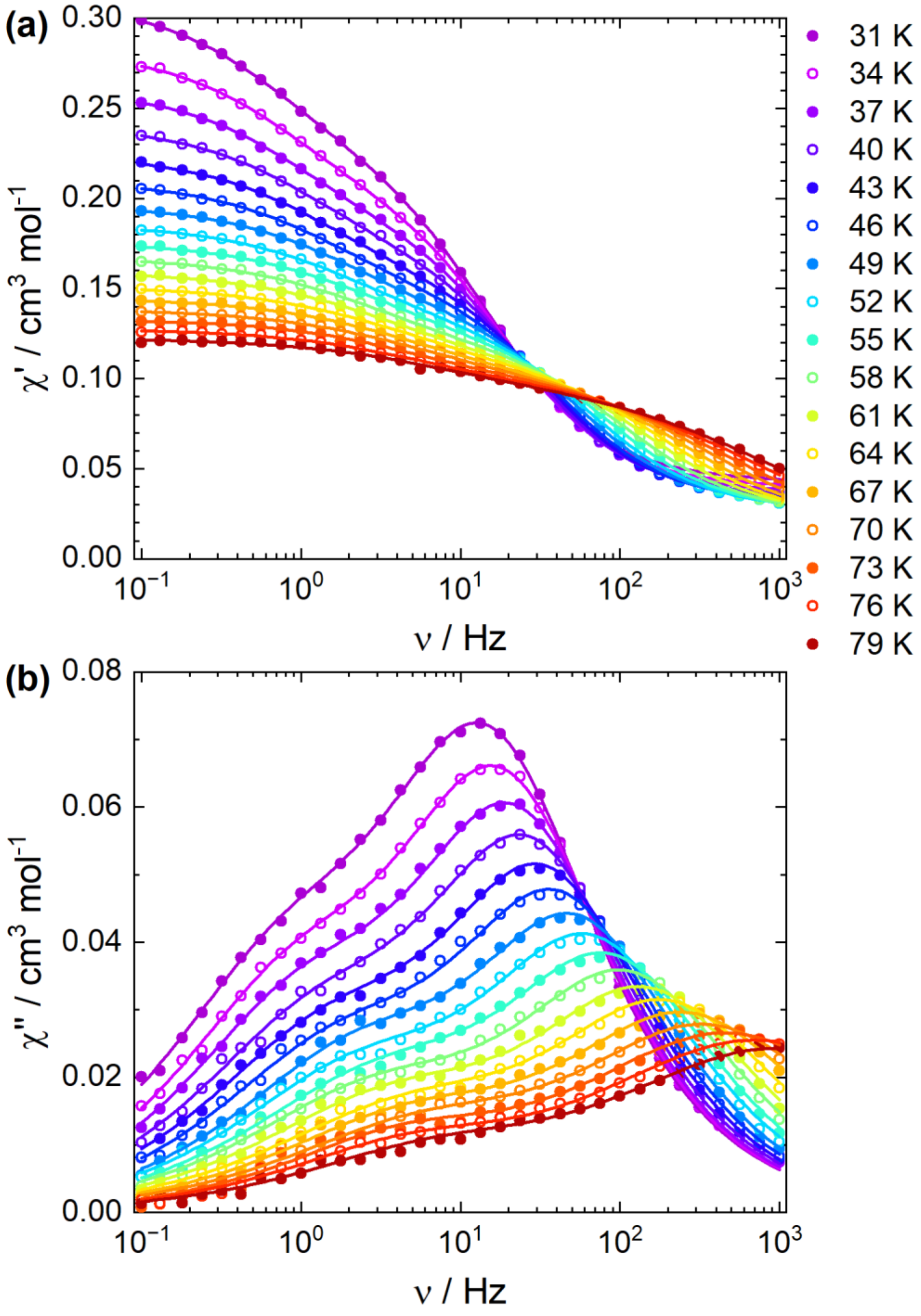

Figure S102. Fitting of the ac frequency dependence of the (a) in-phase (χ′) and (b) out-of-phase (χ″) components of the ac susceptibility for a 200 mM frozen solution of **1-Dy** in fluorobenzene (31–79 K) in dc zero field to the double generalized Debye model.

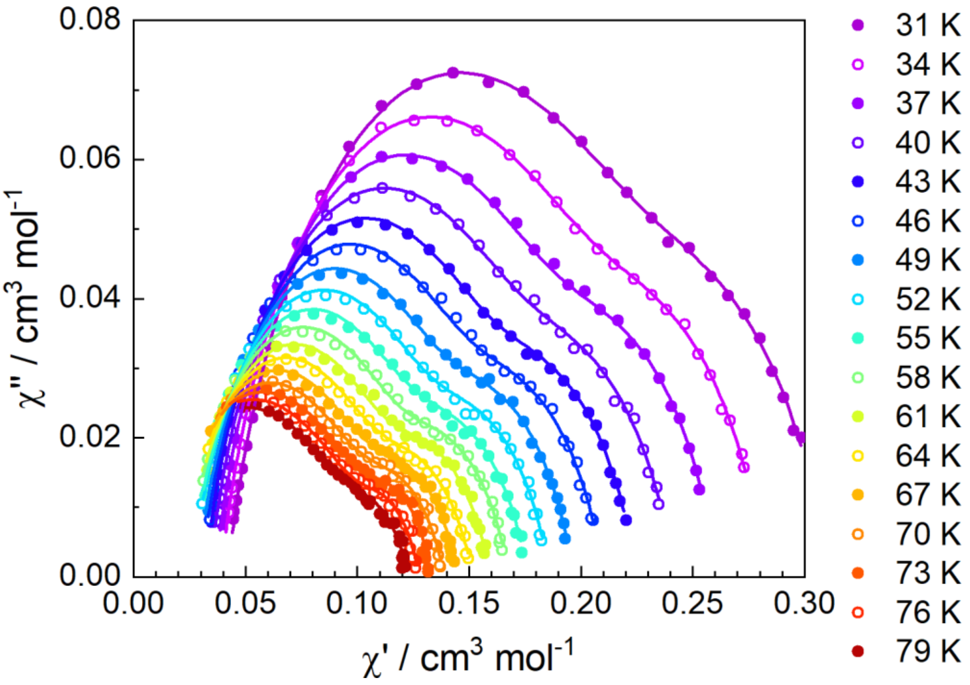

Figure S103. Cole-Cole plot showing fitting of ac data for 200 mM frozen solution of **1-Dy** in fluorobenzene (31–79 K) in dc zero field to the double generalized Debye model.

Table S10. Best fit parameters to the double generalised Debye model for 200 mM frozen solution of **1-Dy** in fluorobenzene in zero dc field.

**T / K**	$\boldsymbol{\tau_1}$ **/ s**	$\boldsymbol{\tau_2}$ **/ s**	$\Delta\chi_1$ / **cm^3 mol^{-1}**	$\Delta\chi_2$ / **cm^3 mol^{-1}**	$\Delta\chi_{total}$ / **cm^3 mol^{-1}**	$\boldsymbol{\alpha_1}$	$\boldsymbol{\alpha_2}$
31	1.04415E-2	2.25283E-1	0.17777	0.09108	0.04179	0.2058	0.2689
34	8.60700E-3	1.96454E-1	0.16179	0.08204	0.0388	0.1980	0.2544
37	7.10859E-3	1.74266E-1	0.14764	0.07718	0.03625	0.1933	0.2556
40	5.73667E-3	1.38529E-1	0.13351	0.07446	0.03377	0.1897	0.2812
43	4.59411E-3	1.19845E-1	0.12339	0.06979	0.03159	0.1869	0.2768
46	3.74834E-3	1.01594E-1	0.11471	0.0656	0.02994	0.1889	0.2844
49	2.89406E-3	7.93483E-2	0.10535	0.06292	0.02842	0.1853	0.2807
52	2.35834E-3	7.74748E-2	0.10332	0.05675	0.02588	0.2065	0.2887
55	1.76599E-3	5.75630E-2	0.09354	0.0579	0.02501	0.2010	0.3189
58	1.40002E-3	5.97924E-2	0.09377	0.05141	0.02268	0.2268	0.3098
61	1.04517E-3	4.70191E-2	0.08731	0.05042	0.02195	0.2300	0.3266
64	7.73812E-4	3.78702E-2	0.08515	0.04708	0.01955	0.2492	0.3441
67	5.78735E-4	3.56011E-2	0.08622	0.04225	0.0166	0.2792	0.3314
70	4.23175E-4	3.12863E-2	0.08675	0.03844	0.01369	0.3127	0.3415
73	3.19045E-4	3.97106E-2	0.09895	0.02783	0.00621	0.3876	0.2726
76	2.13724E-4	3.70496E-2	0.10352	0.02416	0.00000	0.4279	0.2867
79	1.56363E-4	2.63243E-2	0.09881	0.02380	0.00000	0.4313	0.3224

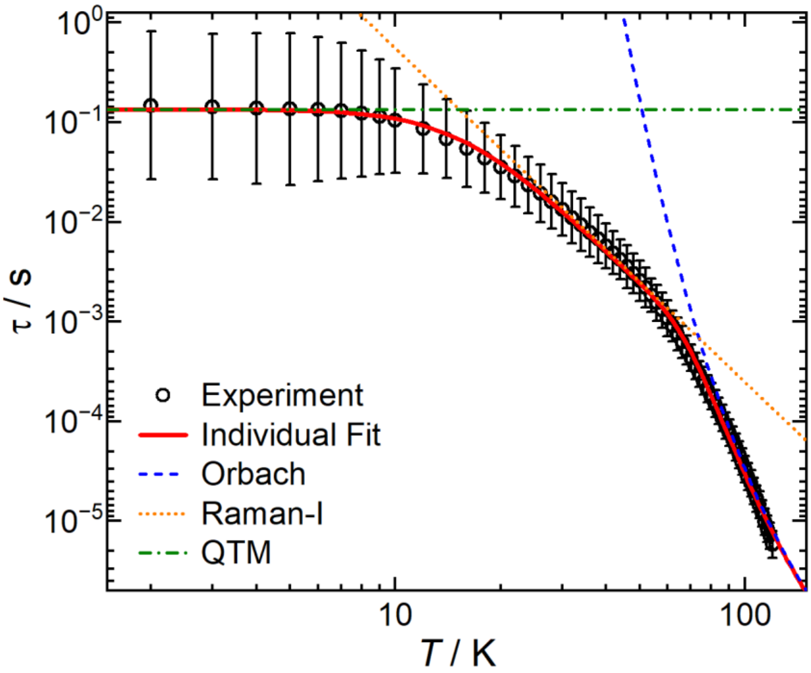

Figure S104. Temperature dependence of the magnetic relaxation time (τ) of **1-Dy** in zero dc field. Bars denote ESDs of distribution of times from the generalized Debye model.[3,4] Individual fit is of the zero dc field average relaxation times to Eqn 1, without errors.

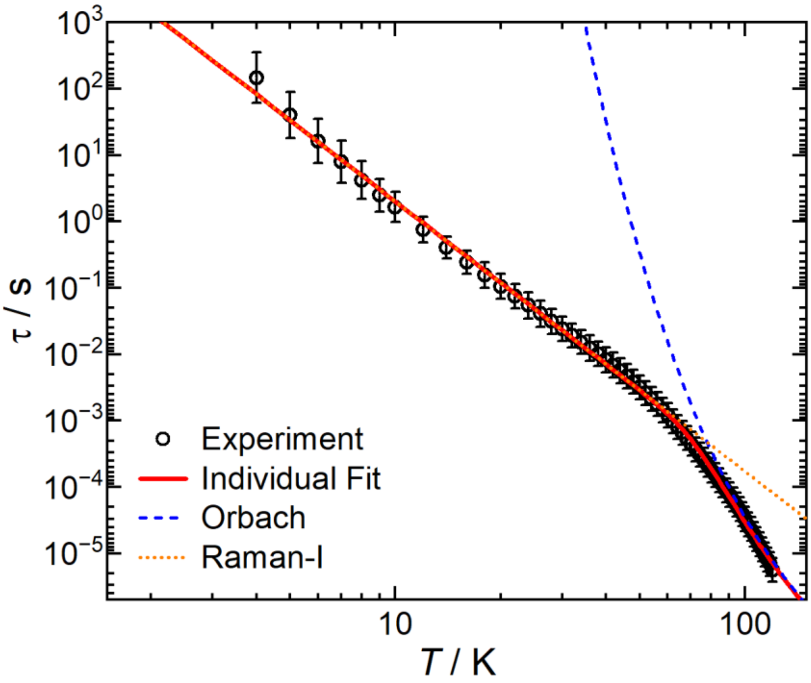

Figure S105. Temperature dependence of the magnetic relaxation time (τ) of **1-Dy** in 0.08 T dc field. Bars denote ESDs of distribution of times from the generalized Debye model.[3,4] Individual fit is of the 0.08 T field average relaxation times to Eqn 1 (τ_{QTM}^{-1} = 0), without errors.

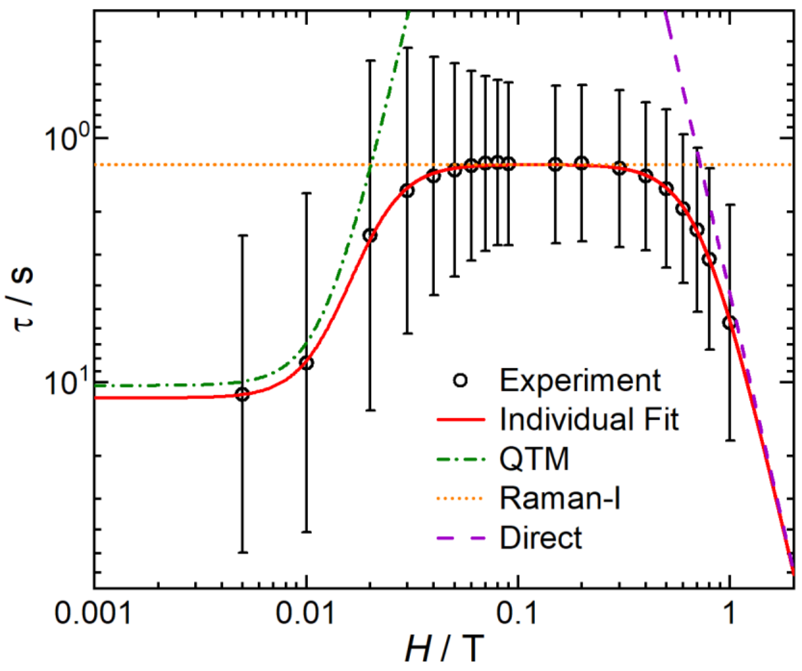

Figure S106. Field dependence of the magnetic relaxation time (τ) of **1-Dy** at 12 K. Bars denote ESDs of distribution of times from the generalized Debye model.[3,4] Individual fit is of the 12 K average relaxation times to Eqn 2, without errors.

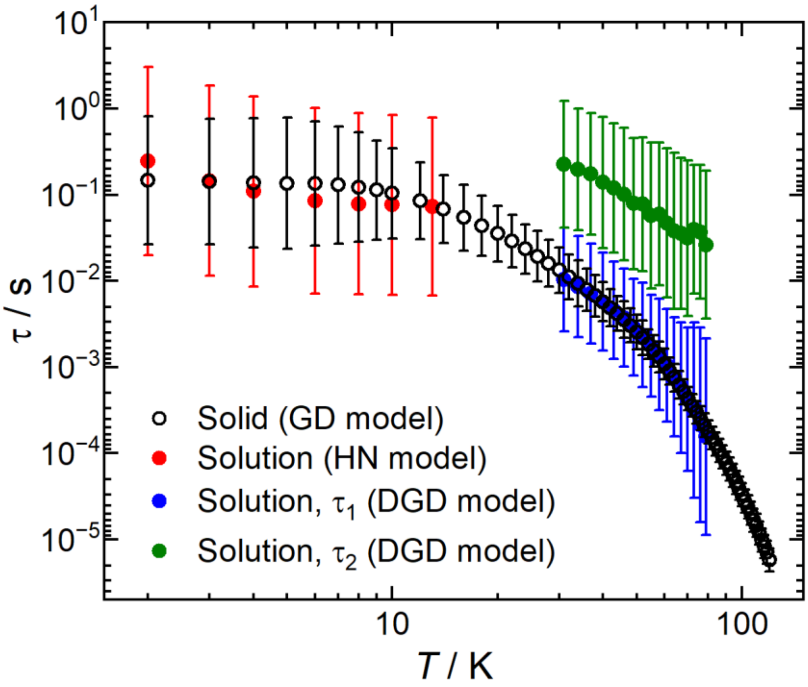

Figure S107. Comparison of relaxation times for **1-Dy** as a solid suspended in mineral oil in a polypropylene bag using the generalized Debye model (GD, black) and as 200 mM frozen solution in fluorobenzene using the Havriliak-Negami model (HN, red) or the double generalized Debye model (DGD, blue – short time, green – long time) to extract relaxation times. Bars denote ESDs of distribution of rates from the appropriate model.

8. CASSCF-SO calculations

Table S11. Electronic structure of **1-Dy** calculated with the crystal field parameters obtained from CASSCF-SO using the solid-state geometry of **1-Dy** in zero-field. Each row corresponds to a Kramers doublet.

Energy (cm^{-1})	Energy (K)	g_x	g_y	g_z	Angle[a] (deg)	Wavefunction	$<J_z>$
0.00	0.00	0.0	0.0	19.9	--	99.6% |± 15/2⟩	±7.5
427.96	615.74	0.0	0.0	16.9	0.8	98% |± 13/2⟩ + 2% |± 9/2⟩	±6.5
823.47	1184.74	0.1	0.1	14.0	1.6	94% |± 11/2⟩ + 5% |± 7/2⟩	±5.4
1149.15	1653.38	0.7	0.7	11.1	5.3	88% |± 9/2⟩ + 2% |± 13/2⟩ + 9% |± 5/2⟩	±4.3
1385.01	1992.73	1.3	2.6	8.2	10.6	76% |± 7/2⟩ + 5% |± 11/2⟩ + 1% |± 9/2⟩ + 1% |± 5/2⟩ + 15% |± 3/2⟩ + 2% |± 1/2⟩ + 1% |∓ 1/2⟩	±3.1
1531.01	2202.80	4.2	5.2	8.7	83.7	54% |± 5/2⟩ + 7% |± 9/2⟩ + 1% |± 7/2⟩ + 4% |± 3/2⟩ + 23% |± 1/2⟩ + 4% |∓ 1/2⟩ + 3% |∓ 3/2⟩ + 4% |∓ 7/2⟩	±1.7
1638.24	2357.08	1.2	2.3	15.0	87.5	46% |± 3/2⟩ + 12% |± 7/2⟩ + 4% |± 5/2⟩ + 1% |± 1/2⟩ + 14% |∓ 1/2⟩ + 2% |∓ 3/2⟩ + 19% |∓ 5/2⟩ + 1% |∓ 7/2⟩ + 2% |∓ 9/2⟩	±0.6
1709.26	2459.26	0.2	0.5	19.3	87.9	39% |± 1/2⟩ + 1% |± 7/2⟩ + 9% |± 5/2⟩ + 16% |± 3/2⟩ + 15% |∓ 1/2⟩ + 14% |∓ 3/2⟩ + 4% |∓ 5/2⟩ + 2% |∓ 7/2⟩	±0.2

[a] The angle between the g_z value of the excited Kramers doublet and the ground Kramers doublet.

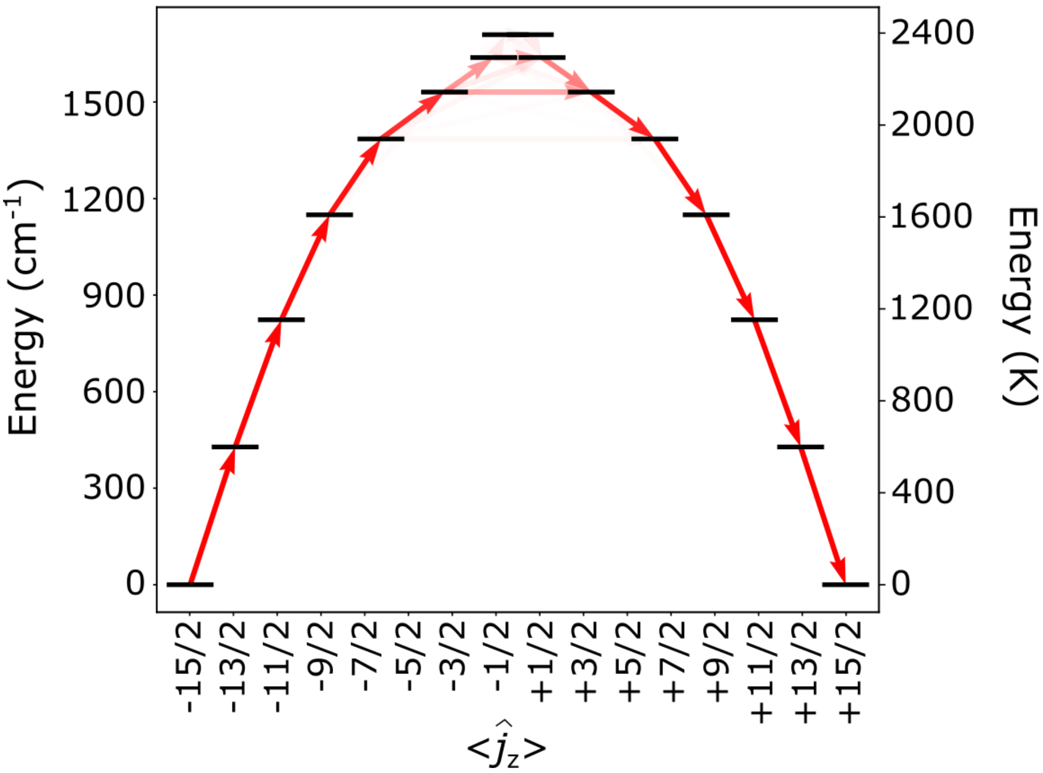

Figure S108. Energy barrier to magnetic relaxation for a model of **1-Dy**. Electronic states from CASSCF-SO calculations, labelled with their dominant m_J composition in the J = 15/2 basis. Arrows represent the Orbach relaxation pathway, where the opacity of the arrows is proportional to the transition probability approximated with the average matrix elements of magnetic moment connecting the states, $\gamma_{ij} = (1/3)[|<i|\mu_x|j>|^2 + |<i|\mu_y|j>|^2 + |<i|\mu_z|j>|^2]$, normalized from each departing state and commencing from |–15/2>.

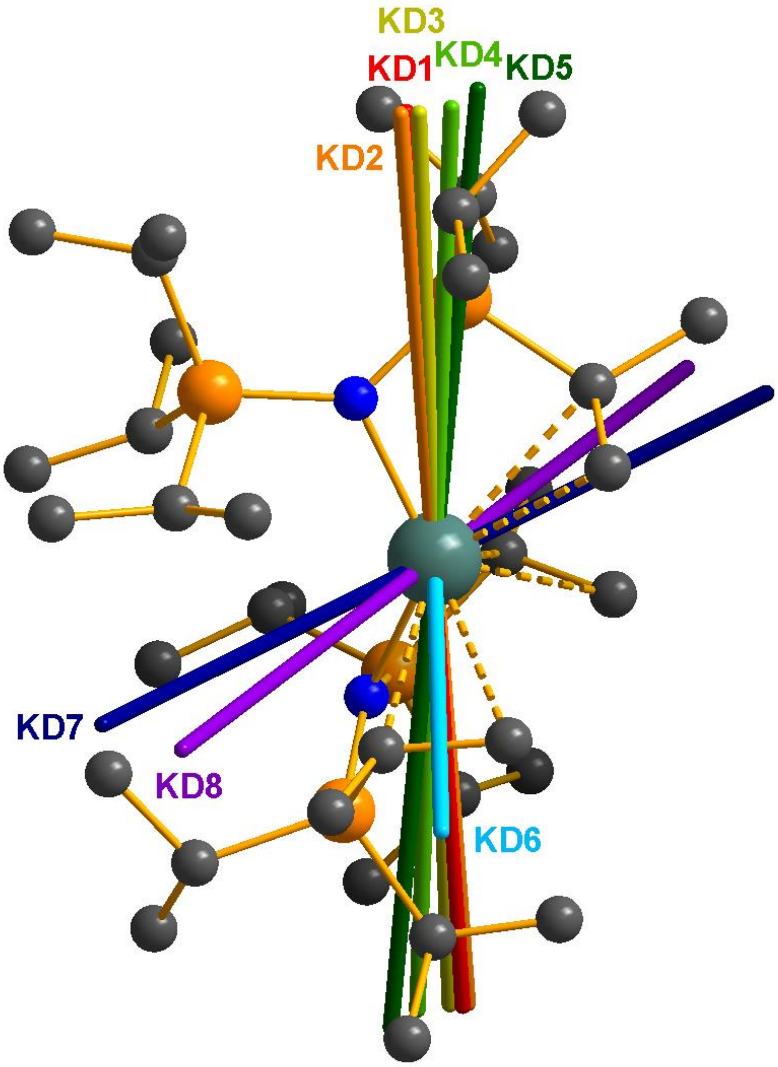

Figure S109. Structure of **1-Dy** cation with overlaid g_z vectors for Kramer's doublets (KD1–KD8) calculated with CASSCF-SO. Hydrogen atoms are omitted for clarity.

9. References

(1) Chilton, N. F.; Goodwin, C. A. P.; Mills, D. P.; Winpenny, R. E. P. The First Near-Linear Bis(Amide) f-Block Complex: A Blueprint for a High Temperature Single Molecule Magnet. *Chem. Commun.* **2015**, *51*, 101–103. https://doi.org/10.1039/C4CC08312A.

(2) Gransbury, G. K.; Corner, S. C.; Kragskow, J. G. C.; Evans, P.; Yeung, H. M.; Blackmore, W. J. A.; Whitehead, G. F. S.; Vitorica-Yrezabal, I. J.; Oakley, M. S.; Chilton, N. F.; Mills, D. P. AtomAccess: A Predictive Tool for Molecular Design and Its Application to the Targeted Synthesis of Dysprosium Single-Molecule Magnets. *J. Am. Chem. Soc.* **2023**, *145* (41), 22814–22825. https://doi.org/10.1021/jacs.3c08841.

(3) Reta, D.; Chilton, N. F. Uncertainty Estimates for Magnetic Relaxation Times and Magnetic Relaxation Parameters. *Phys. Chem. Chem. Phys.* **2019**, *21* (42), 23567–23575. https://doi.org/10.1039/C9CP04301B.

(4) Blackmore, W. J. A.; Gransbury, G. K.; Evans, P.; Kragskow, J. G. C.; Mills, D. P.; Chilton, N. F. Characterisation of Magnetic Relaxation on Extremely Long Timescales. *Phys. Chem. Chem. Phys.* **2023**, *25*, 16735–16744. https://doi.org/10.1039/D3CP01278F.
